# Hydrophilic Reduction-Resistant Spin Labels of Pyrrolidine and Pyrroline Series from 3,4-Bis-hydroxymethyl-2,2,5,5-tetraethylpyrrolidine-1-oxyl

**DOI:** 10.3390/ijms25031550

**Published:** 2024-01-26

**Authors:** Mikhail S. Usatov, Sergey A. Dobrynin, Yuliya F. Polienko, Denis A. Morozov, Yurii I. Glazachev, Sergey V. An’kov, Tatiana G. Tolstikova, Yuri V. Gatilov, Irina Yu. Bagryanskaya, Arthur E. Raizvikh, Elena G. Bagryanskaya, Igor A. Kirilyuk

**Affiliations:** 1 N. N. Vorozhtsov Novosibirsk Institute of Organic Chemistry SB RAS, Lavrentiev Ave. 9, Novosibirsk 630090, Russia; musatov@nioch.nsc.ru (M.S.U.); dobrynin@nioch.nsc.ru (S.A.D.); polienko@nioch.nsc.ru (Y.F.P.); dmorozov@nioch.nsc.ru (D.A.M.); sergey.ankov42@gmail.com (S.V.A.); tolstiktg@nioch.nsc.ru (T.G.T.); gatilov@nioch.nsc.ru (Y.V.G.); bagryan@nioch.nsc.ru (I.Y.B.); arturaiz@nioch.nsc.ru (A.E.R.); egbagryanskaya@nioch.nsc.ru (E.G.B.); 2 Department of Natural Sciences, Novosibirsk State University, Pirogova Str. 1, Novosibirsk 630090, Russia; 3 Voevodsky Institute of Chemical Kinetics and Combustion SB RAS, Institutskaya 3, Novosibirsk 630090, Russia; glaza@kinetics.nsc.ru; 4 Department of Physics, Novosibirsk State University, Pirogova Str. 1, Novosibirsk 630090, Russia

**Keywords:** reduction-resistant nitroxide, spin label, spin probe, EPR, hyperfine coupling

## Abstract

Highly resistant to reduction nitroxides open new opportunities for structural studies of biological macromolecules in their native environment inside living cells and for functional imaging of pH and thiols, enzymatic activity and redox status in living animals. 3,4-Disubstituted nitroxides of 2,2,5,5-tetraethylpyrrolidine and pyrroline series with a functional group for binding to biomolecules and a polar moiety for higher solubility in water and for more rigid attachment via additional coordination to polar sites were designed and synthesized. The EPR spectra, lipophilicities, kinetics of the reduction in ascorbate-containing systems and the decay rates in liver homogenates were measured. The EPR spectra of all 3,4-disubstituted pyrrolidine nitroxides showed additional large splitting on methylene hydrogens of the ethyl groups, while the spectra of similar pyrroline nitroxides were represented with a simple triplet with narrow lines and hyperfine structure of the nitrogen manifolds resolved in oxygen-free conditions. Both pyrrolidine and pyrroline nitroxides demonstrated low rates of reduction with ascorbate, pyrrolidines being a bit more stable than similar pyrrolines. The decay of positively charged nitroxides in the rat liver homogenate was faster than that of neutral and negatively charged radicals, with lipophilicity, rate of reduction with ascorbate and the ring type playing minor role. The EPR spectra of *N*,*N*-dimethyl-3,4-bis-(aminomethyl)-2,2,5,5-tetraethylpyrrolidine-1-oxyl showed dependence on pH with *pK_a_* = 3, Δ*a*_N_ = 0.055 mT and Δ*a*_H_ = 0.075 mT.

## 1. Introduction

Nitroxides (aminoxyl radicals) are widely used as molecular probes and labels for the investigation of complex molecular systems using various methods of EPR and NMR spectroscopy and imaging [1,2,3,4,5,6,7,8,9,10]. Biophysics and structural biology are the areas where application of nitroxide-based spectroscopic methods have been especially fruitful. Special approaches and techniques have been developed for structural studies of biological macromolecules in their native environment inside living cells [2,11] and for functional imaging of pH and thiols [2,12], enzyme activity [13] and redox status [14] in living animals. The stability of the nitroxide radical is of crucial importance for these applications. Early studies showed that reduction of a nitroxide group to hydroxylamine one with enzymatic systems and low-molecular biogenic antioxidants is a major pathway of nitroxide decay in biological systems [15]. To slow down a radical decay, so-called “sterically shielded” pyrroline and pyrrolidine nitroxides with four ethyl (or more bulky alkyl) substituents adjacent to the N-O group were designed [16,17]. These nitroxides showed much higher stability in biological samples and in cells than conventional tetramethyl-substituted nitroxides [18,19,20,21]. A number of reduction-resistant spin labels for in-cell applications have been prepared from these nitroxides [20,21,22]. However, bulky alkyl substituents make these spin labels lipophilic and poorly soluble in water. Stock solutions were usually prepared in DMSO or in water–DMSO mixtures, see for example [20,23]. Recent studies revealed that an alternative metabolic pathway implying cytochrome P450 activity may significantly contribute to the decay of sterically shielded nitroxides in subcellular systems [24,25]. These data coincide with our observations that decay of 2,2,5,5-tetraethylpyrrolidine nitroxides in homogenates of liver and kidney, which are known to be rich of cytochrome P450 enzymes, proceeds much faster than in homogenates of brain, heart or in blood [22]. According to some estimates, hydrophilic nitroxides, with a negative charge and/or minimal hydrophilicity are required to slow down transformation by P450 enzymes, either via reductive or oxidative metabolism [24].

**Scheme 1:**
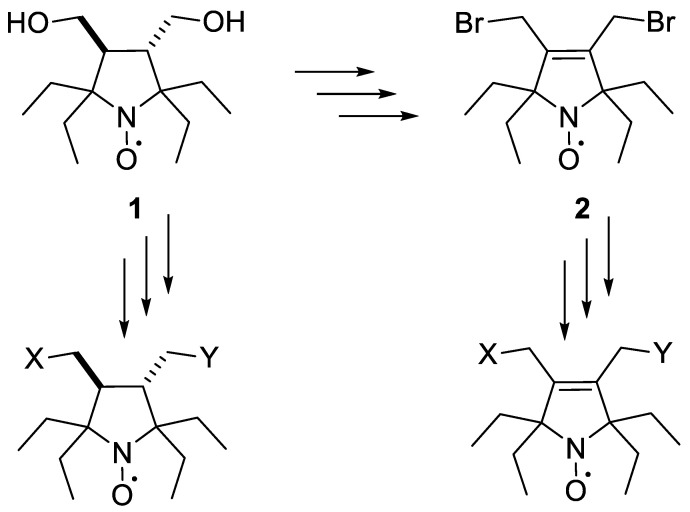
General approach to the synthesis of hydrophilic reduction-resistant spin labels.

We recently described convenient procedures for synthesis of 3,4-bis-hydroxymethyl-2,2,5,5-tetraethylpyrrolidine-1-oxyl (**1**) (Scheme 1) [26,27]. Pyrrolidine nitroxide **1** can be converted into pyrroline derivatives, e.g., **2** [28]. However, synthetic potential of **1** and **2** was not seriously studied yet. It should be noted that similar bifunctional derivatives of 2,2,5,5-tetramethyl-substituted pyrrolidine or pyrroline nitroxides attracted much attention, although they are not as readily available, see for example [29,30,31,32]. Spin labels of pyrroline series with additional functional groups in a neighboring position of the heterocycle are known for especially rigid binding to the target biomolecules allowing for more accurate distance measurements using pulsed EPR methods [33,34,35].

The aim of this work was to find a convenient synthetic approach to 2,2,5,5-tetraethyl-3,4-disubstituted pyrrolidine and pyrroline nitroxides with two different functional groups: one for selective binding to biopolymers or other target molecules (X) and a polar charged moiety that makes the nitroxide hydrophilic and water soluble (Y). We have focused on the preparation of hydrophilic analogs of the known reduction-resistant maleimide spin labels, which are used for selective binding to thiol groups of cysteine residues [20,21], and nitroxide azides, which can be used inside the cells for bioorthogonal spin labeling of proteins modified with unnatural alkyne-containing amino acids using copper (I)-catalyzed azide–alkyne cycloaddition (CuAAC) [36] or strain-promoted azide–alkyne cycloadditions (SPAAC) [37]. Since **1** is a readily available compound, novel hydrophilic nitroxides can be produced on a large scale not only for SDSL-EPR studies but also for the development of specific probes/contrast agents for in vivo EPRI/OMRI/MRI studies. The parameters of EPR spectra of the new nitroxides, their lipophilicities and decay in liver homogenate and in model ascorbate-containing systems were studied.

## 2. Results and Discussion

### 2.1. Synthesis of the Hydrophilic Nitroxides

**Scheme 2:**
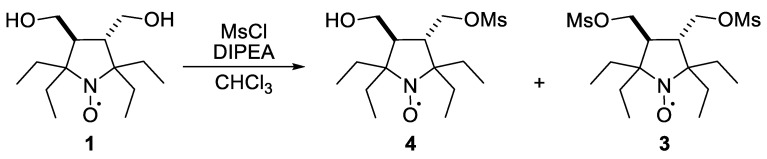
The reaction of **1** with MsCl in the presence of DIPEA.

**Figure 1:**
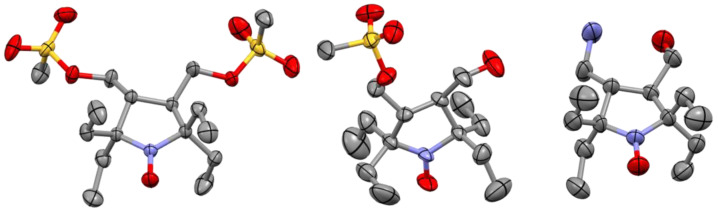
The structure of **3**, **4** and **6** according to single-crystal X-ray diffraction data (hydrogen atoms are not shown).

To convert a symmetric structure, such as **1**, into heterofunctional spin labels one should find a convenient way to modify only one of the two identical functional groups with a satisfactory yield. Reactions on one of two hydroxyl groups in **1** seems to be the shortest pathway to heterobifunctional derivatives of 2,2,5,5-tetraethylpyrrolidine-1-oxyl. The subsequent selective modification of the substituents in the position 3 and 4 can be used to prepare various derivatives. We have showed earlier that the reaction of **1** with excess of methanesulfonyl chloride (MsCl) in the presence of diisopropylethylamine (DIPEA) afforded **3** (Scheme 2) [28], and the latter was used in the synthesis of **2**. Notably, we did not observe any significant conversion in the absence of a base, while the reaction with MsCl in the presence of excess of the base caused accumulation of dark-colored products. Therefore, we used slow addition of DIPEA to control the reaction in the presence of excess of MsCl. The addition of a smaller amount of reagents gives a mixture of di- and monomesylated products **3** and **4**. The optimal preparative yield of **4** 64–68% (75–80% with conversion ca. 85%) was achieved upon slow addition of DIPEA (1.4 eq.) to a solution of **1** and MsCl (1.58 eq.). The higher excess of the reagents required for nearly quantitative conversion of **1** with yields of **3** and **4** are 35 and 55%, correspondingly. Both **3** and **4** were isolated as crystalline compounds. Structure assignments were done on the basis of NMR spectra after nitroxide reduction with Zn/CF_3_COOH [22], IR spectra, element analyses (see Section 3 and Supplementary Materials pp. S4, S10) and single crystal X-ray data (Figure 1). An unexpectedly high yield of **4** may result from stabilization of anionic form of **1** via hydrogen bonding with the neighboring hydroxy group, which makes the rate of mesylation of **1** much higher than that of **4**.

**Scheme 3:**
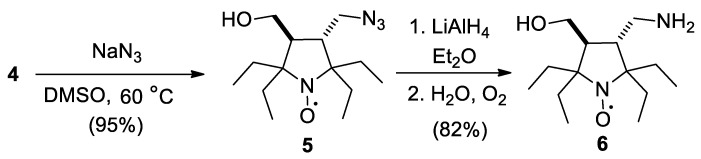
Synthesis of **6**.

Mesyloxy derivatives are convenient precursors for a variety of spin labels. Heating of **4** with an excess of sodium azide in DMSO afforded **5** with nearly quantitative yield (Scheme 3). Azido group was then reduced with LiAlH_4_ to give aminoalcohol **6** which was isolated as crystalline solid. The structure of **6** was confirmed by IR and NMR spectra and single crystal X-ray analyses data (Figure 1 and Supplementary Materials pp. S5, S12).

**Scheme 4:**
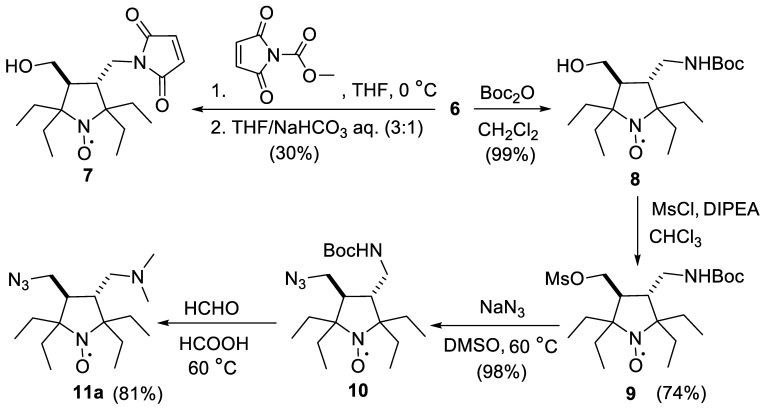
Conversion of **6** into spin label **7** and synthesis of **11a**.

A reaction with N-methoxycarbonyl maleimide (NMCM) was chosen to convert amine **6** into corresponding spin label (Scheme 4). The use of this reagent made it possible to keep the hydroxy group intact, although the yield of spin label **7** was low. In this and all subsequent similar syntheses of maleimide spin labels, we used a modified protocol based on literature methods [38,39]. Primary amine was slowly added dropwise to a solution of NMCM in dry THF at 0 °C under argon, and then the reaction mixture was diluted with a saturated aqueous solution of NaHCO_3_. This two-step procedure prevents the addition of unreacted amine to the maleimide formed, since the elimination of methylcarbamate to form maleimide occurs in the second stage, when the amine has already been consumed. 

**Figure 2:**
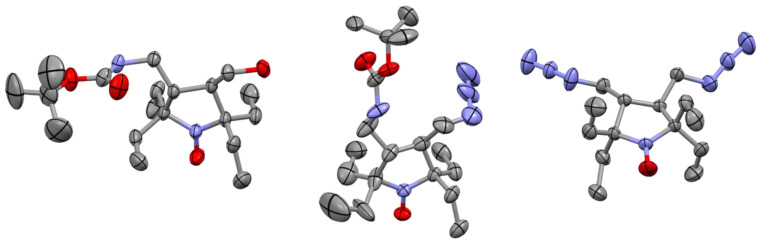
The structure of **8**, **10** and **12a** according to single-crystal X-ray diffraction data (hydrogen atoms are not shown).

Despite the presence of a hydrophilic hydroxy group, radicals **5** and **7** are still poorly soluble in water. The introduction of quaternary ammonium groups, such as trimethylammonium, is a simple and effective way to make a compound highly hydrophilic. It is more convenient to introduce such groups at the final steps of synthesis. For this reason, amino group in **6** was protected with *tert*-butyloxycarbonyl moiety to give **8**. The latter was carefully treated with methanesulfonyl chloride in basic conditions (excess of DIPEA) to give **9** and subsequent heating with NaN_3_ according to the procedure described above afforded **10**. The structures of **8** and **10** were assigned on the basis of IR spectra and HRMS (see Section 3 and Supplementary Materials pp. S11–S12) and confirmed with single-crystal X-ray analysis data (Figure 2). Under Eschweiler–Clarke reaction conditions deprotection and alkylation occur with formation of **11a** as a single product.

**Scheme 5:**
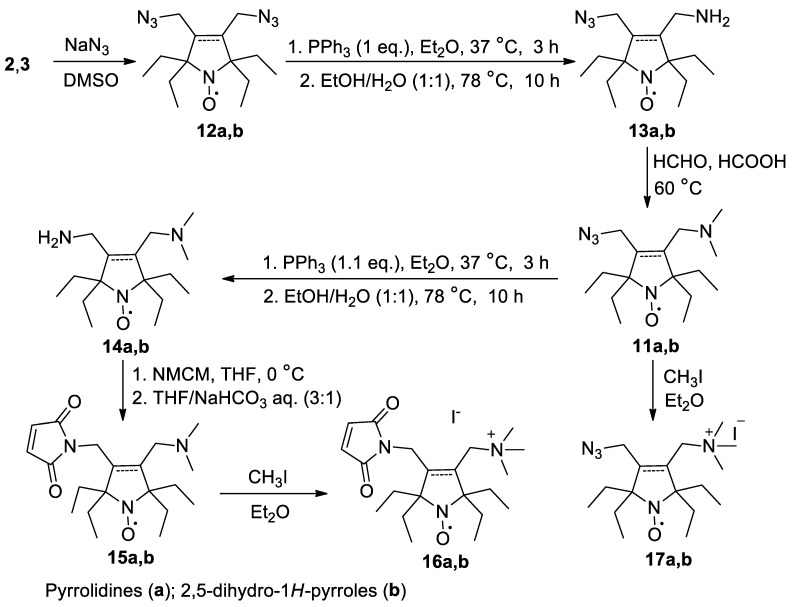
Synthesis of cationic 2,5-dihydro-1*H*-pyrrole and pyrrolidine spin labels.

We have found that **11a** can also be prepared from **3** (Scheme 5) with a higher yield and in fewer steps (45% overall yield from **1** in 4 steps versus 35–36% in 7 steps via Scheme 1, Scheme 2, Scheme 3 and Scheme 4). It is noteworthy that a similar radical of the pyrroline series, **11b**, can also be obtained from **2** using this scheme. It is difficult to obtain a good yield of the monoazido derivative by treating **2** or **3** with sodium azide, see for example literature data for similar 2,2,5,5-tetramethyl-substituted nitroxide [40]. In contrast, the corresponding diazides **12a,b** were readily obtained by heating of **2** and **3** with excess of NaN_3_ in DMSO. These nitroxides were isolated as orange crystals with 93–99% yield and characterized with IR spectra, HRMS and element analyses (see Section 3 and Supplementary Materials pp. S11–S12). The structure of **12a** was confirmed by single crystal X-ray data (Figure 2). The subsequent Staudinger reaction with one equivalent of triphenylphosphine afforded **13a,b** in 64–73% yield. The resulting aminoazides were converted to **11a,b** by Eschweiler–Clarke alkylation.

**Figure 3:**
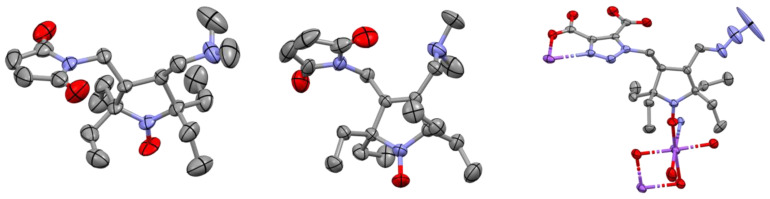
The structure of **15a,b** and **20** according to single-crystal X-ray diffraction data (hydrogen atoms are not shown).

For the preparation of maleimide spin labels from **11a,b** we used the Staudinger reaction followed by treatment with NMCM in analogy to the above procedures. The structures of **15a,b** were confirmed by single crystal X-ray analysis (Figure 3). The addition of methyl iodide to the solutions of the spin labels **11a,b** and **15a,b** in dry diethyl ether leads to precipitation of the corresponding trimethylammonium iodides **16a,b** and **17a,b**. 

**Scheme 6:**
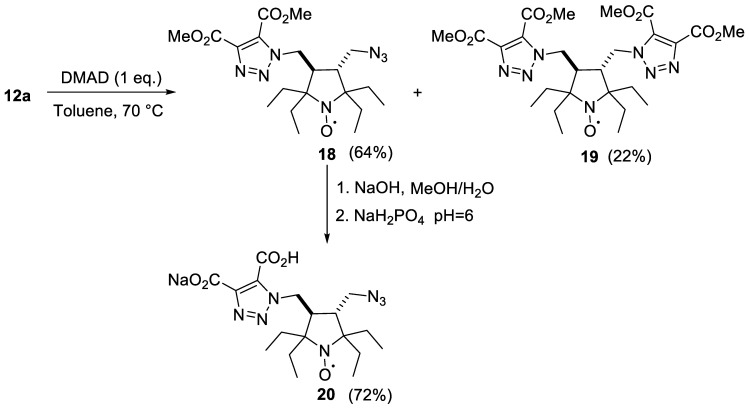
Synthesis of hydrophilic spin label **20** using Huisgen 1,3-dipolar cycloaddition reaction.

Diazide **12a** can also be converted into dianionic spin label. A Huisgen cycloaddition reaction with one equivalent of dimethyl acetylenedicarboxylate (DMAD) gave a mixture of mono- and bis-triazoles **18** and **19** (Scheme 6). After alkaline hydrolysis of the ester groups in **18**, the monosodium salt **20** was isolated. The structure of this nitroxide was confirmed by single-crystal X-ray diffraction data (Figure 3).

### 2.2. EPR Studies of the New Nitroxides

**Figure 4:**
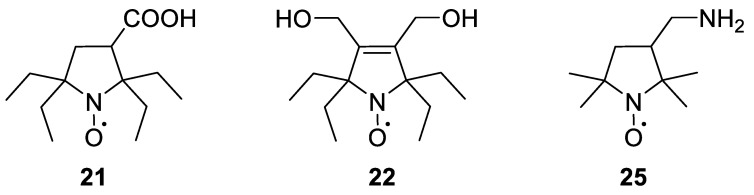
Structure of nitroxides **21**, **22** and **25**.

**Scheme 7:**
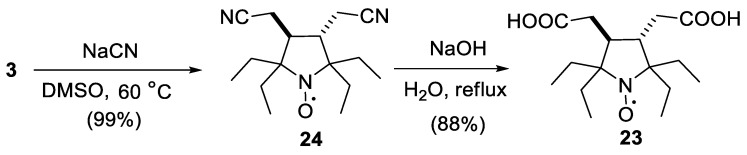
Synthesis of **23**.

The CW-EPR spectra (X-band) were recorded for a broad selection of the new radicals (see Supplementary Materials pp. S23–S26). The parameters of the EPR spectra are given in Table 1. For comparison parameters of the spectra of nitroxides **1**, 3-carboxy-2,2,5,5-tetraethylpyrrolidine-1-oxyl (**21**) [22] and 2,2,5,5-tetraethyl-3,4-bis(hydroxymethyl)-2,5-dihydro-1*H*-pyrrol-1-oxyl (**22**) [28] (Figure 4) are shown. In addition, the nitroxide **23** was synthesized for this study by heating **3** with sodium cyanide followed by alkaline hydrolysis of the cyano groups (Scheme 7).

**Figure 5:**
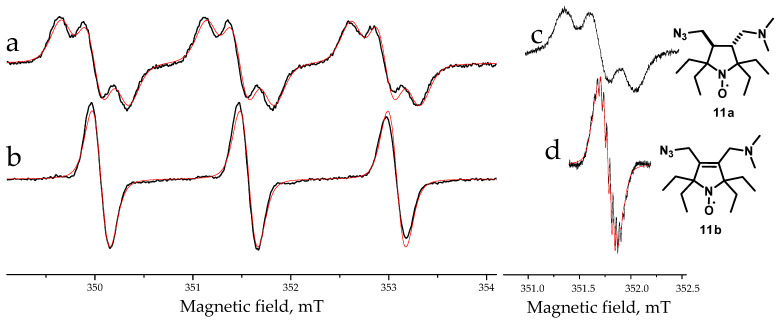
EPR spectra of **11a,b** in 50 mM phosphate buffer under aerobic conditions, **11a** (**a**) and **11b** (**b**) and in the oxygen-free solutions (middle component), **11a** (**c**) and **11b** (**d**).

The EPR spectra of 3,4-disubstituted pyrrolidine nitroxides correspond to the pattern described for **1**, with broad lines and two large additional doublet splittings on two hydrogen atoms of the methylene groups of the ethyl substituents [26,41] (Figure 5a). The value of these constants depends on the size of the neighboring substituent at position 3 or 4. For example, the introduction of the bulky maleimido group results in up to a 50% increase in one of two *a*_H_ (cf. **14a** and **15a**; **16a** and **17a**). We have showed earlier that high *hfc* with one of methylene hydrogens of ethyl group is observed in nitroxides with the pseudoaxial position of this ethyl group and CH_2_-CH_3_ bond nearly parallel to the N-O bond axis [41]. Apparently, the repulsion of the large substituent and the neighboring ethyl group (*cis* relative to each other) increases the contribution of this conformation.

In contrast, EPR spectra of the pyrroline nitroxides showed classical triplet with no additional large splittings (Figure 5b). In oxygen-free solution, hyperfine structure of the spectral lines was resolved, which is typical for the nitroxides with planar heterocycle (pyrroline, 3-imidazoline and isoindoline) (Figure 5d) [4]. The simulation gives four *a*_H_ = 0.0611 mT and four *a*_H_ = 0.0317 mT, apparently due to *hfc* with methylene hydrogens of ethyl groups.

**Figure 6:**
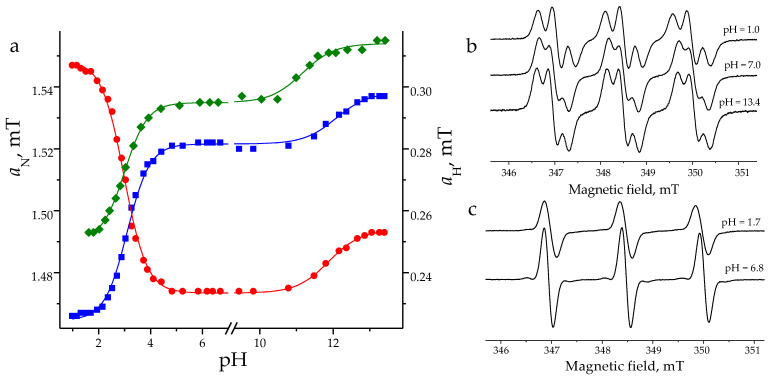
Dependence of the EPR spectra of **14a,b** on pH. Titration curves, **14a**, *a*_N_ versus pH (blue squares), *a*_H_ versus pH (red circles), **14b**, *a*_N_ versus pH (green diamonds) (**a**). The fitting with modified Henderson–Hasselbalch equation [43] is shown with solid lines. EPR spectra of **14a** at pH 1.0, 7.0 and 13.4 (**b**); of **14b** at pH 1.7 and 6.8 (**c**).

**Scheme 8:**
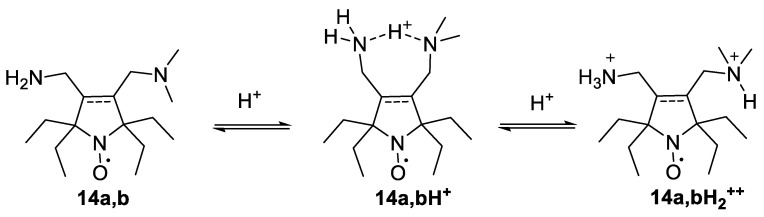
Protonation of **14a,b**.

The EPR spectra of nitroxides with ionizable groups may be dependent on pH. Similarly to that described for 3-aminomethyl-proxyl (**25**) [42] (Figure 4), spectra of 3-aminomethyl- and 3-dimethylaminomethyl nitroxides **6**, **11a,b**, **13a,b** and **15a,b** demonstrate small dependence on pH with Δ*a*_N_ ca. 0.02 mT and pK ca. 9. Titration of the nitroxides **14a** and **14b** revealed two transitions at pH ca. 3 and ca. 11 (Figure 6), which apparently correspond to two protonation steps with formation of mono- and dication. Similarly to the above pattern protonation of free bases **14a,b** to form monocations leads to minor changes in nitrogen *hfc*, Δ*a*_N_ = 0.02 mT, however more significant changes were observed upon the second protonation, Δ*a*_N_ = 0.044–0.055 mT. Surprisingly, the constants on the hydrogen atoms of the methylene fragments of ethyl groups in **14a** turned out to be more sensitive to pH changes (Figure 6). While formation of monocation **14aH^+^** leads to decrease of apparent *a*_H_ by 0.02 mT, the spectrum of **14aH_2_**^++^ (pH = 1) shows increase of the hydrogen *hfc* constant by 0.075 mT. Presumably the monocations **14aH^+^** and **14bH^+^** are stabilized due to coordination (hydrogen bonding) with the neighboring amino group forming 7-membered rings (Scheme 8).

This stabilization partly accounts for higher pK of the first protonation of **14a,b**. Formation of these cyclic structures apparently decreases the repulsion of the ethyl groups and the substituents in the position 3 and 4. A noticeable decrease of *a*_H_ upon the first protonation of **14a** confirms the assumption of the formation of these cyclic structures. In contrast, the second protonation leads to opening of the 7-membered ring and strong conformational changes because of electrostatic repulsion of the neighboring cationic groups. These changes result in pronounced growth of *a*_H_ in the spectra of **14a**. In the spectra of **14b,** the hyperfine structure of the triplet manifolds was not resolved, but growth of *a*_H_ upon the second protonation can be followed by the inhomogeneous line broadening which leads to notable decrease of the peak height in the spectrum (Figure 6c).

### 2.3. Lipophilicity

Partition in octanol-water mixtures for ionizable compounds may depend on pH, and the pH changes may result from addition of the compound. Therefore, phosphate-citrate-borate buffer (5 mM, pH 7.4) was used for K_p_ measurements to make the partition closer to physiological conditions. The data is listed in Table 1. The new set of spin labels contains highly hydrophilic nitroxides, such as the positively charged spin labels **16a,b** and **17a,b** and negatively charged **20**, which were not detected in octanol after shaking with buffer. Although monoamino derivatives **11** and **13** mainly exist in protonated form at pH 7.4, they still remain lipophilic and, therefore, should permeate cells. Amino groups are known to provide higher retention/intracellular accumulation due to transmembrane potential or ion-trapping effect [44]. This effect was exploited in design of nitroxide contrast agents for MRI and radioprotectors [45]. Cellular accumulation of spin labels like **11** and **13** may help to increase efficacy spin labeling using CuAAC or SPAAC.

### 2.4. Reduction Rates in Ascorbate-Containing Systems

Rate constants of the nitroxides decay in the ascorbate-containing systems are given in Table 1. All the pyrrolidine radicals demonstrated very high stability to reduction, exceeding that of **21**. Reduction rates of pyrroline nitroxides are always expectedly higher than those of similar nitroxides of pyrrolidine series, but they were never higher than that of **21** by more than 40%.

### 2.5. Decay in the Rat Liver Homogenates

**Figure 7:**
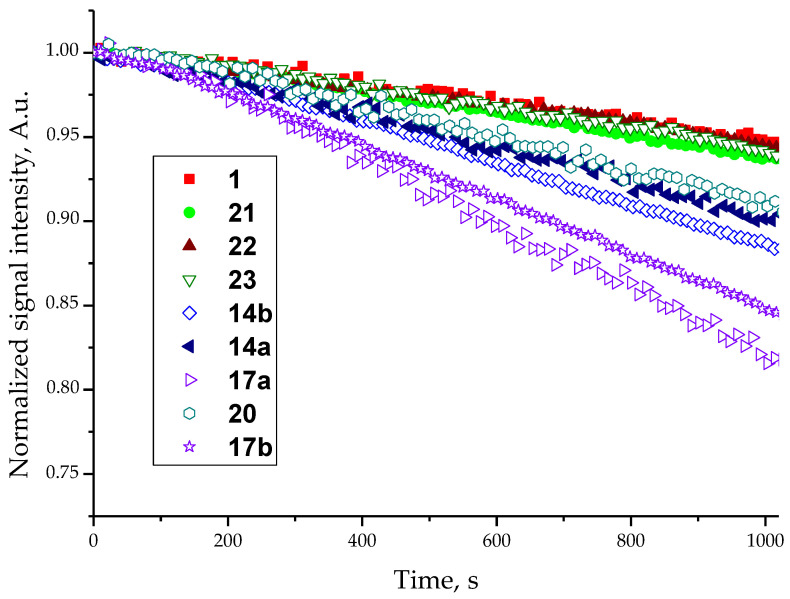
Decay of nitroxides in rat liver homogenate.

For this research we used a selection of nitroxides, containing cationic groups **17a,b**, ionizable groups existing at pH 7.4 in cationic **14a,b** and anionic **20**, **21**, **23** forms, and non-ionizable nitroxides with hydroxyl groups **1**, **22**. We avoided using maleimido derivatives because they could react with proteins of the homogenate, therefore, the results would be hard to interpret. The nitroxide solutions were mixed with rat liver homogenate at 37 °C and decay of the EPR signal was followed. The kinetics of decay are shown in Figure 7.

Surprisingly, the decay rates showed no correlation with the reduction rates with ascorbate, with the lipophilicity values or with the nitroxide heterocyclic ring type. The compounds can be divided into three categories in accordance with the type of the functional groups. The decay of cationic nitroxides and amino derivatives was significantly faster than that of carboxylic acids and diols. In our previous study, we showed that the decay of positively charged 2,2,5,5-tetraethylpyrrolidine-1-oxyls in homogenates of kidney and liver proceeds much faster than in blood and in homogenates of brain and heart [22]. Therefore, a relatively high rate of decay of cationic nitroxides in liver homogenates does not mean that they cannot be used as spin labels in living cells, since the cells normally used in such studies do not have such a high activity towards xenobiotics. However, the above data show that negatively charged or non-charged polar nitroxides may perform better in EPR/NMR imaging studies in vivo. This coincides with the conclusions of the recent paper by Peyrot, et al. [24]. Despite much similarity of our results to literature data, we are far from the assignment of the observed decay of nitroxides to certain enzyme activity and from elucidation of the decay mechanism without special investigation.

## 3. Materials and Methods

### 3.1. General Information

The compounds **1** [26,27] and **2** [28] were prepared according to the literature. The IR spectra were recorded on a Bruker Vector 22 FT-IR spectrometer (Bruker, Billerica, MA, USA) in KBr pellets (1:150 ratio) or in neat samples (see the Supplementary Materials in this article pp. S10–S18) and are reported in wave numbers (cm^−1^). UV spectra were acquired on a HP Agilent 8453 spectrometer (Agilent Technologies, Santa Clara, CA, USA) in ethanol solutions (concentration~10^−4^ M). ^1^H NMR spectra were recorded on a Bruker AV 300 (300.132 MHz), AV 400 (400.134 MHz) and DRX 500 (500.130 MHz) spectrometers (Bruker, Billerica, MA, USA). ^13^C NMR spectrum was recorded on a Bruker AV 300 (75.467 MHz) (see the Supplementary Materials in this article pp. S4–S9). All the NMR spectra were acquired for 5–10% solutions in CDCl_3_ or CD_3_OD at 300 K using the signal of the solvent as a standard. To confirm the structure of stable nitroxides, ^1^H NMR spectra were recorded of the solutions of corresponding hydroxylamines prepared via reduction of the nitroxide samples (10–20 mg) with Zn powder in a CD_3_OD–CF_3_COOH 10:1 mixture [22]. HRMS analyses were performed using a High-Resolution Mass Spectrometer DFS (Thermo Electron, Waltham, MA, USA).

Reactions were monitored by TLC on precoated TLC sheets ALUGRAM Xtra SIL G/UV_254_ ((Macherey-Nagel GmbH & Co. KG, Düren, Germany) using UV light 254 nm, 1% aqueous permanganate, 10% solution of phosphomolybdic acid in ethanol and Dragendorff reagent as visualizing agents. Kieselgel60 (Macherey-Nagel GmbH & Co. KG) was utilized as an adsorbent for column chromatography.

CCDC 2308349 (**3**), 2308350 (**4**), 2309021 (**6**), 2308351 (**8**), 2308352 (**10**), 2308353 (**12a**), 2308354 (**15a**), 2308355 (**15b**), 2308356 (**20**) contain the supplementary crystallographic data for this paper. These data can be obtained free of charge from The Cambridge Crystallographic Data Centre https://www.ccdc.cam.ac.uk/structures/ (accessed on 17 November 2023).

### 3.2. Synthesis

#### 3.2.1. A Reaction of Hydroxymethyl Nitroxides **1** and **8** with Methanesulfonyl Chloride

Method A: N,N-diisopropylethylamine (DIPEA) was added dropwise within 6 h to a stirred solution of **1** and methanesulfonyl chloride 1.4 g in dry chloroform, the amount and ratio of the reagents is shown in Table 2. The mixture was stirred overnight, washed with water and dried with MgSO_4_. The solution was concentrated in a vacuum and separated using column chromatography on silica gel, eluent chloroform; yields of **3** and **4** are shown in the Table 2.

*2,2,5,5-Tetraethyl-3,4-bis((methylsulfonyloxy)methyl)pyrrolidin-1-oxyl* (**3**), yellow crystals, m.p. 73–75 °C (hexane). IR (KBr) ν_max_: 1348, 1170 (OMs). ^1^H NMR (300 MHz; CD_3_OD, Zn/CF_3_COOH, δ): 1.06 (t, J_t_ = 7.4 Hz, 6H), 1.07 (t, J_t_ = 7.4 Hz, 6H), 1.88 (dq, J_d_ = 14.7 Hz, J_q_ = 7.4 Hz, 2H), 2.00 (q, J_q_ = 7.4 Hz, 4H), 2.06 (dq, J_d_ = 14.7 Hz, J_q_ = 7.4 Hz, 2H), 2.67–2.78 (m, 2H), 3.18 (s, 6H), 4.44–4.55 (m, 4H). Anal. Calcd for C_16_H_32_NO_7_S_2_: C, 46.36; H, 7.78; N, 3.38; S, 15.47; found: C, 46.49; H, 7.50; N, 3.57.

*2,2,5,5-Tetraethyl-3-(hydroxymethyl)-4-((methylsulfonyloxy)methyl)pyrrolidin-1-oxyl* (**4**), yellow crystals, m.p. 66–67 °C (hexane). IR (KBr) ν_max_: 3386 (OH), 1359, 1180 (OMs). ^1^H NMR (300 MHz; CD_3_OD, Zn/CF_3_COOH, δ): 1.04 (t, J_t_ = 7.4 Hz, 6H), 1.05 (t, J_t_ = 7.4 Hz, 3H), 1.06 (t, J_t_ = 7.4 Hz, 3H), 1.77–2.12 (m, 8H), 2.32 (ddd, J_d1_ = 11.8 Hz, J_d2_ = 5.6, J_d3_ = 4.9 Hz, 1H), 2.68 (ddd, J_d1_ = 11.8 Hz, J_d2_ = 7.3 Hz, J_d3_ = 3.6 Hz, 1H), 3.14 (s, 3H), 3.78 (dd, J_d1_ = 11.7 Hz, J_d2_ = 4.9 Hz, 1H), 3.83 (dd, J_d1_ = 11.7 Hz, J_d2_ = 5.6 Hz, 1H), 4.43 (dd. J_d1_ = 11.0 Hz, J_d2_ = 7.3 Hz, 1H), 4.54 (dd, J_d1_ = 11.0 Hz, J_d2_ = 3.6 Hz, 1H). ^13^C{^1^H} (75 MHz; CD_3_OD, Zn/CF_3_COOH, δ): 6.71, 6.77, 23.5, 23.7, 28.8, 28.9, 35.3, 48.1, 49.9, 58.42, 66.5, 69.7, 70.1. Anal. Calcd for C_15_H_30_NO_5_S: C, 53.54; H, 8.99; N, 4.16; S, 9.53; found: C, 53.38; H, 8.96; N, 4.20; S, 9.50.

Method B: Methanesulfonyl chloride (227 mg, 1.98 mmol) was added dropwise to a stirred solution of **8** (274 mg, 0.57 mmol) and DIPEA (335 mg, 2.59 mmol)) in dry chloroform (10 mL). The mixture was stirred at ambient temperature for 3 days (TLC control on silica gel, chloroform—methanol 100:1, visualization with UV-254), washed with water and dried with Na_2_CO_3_. The solution was concentrated in vacuum. The residue was purified using column chromatography on silica gel, eluent chloroform—methanol 100:1 to give **9** (247mg, 74%).

*3-(((tert-Butoxycarbonyl)amino)methyl)-2,2,5,5-tetraethyl-4-(((methylsulfonyl)oxy)methyl)pyrrolidin-1-oxyl* (**9**), yellow crystals, m.p. 135–136 °C (hexane—chloroform 25:1). IR (KBr) ν_max_: 3363, 1513 (NH), 1675 (C=O), 1358, 1178 (OMs). UV (EtOH) λ_max_ (log ε): 239 (3.34). Anal. Calcd for C_20_H_39_N_2_O_6_S: C, 55.15; H, 9.02; N, 6.43; S, 7.36; found: C, 54.89; H, 9.06; N, 6.38; S, 7.19. HRMS (EI/DFS) *m/z*: [M]^+^ calcd for C_20_H_39_N_2_O_6_S 435.2523, found 435.2525.

#### 3.2.2. Reaction of Mesylates with Sodium Azide (General Method)

A mixture of a mesylate **3**, **4**, or **9** (12 mmol), NaN_3_ (5 g, 77 mmol) and DMSO (50 mL) was heated to 60 °C and stirred for 4–6 h. The conversion was controlled using TLC (silica gel, chloroform, UV 254). The mixture was diluted with H_2_O (200 mL) and extracted with hexane-diethyl ether mixture 1:1 (3 × 100 mL). The extract was washed with water and dried with MgSO_4_. The solvent was distilled off and residue was purified as described below.

*3-(Azidomethyl)-2,2,5,5-tetraethyl-4-(hydroxymethyl)pyrrolidin-1-oxyl* (**5**). Crude nitroxide was dissolved in the mixture hexane–toluene 20:1 and the solution was cooled to −15 °C. The crystalline precipitate of **5** was filtered off and washed with cold hexane, yield 90%, m.p. 35–38 °C. IR (KBr) ν_max_: 3406 (OH), 2098 (N_3_). UV (EtOH) λ_max_ (log ε): 223 (3.35). Anal. Calcd for C_14_H_27_N_4_O_2_: C, 59.34; H, 9.60; N, 19.77; found: C, 59.34; H, 9.86; N, 19.81.

*3-(Azidomethyl)-4-((tert-butoxycarbonylamino)methyl)-2,2,5,5-tetraethylpyrrolidin-1-oxyl* (**10**). Yield 98%, yellow oil. IR (KBr) ν_max_: 3353 (NH), 2100, 2090 (N_3_), 1708 (C=O). UV (EtOH) λ_max_ (log ε): 223 (3.43). HRMS (EI/DFS) *m/z*: [M]^+^ calcd for C_19_H_36_O_3_N_5_ 382.2813, found 382.2817.

*3,4-Bis(azidomethyl)-2,2,5,5-tetraethylpyrrolidin-1-oxyl* (**12a**). Yield 99%, m.p. 62–65 °C (hexane). IR (KBr) ν_max_: 2193, 2096 (N_3_). Anal. Calcd for C_14_H_26_N_7_O: C, 54.52; H, 8.50; N, 31.79; found: C, 54.60; H, 8.46; N, 31.50.

*3,4-Bis(azidomethyl)-2,2,5,5-tetraethyl-2,5-dihydro-1H-pyrrol-1-oxyl* (**12b**). Crude nitroxide was purified using column chromatography on silica gel, hexane—ethyl acetate mixture 4:1 to give **12**, yield 93%, yellow crystals, m.p. 44–46 °C (hexane). IR (KBr) ν_max_: 2098 (N_3_). Anal. Calcd for C_14_H_24_N_7_O: C, 54.88; H, 7.90; N, 32.00; found: C, 54.70; H 8.00; N, 32.12.

*3-(Aminomethyl)-2,2,5,5-tetraethyl-4-(hydroxymethyl)pyrrolidin-1-oxyl* (**6**). A solution of nitroxide azide **5** (243 mg, 0.86 mmol) in absolute ethyl ether (5 mL) was added dropwise to a stirred solution of lithium aluminum hydride (211 mg, 5.55 mmol) in absolute ethyl ether (20 mL). The conversion was controlled using TLC (silica gel, hexane—ether 1:1, UV 254). The flask was cooled in an ice bath and carefully quenched with 10 mL of 2.5 M aqueous sodium hydroxide. The organic layer was separated via decantation, the wet precipitate was washed with diethyl ether (10 mL × 3 times) and the combined extract was evaporated under reduced pressure. The residue was purified by column chromatography on silica gel (eluent chloroform—methanol—aqueous ammonia 190:10:1). Yield of **6** 187 mg (82%), m.p. 43–44 °C (dec). IR (KBr) ν_max_: 3463, 3380, 3359, 3288, 3193 (OH, NH_2_). UV (EtOH) λ_max_ (log ε): 238 (3.29). ^1^H NMR (300 MHz; CD_3_OD, Zn/CF_3_COOH, δ): 0.91 (t, J_t_ = 7.4 Hz, 3H), 0.96 (t, J_t_ = 7.4 Hz, 3H), 0.97 (t, J_t_ = 7.4 Hz, 3H), 0.98 (t, J_t_ = 7.4 Hz, 3H), 1.16–2.03 (m, 8H), 2.32 (dd, J_d1_ = 11 Hz, J_d2_ = 10 Hz, 1H), 2.46 (dd, J_d1_ = 11 Hz, J_d2_ = 10 Hz, 1H), 2.90 (dd, J_d1_ = 12 Hz, J_d2_ = 11 Hz, 1H), 3.16 (d, J_d_ = 12 Hz, 1H), 3.52 (dd, J_d1_ = 11 Hz, J_d2_ = 10 Hz, 1H), 3.87 (d, J_d1_ = 10 Hz, 1H). Anal. Calcd for C_14_H_29_N_2_O_2_: C, 65.33; H, 11.36; N, 10.88; found: C, 65.11; H 11.38; N, 10.67. HRMS (EI/DFS) *m/z*: [M]^+^ calcd for C_14_H_29_O_2_N_2_ 257.2224, found 257.2225.

*3-(((tert-Butoxycarbonyl)amino)methyl)-2,2,5,5-tetraethyl-4-(hydroxymethyl)pyrrolidin-1-oxyl* (**8**). A solution of Boc_2_O (505 mg, 2.31 mmol) in dichloromethane (5 mL) was cooled in an ice bath and the solution of nitroxide **6** (520 mg, 2.02 mmol), triethylamine (0.83 mL, 5.95 mmol) in dichloromethane (1 mL) was added dropwise. The reaction mixture was allowed to stay under stirring at the ambient temperature for 24 h. The conversion was controlled using TLC (silica gel, chloroform—methanol—aqueous ammonia 190:10:1, UV 254). The mixture was diluted with 10% aqueous solution of ammonium chloride (10 mL) and extracted with ethyl acetate (3 × 10 mL). The extract was washed with brine and dried with Na_2_SO_4_. The solvent was distilled off and residue was purified by column chromatography on silica gel (eluent chloroform—methanol 200:1 gradient enhanced to 50:1). Yield 776 mg (99%), yellow crystals, m.p. 82–85 °C. IR (KBr) ν_max_: 3376, 3320 (OH, NH), 1720, 1691 (C=O), 1540 (NH). UV (EtOH) λ_max_ (log ε): 238 (3.37). Anal. Calcd for C_19_H_38_N_2_O_4_: C, 63.83; H, 10.43; N, 7.84; found: C, 63.58; H 10.53; N, 7.94. HRMS (EI/DFS) *m/z*: [M]^+^ calcd forC_19_H_38_O_4_N_2_ 357.2748, found 357.2753.

#### 3.2.3. The Eschweiler–Clarke Methylation of Nitroxides **10** and **13a,b** (General Method)

The mixture of nitroxide **10**, **13a** or **13b** (0.06 mmol), 21% aqueous solution of formaldehyde (0.16 mL, 1.05 mmol) and formic acid (95%) (525 mg, 10.84 mmol) was heated in an oil bath at 60 °C for 36 h. The conversion was controlled using TLC (silica gel, chloroform—methanol—aqueous ammonia 100:10:1, UV 254). The reaction mixture was diluted with water, basified (Na_2_CO_3_) to pH ~ 8, and extracted with ether (3 × 10 mL). An organic extract was dried over Na_2_CO_3_, and the solvent was removed under reduced pressure.

*3-(Azidomethyl)-4-((dimethylamino)methyl)-2,2,5,5-tetraethylpyrrolidin-1-oxyl* (**11a**). Yield 81% (from **10**) and 80% (from **13a**), yellow crystals, m.p. 45–46 °C. IR (KBr) ν_max_: 2821, 2769 (N-CH_3_), 2098 (N_3_). UV (EtOH) λ_max_ (log ε): 236 (3.43). Anal. Calcd for C_16_H_32_N_5_O: C, 61.90; H, 10.39; N, 22.56; found: C, 61.74; H, 10.43; N, 22.59. HRMS (EI/DFS) *m/z*: [M]^+^ calcd for C_16_H_32_ON_5_ 310.2601, found 310.2599.

*3-(Azidomethyl)-4-((dimethylamino)methyl)-2,2,5,5-tetraethyl-2,5-dihydro-1H-pyrrol-1-oxyl* (**11b**). Yield 56%, yellow oil. IR (neat) ν_max_: 2817, 2769 (N-CH_3_), 2094 (N_3_). Anal. Calcd for C_16_H_30_N_5_O: C, 62.30; H, 9.80; N, 22.71; found: C, 62.17; H, 9.78; N, 22.57. HRMS (EI/DFS) *m/z*: [M]^+^ calcd for C_16_H_30_ON_5_ 308.2445, found 308.2443.

#### 3.2.4. General Procedure for Quaternization of Dimethylamino Group

Iodomethane (120 mg, 0.85 mmol) was added to the solution of nitroxide **11a,b** or **15a,b** (0.1 mmol) in dry diethyl ether (0.5 mL) and the resulting mixture was allowed to stay at ambient temperature for 72 h. The precipitate formed was collected on a filter and washed with dry diethyl ether.

*3-((2,5-Dioxo-2,5-dihydro-1H-pyrrol-1-yl)methyl)-2,2,5,5-tetraethyl-4-((trimethylammonio)methyl)pyrrolidin-1-oxyl monoiodide* (**16a**). Yield 41%, yellow crystals, m.p. 155–156 °C (dec). IR (KBr) ν_max_: 1768, 1705 (C=O). Anal. Calcd for C_21_H_37_IN_3_O_3_: C, 49.80; H, 7.36; N, 8.30; found: C, 49.75; H, 7.60; N, 8.17.

*3-((2,5-Dioxo-2,5-dihydro-1H-pyrrol-1-yl)methyl)-2,2,5,5-tetraethyl-4-((trimethylammonio)methyl)-2,5-dihydro-1H-pyrrol-1-oxyl monoiodide* (**16b**). Yield 20%, yellow crystals, m.p. 204–205 °C. IR (KBr) ν_max_: 1772, 1712 (C=O). Anal. Calcd for C_21_H_35_IN_3_O_3_: C, 50.00; H, 6.99; N, 8.33; I, 25.16; found: C, 50.11; H, 7.14; N, 8.40; I, 25.30.

*3-(Azidomethyl)-2,2,5,5-tetraethyl-4-((trimethylammonio)methyl)pyrrolidin-1-oxyl monoiodide* (**17a**). Yield 42%, yellow crystals, m.p. 91–92 °C (dec). IR (KBr) ν_max_: 2100 (N_3_). UV (EtOH) λ_max_ (log ε): 219 (4.09). Anal. Calcd for C_17_H_35_N_5_OI: C, 45.13; H, 7.80; N, 15.48; I, 28.05; found: C, 44.99; H, 7.63; N, 15.67; I, 28.02.

*3-(Azidomethyl)-2,2,5,5-tetraethyl-4-((trimethylammonio)methyl)-2,5-dihydro-1H-pyrrol-1-oxyl monoiodide* (**17b**). Yield 85%, yellow crystals, m.p. 148–149 °C. IR (KBr) ν_max_: 2098 (N_3_). Anal. Calcd for C_17_H_33_N_5_OI: C, 45.34; H, 7.39; N, 15.55; I, 28.18; found: C, 45.48; H, 7.60; N, 15.75; I, 28.24.

#### 3.2.5. Reaction of Nitroxide **12a** with Dimethyl Acetylenedicarboxylate (DMAD)

A solution of DMAD (149 mg, 1.00 mmol) in toluene (2 mL) was added to the solution of nitroxide **12a** (305 mg, 1.00 mmol) in toluene (2 mL). The resulting mixture was stirred and heated in an oil bath at 70 °C for 24 h. The conversion was controlled using TLC (silica gel, chloroform—methanol 200:1, UV 254). The solvent was distilled off and residue was separated by column chromatography on silica gel (eluent hexane—ethyl acetate 1:1) to give **18** (285 mg, 64%) and **19** (129 mg, 22%).

*3-(Azidomethyl)-4-((4,5-bis(methoxycarbonyl)-1H-1,2,3-triazol-1-yl)methyl)-2,2,5,5-tetraethylpyrrolidin-1-oxyl* (**18**). Yellow crystals, m.p. 113–114 °C. IR (KBr) ν_max_: 2200, 2098 (N_3_), 1733 (C=O). Anal. Calcd for C_20_H_32_N_7_O_5_: C, 53.32; H, 7.16; N, 21.76; found: C, 53.47; H, 7.06; N, 21.94. HRMS (EI/DFS) *m/z*: [M]^+^ calcd for C_20_H_32_O_5_N_7_ 450.2459, found 450.2461.

*3,4-Bis((4,5-bis(methoxycarbonyl)-1H-1,2,3-triazol-1-yl)methyl)-2,2,5,5-tetraethylpyrrolidin-1-oxyl* (**19**). Yellow crystals, m.p. 178–179 °C (dec). IR (KBr) ν_max_: 1731 (C=O). Anal. Calcd for C_26_H_38_N_7_O_9_: C, 52.69; H, 6.46; N, 16.54; found: C, 52.87; H, 6.38; N, 16.26. HRMS (EI/DFS) *m/z*: [M]^+^ calcd for C_26_H_38_O_9_N_7_ 592.2726, found 592.2721.

#### 3.2.6. General Procedure of the Synthesis of Amines **13a,b** and **14a,b** with PPh_3_

Triphenylphosphine (1 eq. for **12a,b** or 1.1 eq. for **11a,b**) was added to the solution of azide **11a,b** or **13a,b** (1 eq., 3 mmol) in dry diethyl ether (10 mL). The reaction mixture was stirred and heated under reflux for 3 h. The solvent was evaporated under reduced pressure and 50% aqueous ethanol (10 mL) was added to the residue. The resulted mixture was heated under reflux with stirring for 10 h. The ethanol was distilled off under reduced pressure and the water layer was acidified with 1M aqueous NaHSO_4_ to pH = 2 and extracted with chloroform (3 × 20 mL). The water layer was basified with sodium carbonate to pH~9–10, extracted with chloroform (5 × 20 mL), the extract was dried with sodium carbonate, and the solvent was evaporated under reduced pressure.

*3-(Aminomethyl)-4-(azidomethyl)-2,2,5,5-tetraethylpyrrolidin-1-oxyl* (**13a**). Yield 64%, yellow crystals, m.p. 36–40 °C. IR (KBr) ν_max_: 3390, 3360 (NH), 2177, 2098 (N_3_). Anal. Calcd for C_14_H_28_IN_5_O: C, 59.54; H, 9.99; N, 24.80; found: C, 59.53; H, 9.94; N, 24.57. HRMS (EI/DFS) *m/z*: [M]^+^ calcd for C_14_H_28_ON_5_ 282.2288, found 282.2291.

*3-(Aminomethyl)-4-(azidomethyl)-2,2,5,5-tetraethyl-2,5-dihydro-1H-pyrrol-1-oxyl* (**13b**). Yield 73%, yellow oil. IR (neat) ν_max_: 3380, 3311 (NH), 2096 (N_3_). HRMS (EI/DFS) *m/z*: [M]^+^ calcd for C_14_H_26_ON_5_ 280.2132, found 280.2131.

*3-(Aminomethyl)-4-((dimethylamino)methyl)-2,2,5,5-tetraethylpyrrolidin-1-oxyl* (**14a**). Yield 90%, yellow oil. IR (neat) ν_max_: 3361, 3270 (NH), 2821, 2767 (N-CH_3_). NMR sample was prepared as follows: after the procedure [21] a solution was evaporated, the residue was mixed with 10% NaOH in MeOH (1 mL) and again evaporated, the residue was triturated with CDCl_3_ and the solution was filtered into the NMR tube. ^1^H NMR (300 MHz; CDCl_3_, δ): 0.80 (t, J_t_ = 7.3 Hz, 3H), 0.81 (t, J_t_ = 7.4 Hz, 6H), 0.82 (t, J_t_ = 7.3 Hz, 3H), 1.14–1.32 (m, 7H), 1.32–1.51 (m, 3H), 1.76 (ddd, J_d1_ = 11.2 Hz, J_d2_ = 9.2 Hz, J_d3_ = 2.6 Hz, 1H), 1.92 (ddd, J_d1_ = 11.2 Hz, J_d2_ = 10.6 Hz, J_d3_ = 1.8 Hz, 1H), 2.00 (dd, J_d1_ = 12.5 Hz, J_d2_ = 1.8 Hz, 1H), 2.17 (s, 6H), 2.35 (dd, J_d1_ = 12.5 Hz, J_d2_ = 10.6 Hz, 1H), 2.53 (dd, J_d1_ = 12.8 Hz, J_d2_ = 9.2 Hz, 1H), 2.71 (dd, J_d1_ = 12.8 Hz, J_d2_ = 2.6 Hz, 1H). ^13^C{^1^H} NMR (75 MHz; CDCl_3_, δ): 7.4, 7.5, 8.2, 8.6, 28.2, 28.4, 29.2, 29.6, 30.8, 42.1, 45.5, 46.4, 54.3, 60.4, 62.5, 62.6. Anal. Calcd for C_16_H_34_N_3_O: C, 67.56; H, 12.05; N, 14.77; found: C, 67.72; H, 11.77; N, 14.60. HRMS (EI/DFS) *m/z*: [M]^+^ calcd for C_16_H_34_ON_3_ 284.2696, found 284.2698.

*3-(Aminomethyl)-4-((dimethylamino)methyl)-2,2,5,5-tetraethyl-2,5-dihydro-1H-pyrrol-1-oxyl* (**14b**). Yield 60%, yellow oil. IR (neat) ν_max_: 3376 (NH), 2815, 2765 (N-CH_3_). ^1^H NMR (300 MHz; CD_3_OD, Zn/CF_3_COOH, δ): 1.06 (t, J_t_ = 7.4 Hz, 6H), 1.07 (t, J_t_ = 7.4 Hz, 6H), 1.94 (dq, J_d_ = 14.7 Hz, J_q_ = 7.4 Hz, 2H), 1.97 (dq, J_d_ = 14.7 Hz, J_q_ = 7.4 Hz, 2H), 2.15 (dq, J_d_ = 14.7 Hz, J_q_ = 7.4 Hz, 2H), 2.17 (dq, J_d_ = 14.7 Hz, J_q_ = 7.4 Hz, 2H), 2.71–2.94 (m, 6H), 3.71–3.99 (m, 4H). HRMS (EI/DFS) *m/z*: [M]^+^ calcd for C_16_H_32_ON_3_ 282.2540, found 282.2539.

#### 3.2.7. General Procedure of the Synthesis of Maleimides **7** and **15a,b**

A solution of N-methoxycarbonyl maleimide NMCM (51 mg, 0.33 mmol) in absolute THF (10 mL) was cooled in an ice bath and the solution of nitroxide **6** or **14a,b** (0.30 mmol) in absolute THF (5 mL) was added dropwise. The reaction mixture was allowed to stay under stirring for 5 min. The conversion was controlled using TLC (silica gel, methanol—ethyl acetate 1:9, UV 254). The mixture was diluted with saturated aqueous solution of NaHCO_3_ (10 mL) and allowed to stay under stirring for 1 h. The conversion was controlled using TLC (silica gel, hexane—ethyl acetate 1:1, UV 254). The mixture was diluted with water and extracted with ethyl acetate (3 × 10 mL). The extract was washed with brine and dried with Na_2_SO_4_. The solvent was distilled off and residue was purified by column chromatography on silica gel (eluent hexane—ethyl acetate 1:1).

*3-((2,5-Dioxo-2,5-dihydro-1H-pyrrol-1-yl)methyl)-2,2,5,5-tetraethyl-4-(hydroxymethyl)pyrrolidin-1-oxyl* (**7**). Yield 29%, yellow crystals, m.p. 44–55 °C. IR (KBr) ν_max_: 3546, 3419 (OH), 1768, 1706 (C=O). UV (EtOH) λ_max_ (log ε): 219 (4.03). Anal. Calcd for C_18_H_29_N_2_O_4_: C, 64.07; H, 8.66; N, 8.30; found: C, 64.17; H, 8.59; N, 8.31.

*3-((Dimethylamino)methyl)-4-((2,5-dioxo-2,5-dihydro-1H-pyrrol-1-yl)methyl)-2,2,5,5-tetraethylpyrrolidin-1-oxyl* (**15a**). Yield 72%, yellow crystals, m.p. 90–91 °C (dec). IR (KBr) ν_max_: 2861, 2821 (N-CH_3_), 1768, 1706 (C=O). Anal. Calcd for C_20_H_34_N_3_O_3_: C, 65.90; H, 9.40; N, 11.53; found: C, 65.78; H 2.23; N, 11.39. HRMS (EI/DFS) *m/z*: [M]^+^ calcd for C_20_H_34_O_3_N_3_ 364.2595, found 364.2597.

*3-((Dimethylamino)methyl)-4-((2,5-dioxo-2,5-dihydro-1H-pyrrol-1-yl)methyl)-2,2,5,5-tetraethyl-2,5-dihydro-1H-pyrrol-1-oxyl* (**15b**). Yield 73%, yellow crystals, m.p. 96–101 °C. IR (KBr) ν_max_: 2813, 2763 (N-CH_3_), 1766, 1706 (C=O). UV (EtOH) λ_max_ (log ε): 202 (4.24), 217 (4.16). Anal. Calcd for C_20_H_32_N_3_O_3_: C, 66.27; H, 8.90; N, 11.59; found: C, 66.08; H 8.79; N, 11.38. HRMS (EI/DFS) *m/z*: [M]^+^ calcd for C_20_H_32_O_3_N_3_ 362.2438, found 362.2435.

*3-(Azidomethyl)-4-((4,5-dicarboxy-1H-1,2,3-triazol-1-yl)methyl)-2,2,5,5-tetraethylpyrrolidin-1-oxyl monosodium salt* (**20**). A solution of 0.5 M aqueous NaOH (10 mL) was added to the solution of nitroxide **18** (197 mg, 0.44 mmol) in methanol (10 mL). The reaction mixture was allowed to stay at ambient temperature for 72 h. The conversion was controlled using TLC (silica gel, chloroform—methanol 200:1, UV 254). The mixture was diluted with 0.5 M aqueous solution of NaH_2_PO_4_ (20 mL) and evaporated. The residue was triturated with a mixture of chloroform—methanol (60:1), filtered and again evaporated (this procedure was repeated several times). RSA sample was prepared by slow evaporation of the solvent (chloroform—methanol 5:1) in the cold place. Yield 72%, yellow crystals, m.p. 106–107 °C (dec). IR (KBr) ν_max_: 2102 (N_3_), 1724 (C=O), 1631 (COO^−^). Anal. Calcd for C_18_H_27_N_7_NaO_5_: C, 48.64; H, 6.12; N, 22.06; found: C, 48.89; H 5.95; N, 21.91.

*3,4-Bis(carboxymethyl)-2,2,5,5-tetraethylpyrrolidin-1-oxyl* (**23**). A solution of **24** (1.5 g, 5.42 mmol) in ethanol (20 mL) was heated to boiling upon stirring and a solution of sodium hydroxide (5 g, 125 mmol) in water (50 mL) was added portionwise within 4 h. The ethanol/water mixtures were allowed to distill off upon stirring until the volume of the solution reached 40 mL and the mixture was stirred under reflux for 48 h. The solution was allowed to cool down to ambient temperature and extracted with diethyl ether. The aqueous phase was separated, acidified to pH = 2 and extracted with ethyl acetate (5 × 30 mL). The combined extracts were dried with MgSO_4_. The solvent was distilled off in vacuum to give **23** as a light-yellow powder, yield 1.5 g (88%), m.p. 193–194 °C (ethyl acetate). IR (KBr) ν_max_: 1706 (C=O). ^1^H NMR (300 MHz; CD_3_OD, Zn/CF_3_COOH, δ): 1.00 (t, J_t_ = 7.4 Hz, 6H), 1.05 (t, J_t_ = 7.4 Hz, 6H), 1.75 (dq, J_d_ = 15.1 Hz, J_q_ = 7.4 Hz, 2H), 1.87 (dq, J_d_ = 14.2 Hz, J_q_ = 7.4 Hz, 2H), 1.92 (dq, J_d_ = 14.2 Hz, J_q_ = 7.4 Hz, 2H), 1.96 (dq, J_d_ = 15.1 Hz, J_q_ = 7.4 Hz, 2H), 2.43–2.70 (m, 6H); ^13^C{^1^H} NMR (75 MHz; CD_3_OD, Zn/CF_3_COOH, δ): 8.4, 8.6, 25.9, 30.4, 34.1, 49.1, 71.9, 175.3. Anal. Calcd for C_16_H_28_NO_5_: C, 61.12; H, 8.98; N, 4.46; found: C, 61.17; H 8.62; N, 4.29.

*3,4-Bis(cyanomethyl)-2,2,5,5-tetraethylpyrrolidin-1-oxyl* (**24**). A mixture of **3** (2.260 g, 5.45 mmol), NaCN (1.5 g, 30.6 mmol) and DMSO (25 mL) was stirred at 60 °C for 18 h. The mixture was diluted with brine (50 mL), extracted with ethyl acetate (3 × 20 mL) and the combined extracts were washed with water and dried with MgSO_4_. The solvent was distilled off in vacuum to give **24** as a yellow crystals, yield 1.5 g (99%), m.p. 94–98 °C (hexane). IR (KBr) ν_max_: 2244 (C≡N). ^1^H NMR (400 MHz; CD_3_OD, Zn/CF_3_COOH, δ): 1.07 (t, J_t_ = 7.4 Hz, 6H), 1.10 (t, J_t_ = 7.4 Hz, 6H), 1.90 (dq, J_d_ = 14.7 Hz, J_q_ = 7.4, 2H), 1.99 (dq, J_d_ = 14.2 Hz, J_q_ = 7.4 Hz, 2H), 2.03 (dq, J_d_ = 14.2 Hz, J_q_ = 7.4 Hz, 2H), 2.06 (dq, J_d_ = 14.7 Hz, J_q_ = 7.4 Hz, 2H), 2.53–2.62 (m, 2H), 2.88–2.96 (m, 4H); ^13^C{^1^H} NMR (100 MHz; CD_3_OD, Zn/CF_3_COOH, δ): 7.1, 7.2, 15.5, 23.9, 28.7, 47.1, 70.1, 117.7. HRMS (EI/DFS) *m/z*: [M]^+^ calcd for C_16_H_26_ON_3_ 276.2070, found 276.2068.

### 3.3. EPR Measurements and Kinetics

The stock solutions of the nitroxides in DMSO (20 mM) were diluted 100-fold with 5 mM phosphate-citrate-borate buffer pH 7.4. EPR spectra were recorded on Elexsys E540 X-band spectrometer (Bruker Corporation, Billerica, MA, USA) in a 50 µL glass capillary for 0.2 mM solutions, with the following spectrometer settings: frequency, 9.87 GHz; centerfield, 350.6 mT; sweep range, 10 mT; microwave power, 2.0 mW; modulation amplitude, 0.05 mT; time constant, 10.24 ms; and conversion time, 20.48 ms. Simulations of solution electron spin resonance lines were carried out in the EasySpin software (5.2.35), which is available at http://www.easypin.org (accessed on 18 May 2023). The spectra with resolved hyperfine structure were obtained for degassed solutions using the following settings: microwave power, 2.0 mW; modulation amplitude, 0.005 and 0.0025 mT; modulation frequency 100 KHz.

The pH-dependences of the EPR spectra were investigated using the 0.2 mM solutions of the nitroxides in 5 mM phosphate-citrate-borate buffer on a Bruker ER-200D-SRC spectrometer in 50 µL glass capillary for 0.2 mM radical solutions. Spectrometer settings: frequency, 9.87 GHz; microwave power, 5.0 mW; modulation amplitude, 0.1 mT; time constant, 50–100 ms; and conversion time, 50.12 ms. Small portions of 0.1–1 N HCl or 0.1–1 N NaOH were added to the stirred solution of a nitroxide in buffer at 25 °C, while the pH was controlled using pH-meter. After the equilibrium was established, the samples were taken from the solution and the EPR spectra were measured. The experimental points were fitted with the Equation (1) [43] to obtain the pKa values.

(1) $$a_{N}(pH)=\frac{\left(p_{1}+p_{2}\times 10^{{\mathrm{pK}}_{1}-\mathrm{pH}}+p_{3}\times 10^{{\mathrm{pK}}_{1}+{\mathrm{pK}}_{2}-2\times \mathrm{pH}}\right)}{1+10^{{\mathrm{pK}}_{1}-\mathrm{pH}}+10^{{\mathrm{pK}}_{1}+{\mathrm{pK}}_{2}-2\times \mathrm{pH}}}$$

For kinetic measurements in water, stock solutions of nitroxide, ascorbic acid and glutathione in phosphate-citrate-borate buffer (5 mM, pH 7.4) were prepared, and pH was adjusted to 7.4 with NaOH or HCl. All the solutions were deoxygenated with argon, carefully and quickly mixed in a small tube to attain final concentrations (nitroxide, 0.2–04 mM; GSH, 5 mM; and ascorbate, 100–300 mM) and were placed into an EPR capillary (50 μL). The choice of acsorbate concentration is a compromise between the low reduction rate, which requires very long experiment time and deviations of the kinetics from linearity at higher ascorbate concentrations due to ionic strength effects. Since the ascorbate oxidation is slow under these conditions, the 5 mM concentration of GSH was sufficient to suppress the reverse reaction of ascorbate radical with the hydroxylamine, because the rate of the ascorbate radical formation was low. The increase in GSH concentration produced little effect on the final reduction rate constant values. The capillary was sealed on both sides and placed into the EPR resonator. The decay of amplitude of the low-field component of the EPR spectrum was followed to obtain the kinetics. The initial part of the decay curves (up to 20 min) was used for fitting. Kinetics of the decay was fitted to a monoexponential function to calculate the first-order rate constants. Then, these constants were divided by the concentration of ascorbic acid to calculate the second-order reaction constants. Partition coefficients in a water–octanol mixture were estimated from the amplitudes of the EPR spectra of a nitroxide in water after extensive shaking with different portions of added octanol and separation using centrifugation.

The measurements of kinetics of nitroxide decay in liver homogenates were performed using Bruker ER-200D-SRC spectrometer in a 50 µL glass capillary for 0.2 mM solutions, with the following spectrometer settings: frequency, 9.87 GHz; centerfield, 350.6 mT; sweep range, 10 mT; microwave power, 2.0 mW; modulation amplitude, 0.1 mT; time constant, 10.24 ms; and conversion time, 20.48 ms. Before measurement, 10 mM solutions of the nitroxides in PBS were prepared ex tempore and mixed with thawed homogenates (final concentration 0.2 mM). Homogenates were then loaded into 50 μL glass capillaries, which were then sealed with plasticine and inserted into the chamber of the EPR spectrometer. The time elapsed between thawing and beginning of the recording was 7 min. The decay of low-field component of the EPR spectrum was followed to obtain the kinetics.

### 3.4. Animals

Male Wistar rats weighing 220–250 g were kept under standard conditions with unlimited access to water and pelleted food. All manipulations with animals were carried out in a strict accordance with the legislation of the Russian Federation, GOST 33044-2014 “Principles of Good Laboratory Practice”, GOST 33647-2015 “Principles of Good Laboratory Practice (GLP). Terms and definitions”, Decision “On approval of the Rules of Good Laboratory Practice of the Eurasian Economic Union in the field of circulation of medicines” dated 3 November 2016 No. 81 and the provisions of Directive 2010/63/EU of the EU Parliament and the Council of the European Union dated 22 September 2010 on the protection of animals used for scientific purposes. The animal study protocol was approved by the Ethics Committee of the Institute of Organic Chemistry N.N. Vorozhtsov SB RAS (protocol No. P-05-06.2022-14, approved 5 June 2022).

Animals were sacrificed by decapitation, organ tissue (liver) was excised on ice, rinsed with ice-cold 10 mM PBS (pH 7.4), sheared with scissors and homogenized in PBS (1/1 by weight) on ice using electric homogenizer fitted with a Teflon head. The homogenates were snap-frozen in liquid nitrogen.

## 4. Conclusions

In this work, we tested various methods of synthesis heterofunctional derivatives of the sterically shielded nitroxide **1**. The resulting maleimide and azide spin labels of both pyrrolidine and pyrroline series demonstrated high resistance to reduction. Besides that, they contain hydrophilic moieties, making them soluble in water and allowing for additional coordination with target biomolecules for more precise distance measurements. The new pyrroline spin labels are especially valuable due to simple EPR spectra with relatively narrow lines. 

The decay of the new nitroxides in rat liver homogenates was studied. The results revealed higher stability of negatively charged and neutral nitroxides as compared to the positively charged ones. This information will be used in design of the spin probes for in vivo EPRI and MRI applications.

Another important finding in this work is the unexpectedly high sensitivity of the EPR spectra of **14a** to pH changes. It is not only the case that the nitrogen *hfc* is decreasing by 0.055 mT upon second protonation (*pK_a_* 3.0), but also hydrogen *hfc* constants are increasing by 0.075 mT. Unless these effects are somewhat lower than pH-sensitivity of widely used spin probes [4] and pK is far from physiologically important range, this is the first example of a functional spin probe where variation of *hfc* on γ-hydrogens due to conformational changes is used. We believe that similar structures can be designed where comparable changes can result, for example, from a reaction with thiols (see [46]). High stability to reduction makes further investigation of these nitroxides very promising.

## Figures and Tables

**Table 1:** Parameters of the EPR spectra of nitroxides in 50 mM phosphate buffer (pH 7.4) with the addition of 1% DMSO at concentration of 0.2 mM (*a*_N_–*hfc* constant on nitrogen_,_ *a*_H_–*hfc* constant on methylene hydrogens of ethyl groups, H_p-p_–peak to peak linewidth), partition coefficients (K_p_) in octanol/aqueous 5 mM phosphate-citrate-borate buffer pH 7.4 mixtures, and rates of reduction with ascorbate (k_red_) (100–300 mM) in 5 mM phosphate-citrate-borate buffer, pH 7.4, 25 °C in the presence of glutathione 5 mM. For spectrometer settings see Section 3.

Nitroxide	*a*_N_, mT ±0.005	*a*_H_, mT ±0.005	H_p-p_, mT ±0.005	g-Factor ±0.00002	K_p_	k_red_ × 10^4^, M^−1^ s^−1^
**1**	1.57	0.22; 0.22	0.125	2.00563 [27]	41 ± 2	3.3 ± 0.8 [26]
**5**	1.52	0.22; 0.24	0.27	2.00562		
**6**	1.51	0.22; 0.23	0.26	2.00562		
**7**	1.52	0.24; 0.26	0.28	2.00562	84 ± 2	
**8**	1.52	0.24; 0.25	0.27	2.00562		6.0 ± 2.0
**9**	1.51	0.23; 0.27	0.28	2.00562		3.0 ± 1.0
**10**	1.50	0.23; 0.25	0.27	2.00562		3.0 ± 1.0
**11a**	1.49	0.23; 0.27	0.26	2.00563	52 ± 5	4.0 ± 1.0
**11b**	1.51		0.21	2.00562	15 ± 1	15 ± 4.0
**13a**	1.49	0.22; 0.28	0.26	2.00563	350 ± 35	6.3 ± 0.9
pH = 1.5 *	1.51					
pH = 13 *	1.53					
**13b**	1.51		0.18	2.00562	45 ± 2	7.6 ± 0.3
pH = 1.5 *	1.53					
pH = 13 *	1.55					
**14a**	1.51	0.20; 0.28	0.26	2.00562		6.4 ± 1.0
pH = 7.4 *	1.52	0.23; 0.23				
pH = 1.5 *	1.46	0.31; 0.31				
pH = 13 *	1.54	0.25; 0.25				
**14b**	1.52		0.19	2.00563	7 ± 0.3	8 ± 1.0
pH = 7.4 *	1.53					
pH = 1.5 *	1.49					
pH = 13 *	1.55					
**15a**	1.48	0.26; 0.31		2.00562		
**15b**	1.51		0.23	2.00563		
**16a**	1.47	0.23; 0.36	0.29	2.00562	0	
**16b**	1.48		0.25	2.00562		
**17a**	1.48	0.22; 0.27	0.26	2.00561	0	3.0 ± 1.0
**17b**	1.49		0.23	2.00562	0	16 ± 3.0
**20**	1.50	0.24; 0.31	0.28	2.00561	0	5.9 ± 1.2
**21**	1.55	0.19	0.26	2.00563	270	12.5 ± 0.3 [22]
**22**	1.54		0.16	2.00563	37 ± 2	3.4 ± 0.1
**23**	1.53	0.24; 0.29	0.27	2.00562		

* in 5 mM phosphate-citrate-borate buffer, for details see Section 3.

**Table 2:** Reaction conditions, conversion and yields of **3** and **4**.

No.	1, g/mmol	MsCl, g/eq.	DIPEA, g/eq.	CHCl_3_, mL	Conversion of 1, %	Yield	
						3, %	4, %
1	2/7.75	1.4/1.58	1.4/1.4	50	85	12	68
2	4/15.5	3/1.68	3/1.5	50	>95	35	55
3	4/15.5	5/2.8	4.8/2.2	50	100	>95	0

## Data Availability

All the data are in the text and the Supplementary Information in this article.

## Supplementary Materials

The following supporting information can be downloaded at: https://www.mdpi.com/article/10.3390/ijms25031550/s1, NMR spectra of **3**, **4**, **6**, **14a**, **14b**, **23** and **24**; IR spectra of all new compounds, X-ray diffraction data for **3**, **4**, **6**, **8**, **10**, **12a**, **15a**, **15b**, and **20**, EPR spectra of **5**–**10**, **11a, 11b, 13a,b**–**17a,b** and **20**–**23**.
