# Supplementary material for: Hydrophilic Reduction-Resistant Spin Labels of Pyrrolidine and Pyrroline Series from 3,4-Bis-hydroxymethyl-2,2,5,5-tetraethylpyrrolidine-1-oxyl
Source: Int J Mol Sci. 2024 Jan 26;25(3):1550. doi: 10.3390/ijms25031550 (PMC10855552; doi:10.3390/ijms25031550)
Supplement: Supplementary file 1 [file ijms-25-01550-s001.zip › ijms-2771046-SI.pdf]

Supporting Information

Hydrophilic Reduction-Resistant Spin Labels of Pyrrolidine and Pyrroline Series from 3,4-Bis-hydroxymethyl-2,2,5,5-tetraethylpyrrolidine-1-oxyl

Mikhail S. Usatov [1,2], Sergey A. Dobrynin [1], Yuliya F. Polienko [1], Denis A. Morozov [1], Yurii I. Glazachev [3],
Sergey V. An'kov [1], Tatiana G. Tolstikova [1], Yuri V. Gatilov [1], Irina Yu. Bagryanskaya [1], Arthur E. Raizvikh [1,4],
Elena G. Bagryanskaya [1] and Igor A. Kirilyuk [1,*]

[1] N. N. Vorozhtsov Novosibirsk Institute of Organic Chemistry SB RAS, Lavrentiev Ave. 9, Novosibirsk 630090, Russia; musatov@nioch.nsc.ru (M.S.U.); dobrynin@nioch.nsc.ru (S.A.D.); polienko@nioch.nsc.ru (Y.F.P.); dmorozov@nioch.nsc.ru (D.A.M.); sergey.ankov42@gmail.com (S.V.A.); tolstiktg@nioch.nsc.ru (T.G.T.); gatilov@nioch.nsc.ru (Y.V.G.); bagryan@nioch.nsc.ru (I.Y.B.); arturaiz@nioch.nsc.ru (A.E.R.); egbagryanskaya@nioch.nsc.ru (E.G.B.)
[2] Department of Natural Sciences, Novosibirsk State University, Pirogova Str. 1, Novosibirsk 630090, Russia
[3] Voevodsky Institute of Chemical Kinetics and Combustion SB RAS, Institutskaya 3, Novosibirsk 630090, Russia; glaza@kinetics.nsc.ru
[4] Department of Physics, Novosibirsk State University, Pirogova Str. 1, Novosibirsk 630090, Russia
* Correspondence: kirilyuk@nioch.nsc.ru

Table of content

NMR Spectra 4
^{1}H NMR (300 MHz; CD_3OD; Zn/CF_3COOH) of 2,2,5,5-tetraethyl-3,4-bis((methylsulfonyloxy)methyl)pyrrolidin-1-oxyl (3) 4
^{1}H NMR (300 MHz; CD3OD; Zn/CF3COOH) of 2,2,5,5-tetraethyl-3-(hydroxymethyl)-4-((methylsulfonyloxy)methyl)pyrrolidin-1-oxyl (4) 4
^{13}C{^{1}H} (75 MHz; CD3OD; Zn/CF3COOH) of 2,2,5,5-tetraethyl-3-(hydroxymethyl)-4-((methylsulfonyloxy)methyl)pyrrolidin-1-oxyl (4) 5
1H NMR (300 MHz; CD3OD, Zn/CF3COOH) of 3-(Aminomethyl)-2,2,5,5-tetraethyl-4-(hydroxymethyl)pyrrolidin-1-oxyl (6) 5
^{1}H NMR (300 MHz; $CDCl_3$) of 3-(aminomethyl)-4-((dimethylamino)methyl)-2,2,5,5-tetraethylpyrrolidin-1-oxyl (14a) 6
^{13}C{1H} NMR (100 MHz, $CDCl_3$) of 3-(aminomethyl)-4-((dimethylamino)methyl)-2,2,5,5-tetraethylpyrrolidin-1-oxyl (14a) 6
^{1}H NMR (300 MHz; CD_3OD, Zn/CF_3COOH) of 3-(aminomethyl)-4-((dimethylamino)methyl)-2,2,5,5-tetraethyl-2,5-dihydro-1H-pyrrol-1-oxyl (14b) 7
^{1}H NMR (300 MHz; CD_3OD, Zn/CF_3COOH) of 3,4-bis(carboxymethyl)-2,2,5,5-tetraethylpyrrolidin-1-oxyl (23) 7
^{13}C{^{1}H} NMR (75 MHz; CD_3OD, Zn/CF_3COOH) of 3,4-bis(carboxymethyl)-2,2,5,5-tetraethylpyrrolidin-1-oxyl (23) 8
^{1}H NMR (400 MHz; CD_3OD, Zn/CF_3COOH) of 3,4-bis(cyanomethyl)-2,2,5,5-tetraethylpyrrolidin-1-oxyl (24) 8
^{13}C{^{1}H} NMR (100 MHz; CD_3OD, Zn/CF_3COOH) of 3,4-bis(cyanomethyl)-2,2,5,5-tetraethylpyrrolidin-1-oxyl (24) 9
IR Spectra 10

IR (KBr) of 2,2,5,5-tetraethyl-3,4-bis((methylsulfonyloxy)methyl)pyrrolidin-1-oxyl (3) 10
IR (KBr) of 2,2,5,5-tetraethyl-3-(hydroxymethyl)-4-((methylsulfonyloxy)methyl)pyrrolidin-1-oxyl (4)..10
IR (KBr) of 3-(((tert-Butoxycarbonyl)amino)methyl)-2,2,5,5-tetraethyl-4-(((methylsulfonyl)oxy)methyl)pyrrolidin-1-oxyl (9) 10
IR (KBr)of 3-(Azidomethyl)-2,2,5,5-tetraethyl-4-(hydroxymethyl)pyrrolidin-1-oxyl (5)....11
IR (KBr) of 3-(Azidomethyl)-4-((tert-butoxycarbonylamino)methyl)-2,2,5,5-tetraethylpyrrolidin-1-oxyl (10) 11
IR (KBr) of 3,4-Bis(azidomethyl)-2,2,5,5-tetraethylpyrrolidin-1-oxyl (12a)....11
IR (KBr) of 3,4-Bis(azidomethyl)-2,2,5,5-tetraethyl-2,5-dihydro-1H-pyrrol-1-oxyl (12b)....12
IR (KBr) of 3-(Aminomethyl)-2,2,5,5-tetraethyl-4-(hydroxymethyl)pyrrolidin-1-oxyl (6) 12
IR (KBr) of 3-(((tert-Butoxycarbonyl)amino)methyl)-2,2,5,5-tetraethyl-4-(hydroxymethyl)pyrrolidin-1-oxyl (8)....12
IR (KBr) of 3-(azidomethyl)-4-((dimethylamino)methyl)-2,2,5,5-tetraethylpyrrolidin-1-oxyl (11a) 13
IR (neat) of -(azidomethyl)-4-((dimethylamino)methyl)-2,2,5,5-tetraethyl-2,5-dihydro-1H-pyrrol-1-oxyl (11b) 13
IR (KBr) of 3-((2,5-Dioxo-2,5-dihydro-1H-pyrrol-1-yl)methyl)-2,2,5,5-tetraethyl-4-((trimethylammonio)methyl)pyrrolidin-1-oxyl monoiodide (16a)....13
IR (KBr) of 3-((2,5-Dioxo-2,5-dihydro-1H-pyrrol-1-yl)methyl)-2,2,5,5-tetraethyl-4-((trimethylammonio)methyl)-2,5-dihydro-1H-pyrrol-1-oxyl monoiodide (16b)....14
IR (KBr) of 3-(azidomethyl)-2,2,5,5-tetraethyl-4-((trimethylammonio)methyl)pyrrolidin-1-oxyl monoiodide (17a)....14
IR (KBr) of 3-(azidomethyl)-2,2,5,5-tetraethyl-4-((trimethylammonio)methyl)-2,5-dihydro-1H-pyrrol-1-oxyl monoiodide (17b) 14
IR (KBr) of 3-(azidomethyl)-4-((4,5-bis(methoxycarbonyl)-1H-1,2,3-triazol-1-yl)methyl)-2,2,5,5-tetraethylpyrrolidin-1-oxyl (18)....15
IR (KBr) of 3,4-bis((4,5-bis(methoxycarbonyl)-1H-1,2,3-triazol-1-yl)methyl)-2,2,5,5-tetraethylpyrrolidin-1-oxyl (19)....15
IR (KBr) of 3-(aminomethyl)-4-(azidomethyl)-2,2,5,5-tetraethylpyrrolidin-1-oxyl (13a)....15
IR (neat) of 3-(aminomethyl)-4-(azidomethyl)-2,2,5,5-tetraethyl-2,5-dihydro-1H-pyrrol-1-oxyl (13b) .16
IR (neat) of 3-(aminomethyl)-4-((dimethylamino)methyl)-2,2,5,5-tetraethylpyrrolidin-1-oxyl (14a)....16
IR (neat) of 3-(aminomethyl)-4-((dimethylamino)methyl)-2,2,5,5-tetraethyl-2,5-dihydro-1H-pyrrol-1-oxyl (14b)....16
IR (KBr) of 3-((2,5-dioxo-2,5-dihydro-1H-pyrrol-1-yl)methyl)-2,2,5,5-tetraethyl-4-(hydroxymethyl)pyrrolidin-1-oxyl (7) 17
IR (KBr) of 3-((dimethylamino)methyl)-4-((2,5-dioxo-2,5-dihydro-1H-pyrrol-1-yl)methyl)-2,2,5,5-tetraethylpyrrolidin-1-oxyl (15a)....17
IR (KBr) of 3-((dimethylamino)methyl)-4-((2,5-dioxo-2,5-dihydro-1H-pyrrol-1-yl)methyl)-2,2,5,5-tetraethyl-2,5-dihydro-1H-pyrrol-1-oxyl (15b)....17
IR (KBr) of 3-(azidomethyl)-4-((4,5-dicarboxy-1H-1,2,3-triazol-1-yl)methyl)-2,2,5,5-tetraethylpyrrolidin-1-oxyl monosodium salt (20)....18
IR (KBr) of 3,4-bis(carboxymethyl)-2,2,5,5-tetraethylpyrrolidin-1-oxyl (23)....18
XRD Data 19
Table 1 19
EPR Spectra 23
EPR of 3-(Azidomethyl)-2,2,5,5-tetraethyl-4-(hydroxymethyl)pyrrolidin-1-oxyl (5)....23
EPR of 3-(Aminomethyl)-2,2,5,5-tetraethyl-4-(hydroxymethyl)pyrrolidin-1-oxyl (6) 23
EPR of 3-((2,5-dioxo-2,5-dihydro-1*H*-pyrrol-1-yl)methyl)-2,2,5,5-tetraethyl-4-(hydroxymethyl)pyrrolidin-1-oxyl (7) 23
EPR of 3-(((tert-Butoxycarbonyl)amino)methyl)-2,2,5,5-tetraethyl-4-(hydroxymethyl)pyrrolidin-1-oxyl (8) 23
EPR of 3-(((tert-Butoxycarbonyl)amino)methyl)-2,2,5,5-tetraethyl-4-(((methylsulfonyl)oxy)methyl)pyrrolidin-1-oxyl (9) 23
EPR of 3-(Azidomethyl)-4-((tert-butoxycarbonylamino)methyl)-2,2,5,5-tetraethylpyrrolidin-1-oxyl (10) 23

EPR of 3-(azidomethyl)-4-((dimethylamino)methyl)-2,2,5,5-tetraethylpyrrolidin-1-oxyl (11a) 24
EPR of 3-(azidomethyl)-4-((dimethylamino)methyl)-2,2,5,5-tetraethyl-2,5-dihydro-1H-pyrrol-1-oxyl (11b) 24
EPR of 3-(aminomethyl)-4-(azidomethyl)-2,2,5,5-tetraethylpyrrolidin-1-oxyl (13a) 24
EPR of 3-(aminomethyl)-4-(azidomethyl)-2,2,5,5-tetraethyl-2,5-dihydro-1H-pyrrol-1-oxyl (13b) 24
EPR of 3-(aminomethyl)-4-((dimethylamino)methyl)-2,2,5,5-tetraethylpyrrolidin-1-oxyl (14a) 24
EPR of 3-(aminomethyl)-4-((dimethylamino)methyl)-2,2,5,5-tetraethyl-2,5-dihydro-1H-pyrrol-1-oxyl (14b) 24
EPR of 3-((dimethylamino)methyl)-4-((2,5-dioxo-2,5-dihydro-1H-pyrrol-1-yl)methyl)-2,2,5,5-tetraethylpyrrolidin-1-oxyl (15a) 25
EPR of 3-((dimethylamino)methyl)-4-((2,5-dioxo-2,5-dihydro-1H-pyrrol-1-yl)methyl)-2,2,5,5-tetraethyl-2,5-dihydro-1H-pyrrol-1-oxyl (15b) 25
EPR of 3-((2,5-Dioxo-2,5-dihydro-1H-pyrrol-1-yl)methyl)-2,2,5,5-tetraethyl-4-((trimethylammonio)methyl)pyrrolidin-1-oxyl monoiodide (16a) 25
EPR of 3-((2,5-Dioxo-2,5-dihydro-1H-pyrrol-1-yl)methyl)-2,2,5,5-tetraethyl-4-((trimethylammonio)methyl)-2,5-dihydro-1H-pyrrol-1-oxyl monoiodide (16b) 25
EPR of 3-(azidomethyl)-2,2,5,5-tetraethyl-4-((trimethylammonio)methyl)pyrrolidin-1-oxyl monoiodide (17a) 25
EPR of 3-(azidomethyl)-2,2,5,5-tetraethyl-4-((trimethylammonio)methyl)-2,5-dihydro-1H-pyrrol-1-oxyl monoiodide (17b) 25
EPR of 3-(azidomethyl)-4-((4,5-dicarboxy-1H-1,2,3-triazol-1-yl)methyl)-2,2,5,5-tetraethylpyrrolidin-1-oxyl monosodium salt (20) 26
EPR of 3-carboxy-2,2,5,5-tetraethylpyrrolidin-1-oxyl (21) 26
EPR of 3,4-bis(hydroxymethyl)-2,2,5,5-tetraethyl-2,5-dihydro-1H-pyrrol-1-oxyl (22) 26
EPR of 3,4-bis(carboxymethyl)-2,2,5,5-tetraethylpyrrolidin-1-oxyl (23) 26

NMR Spectra

^{1}H NMR (300 MHz; CD_3OD; Zn/CF_3COOH) of 2,2,5,5-tetraethyl-3,4-bis((methylsulfonyloxy)methyl)pyrrolidin-1-oxyl (3)

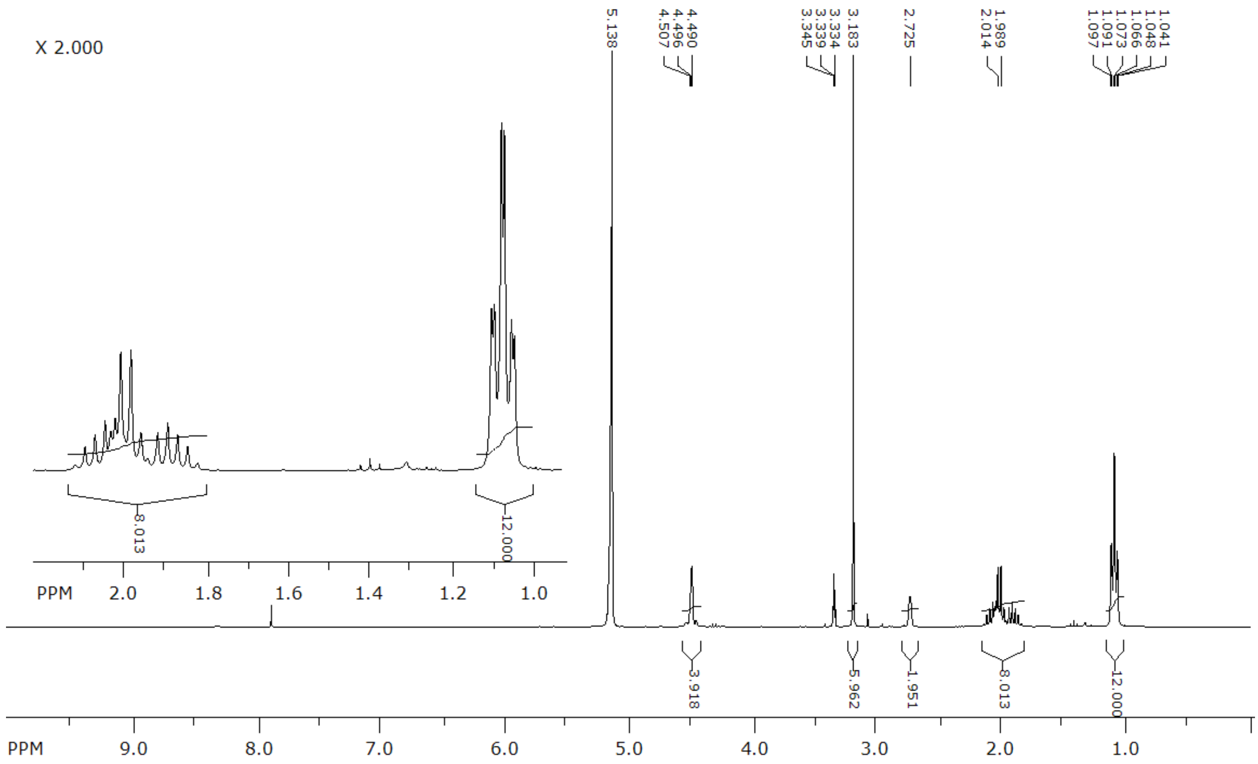

^{1}H NMR (300 MHz; CD3OD; Zn/CF3COOH) of 2,2,5,5-tetraethyl-3-(hydroxymethyl)-4-((methylsulfonyloxy)methyl)pyrrolidin-1-oxyl (4)

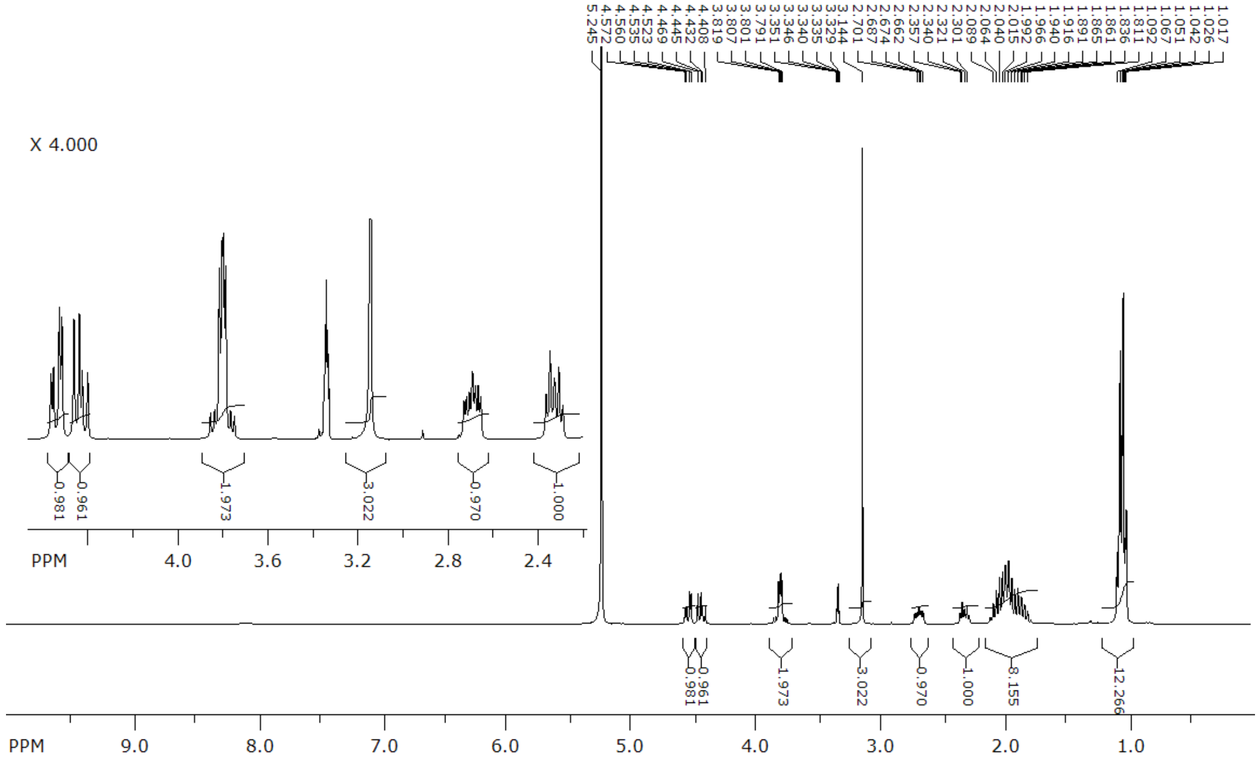

$^{13}C\{^1H\}$ (75 MHz; CD3OD; Zn/CF3COOH) of 2,2,5,5-tetraethyl-3-(hydroxymethyl)-4-((methylsulfonyloxy)methyl)pyrrolidin-1-oxyl (4)

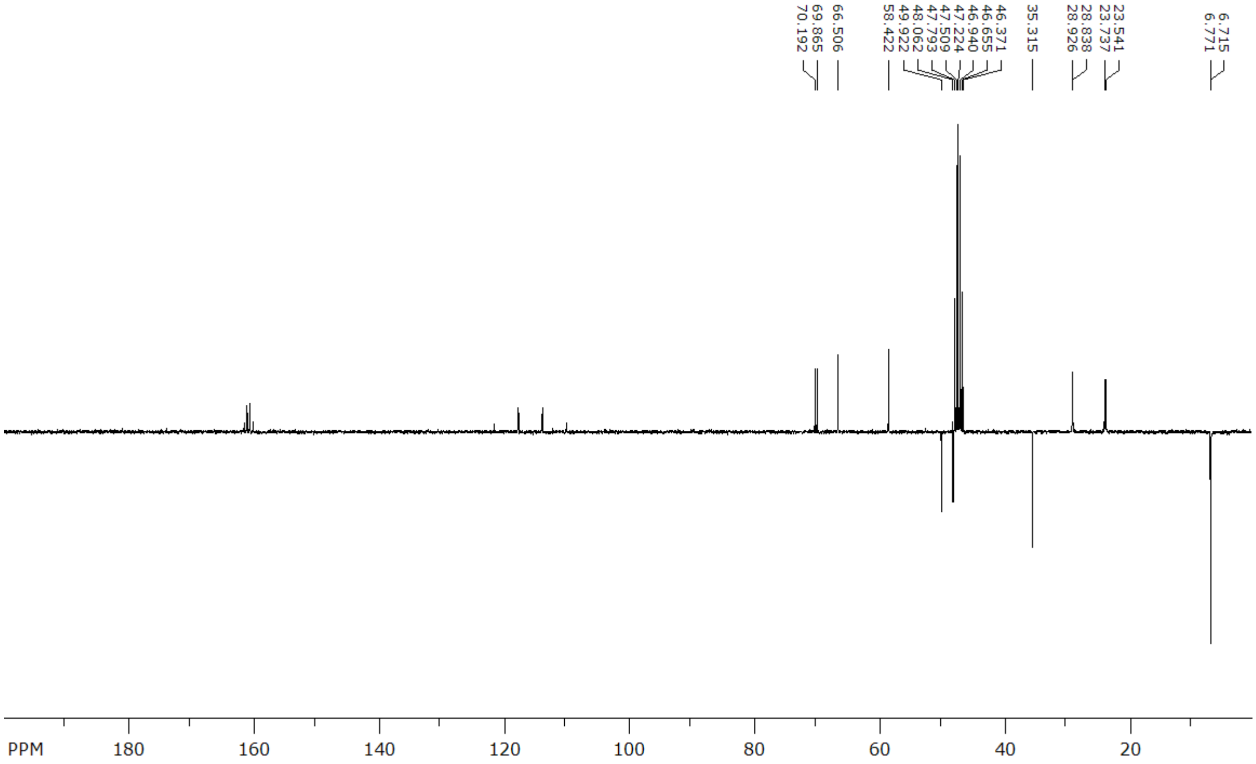

1H NMR (300 MHz; CD3OD, Zn/CF3COOH) of 3-(Aminomethyl)-2,2,5,5-tetraethyl-4-(hydroxymethyl)pyrrolidin-1-oxyl (6)

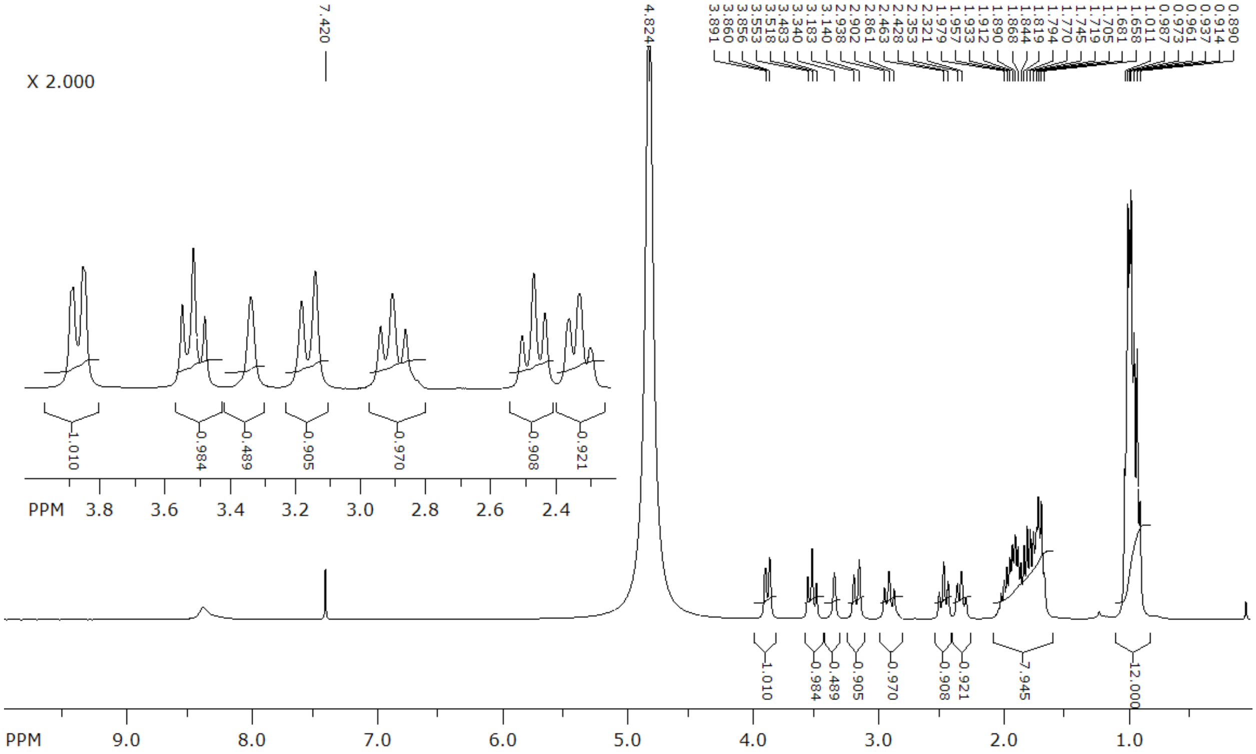

^{1}H NMR (300 MHz; $CDCl_3$) of 3-(aminomethyl)-4-((dimethylamino)methyl)-2,2,5,5-tetraethylpyrrolidin-1-oxyl (14a)

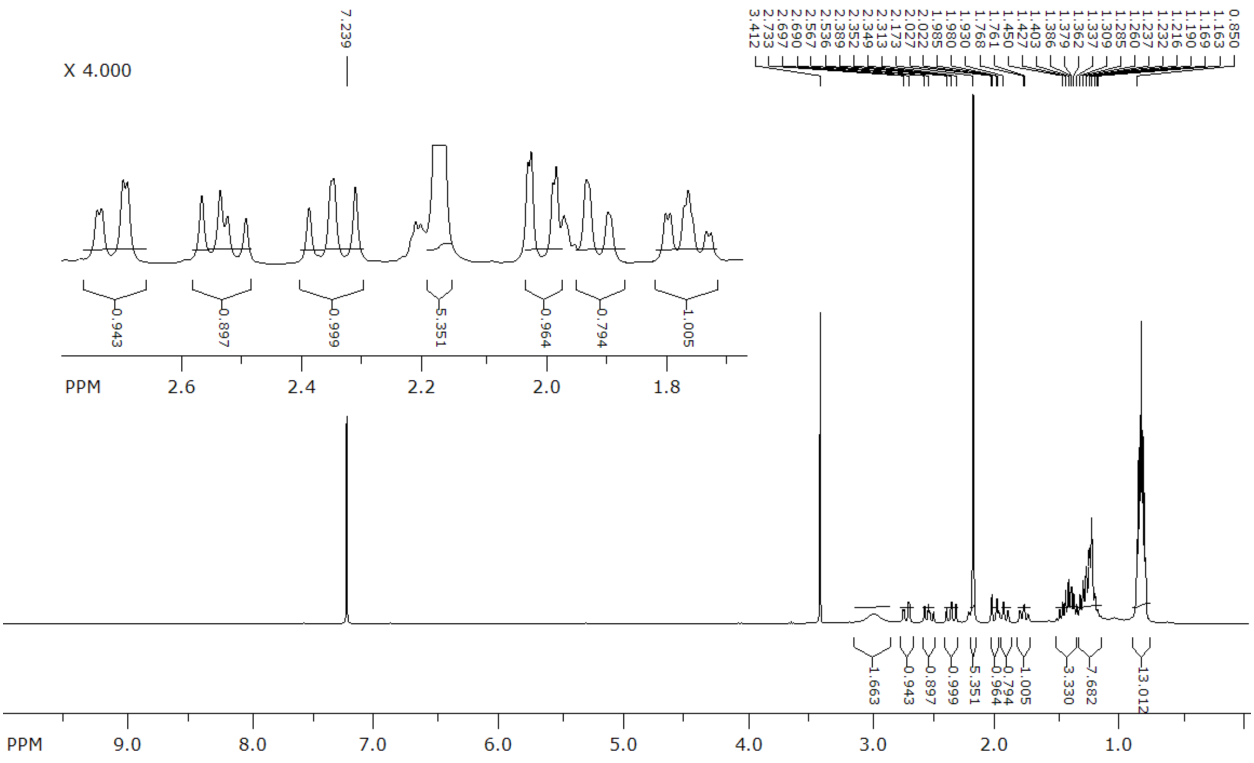

$^{13}C\{^{1}H\}$ NMR (100 MHz, $CDCl_3$) of 3-(aminomethyl)-4-((dimethylamino)methyl)-2,2,5,5-tetraethylpyrrolidin-1-oxyl (14a)

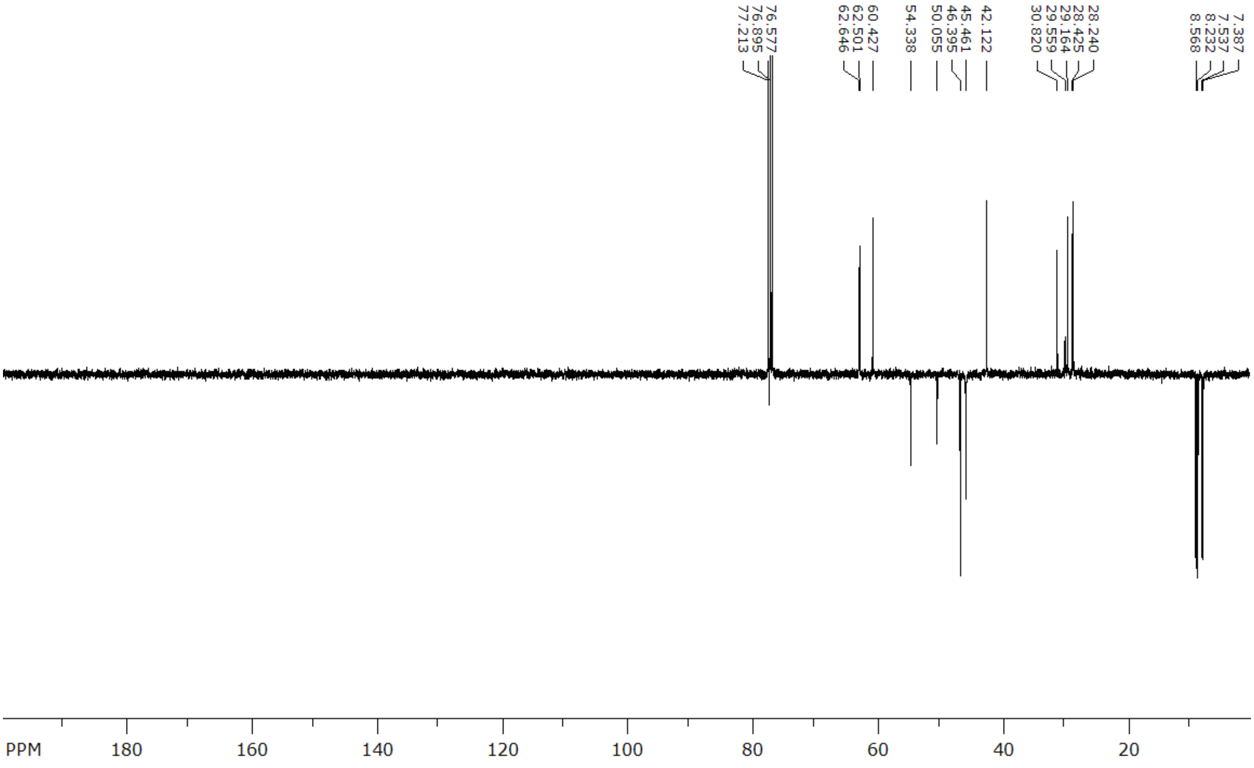

^{1}H NMR (300 MHz; CD_3OD, Zn/CF_3COOH) of 3-(aminomethyl)-4-((dimethylamino)methyl)-2,2,5,5-tetraethyl-2,5-dihydro-1H-pyrrol-1-oxyl (14b)

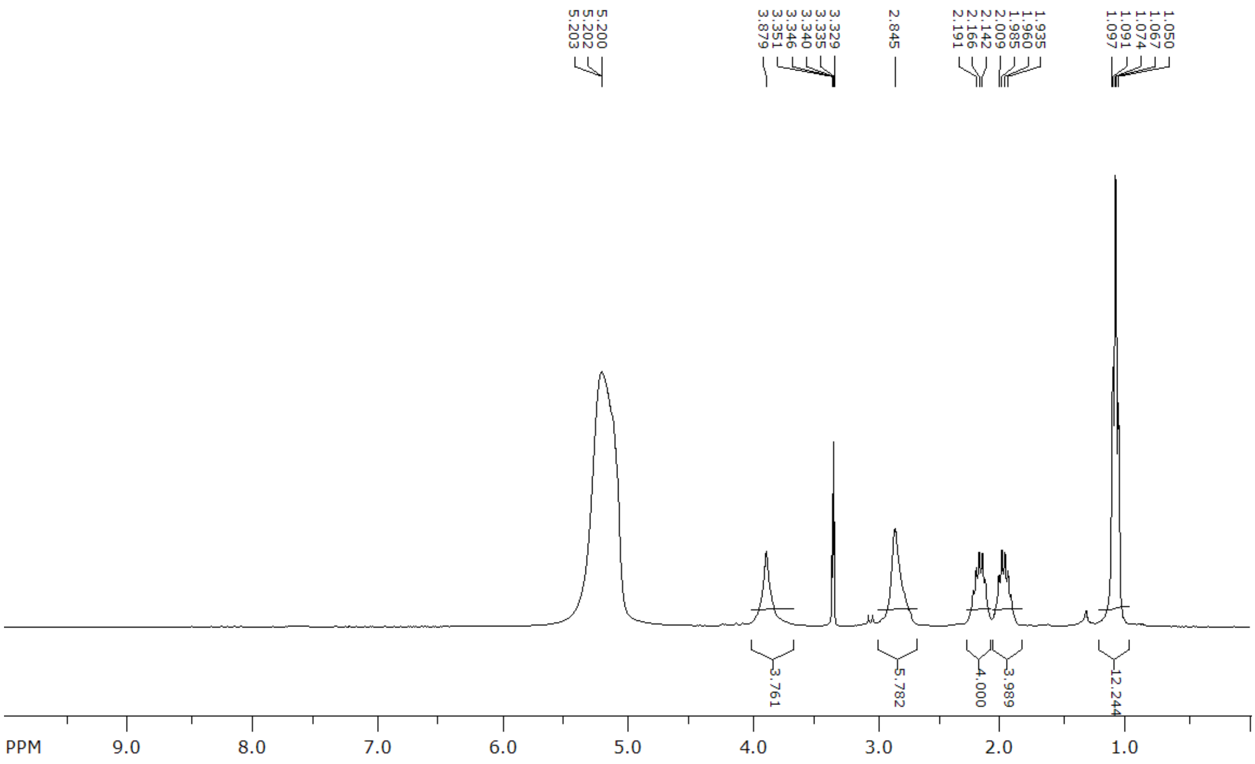

^{1}H NMR (300 MHz; CD_3OD, Zn/CF_3COOH) of 3,4-bis(carboxymethyl)-2,2,5,5-tetraethylpyrrolidin-1-oxyl (23)

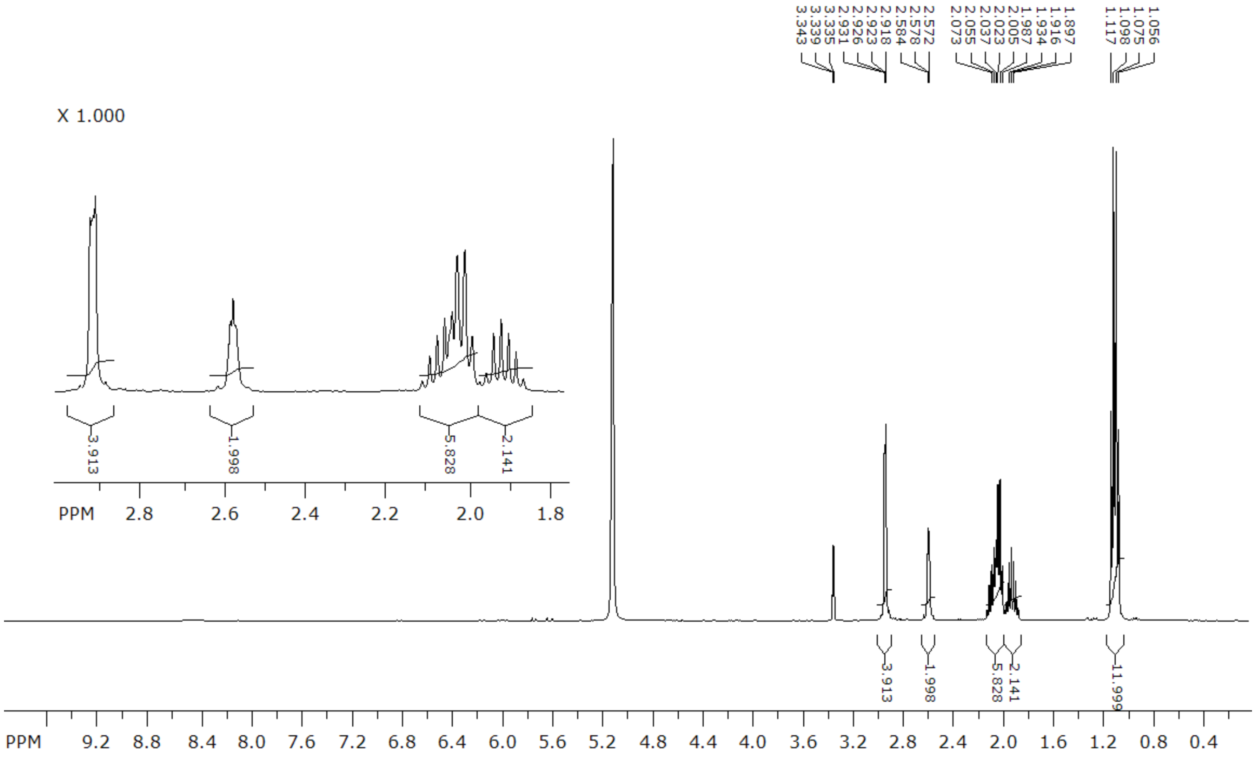

$^{13}C\{^1H\}$ NMR (75 MHz; CD_3OD, Zn/CF_3COOH) of 3,4-bis(carboxymethyl)-2,2,5,5-tetraethylpyrrolidin-1-oxyl (23)

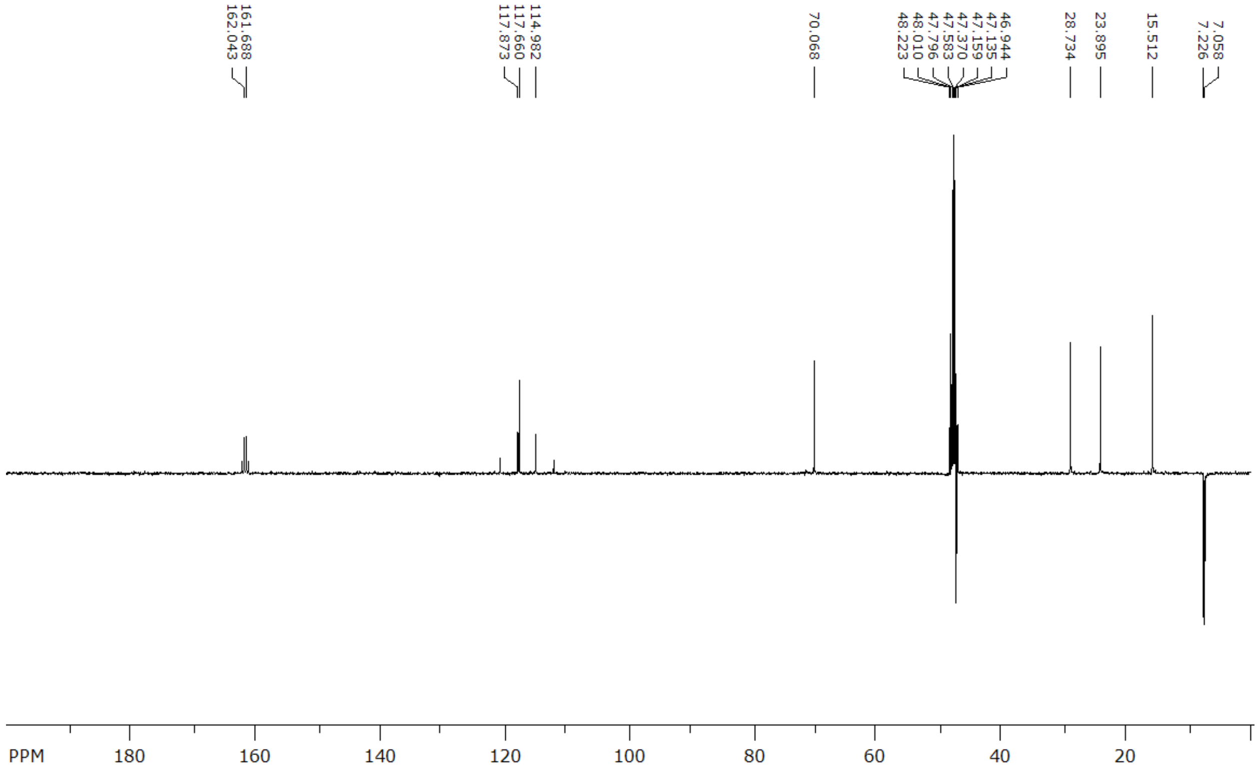

^1H NMR (400 MHz; CD_3OD, Zn/CF_3COOH) of 3,4-bis(cyanomethyl)-2,2,5,5-tetraethylpyrrolidin-1-oxyl (24)

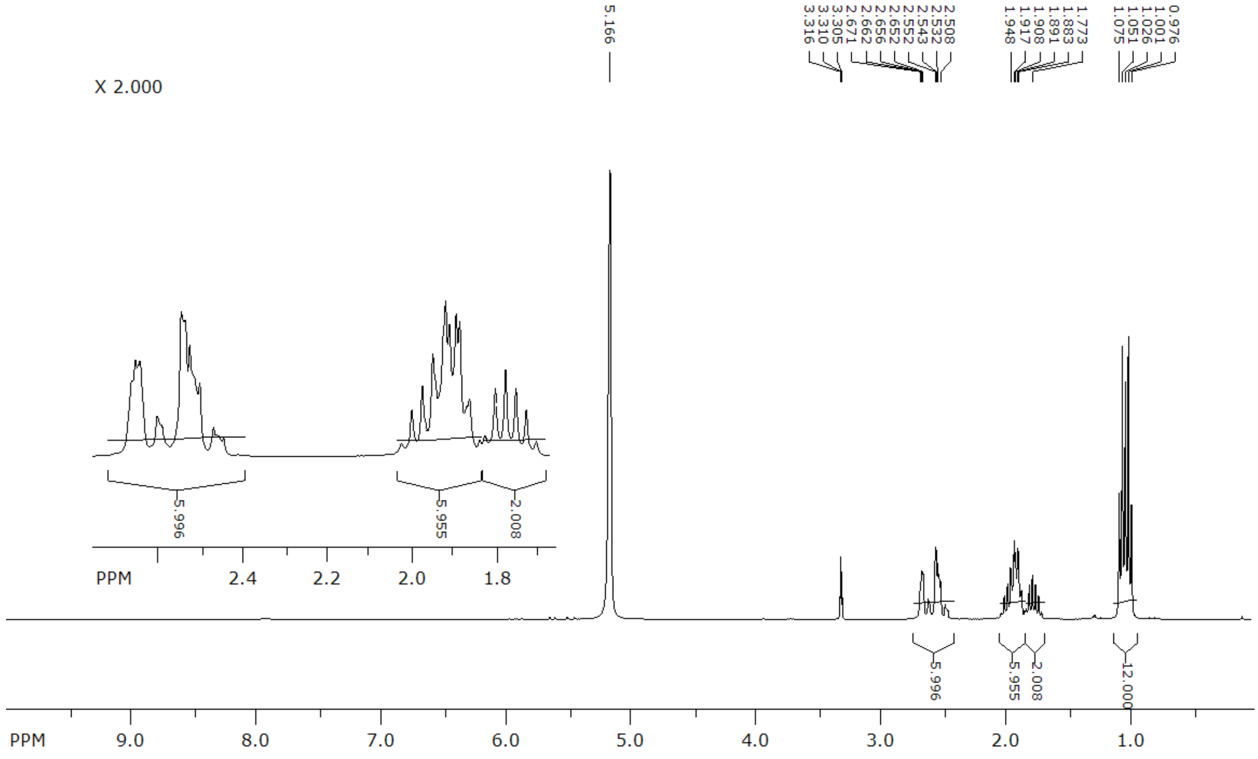

$^{13}C\{^{1}H\}$ NMR (100 MHz; CD_3OD, Zn/CF_3COOH) of 3,4-bis(cyanomethyl)-2,2,5,5-tetraethylpyrrolidin-1-oxyl (24)

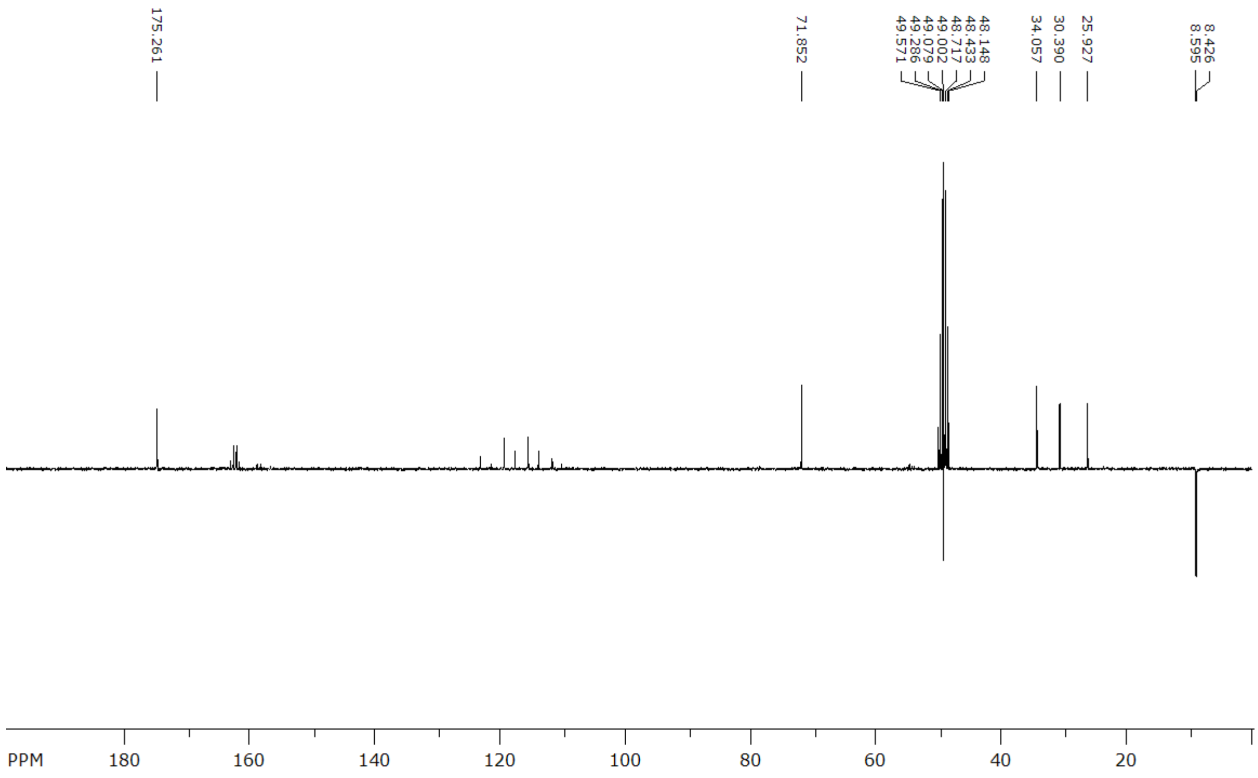

IR Spectra

IR (KBr) of 2,2,5,5-tetraethyl-3,4-bis((methylsulfonyloxy)methyl)pyrrolidin-1-oxyl (3)

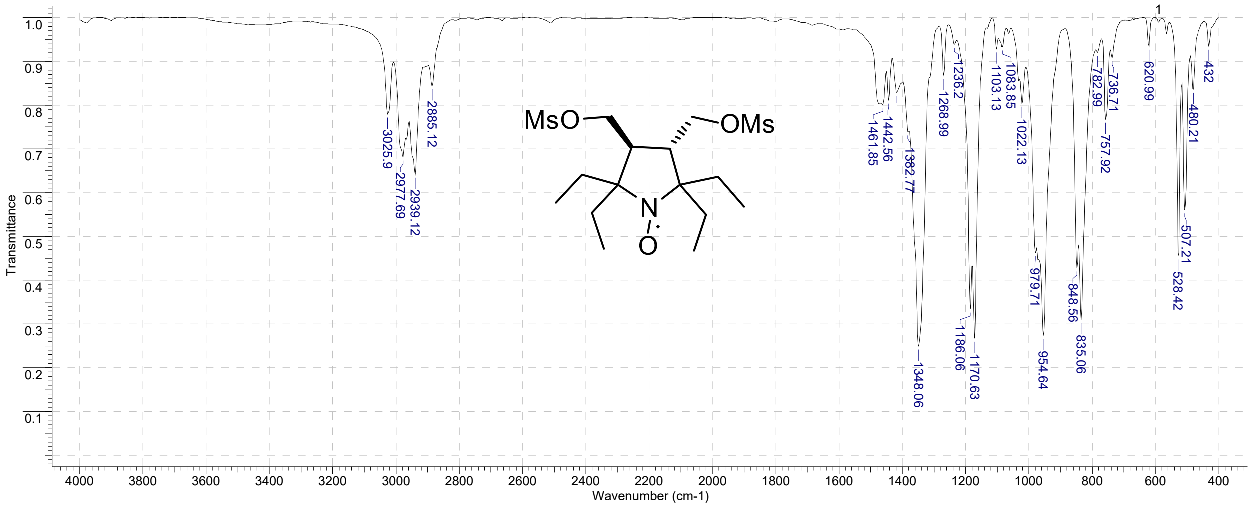

IR (KBr) of 2,2,5,5-tetraethyl-3-(hydroxymethyl)-4-((methylsulfonyloxy)methyl)pyrrolidin-1-oxyl (4)

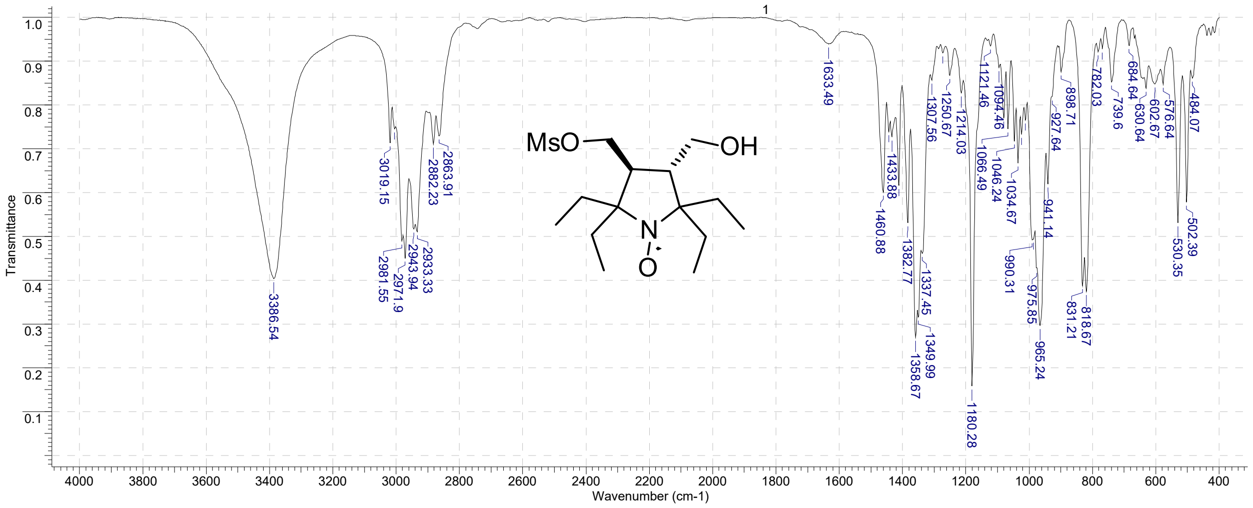

IR (KBr) of 3-(((tert-Butoxycarbonyl)amino)methyl)-2,2,5,5-tetraethyl-4-(((methylsulfonyl)oxy)methyl)pyrrolidin-1-oxyl (9)

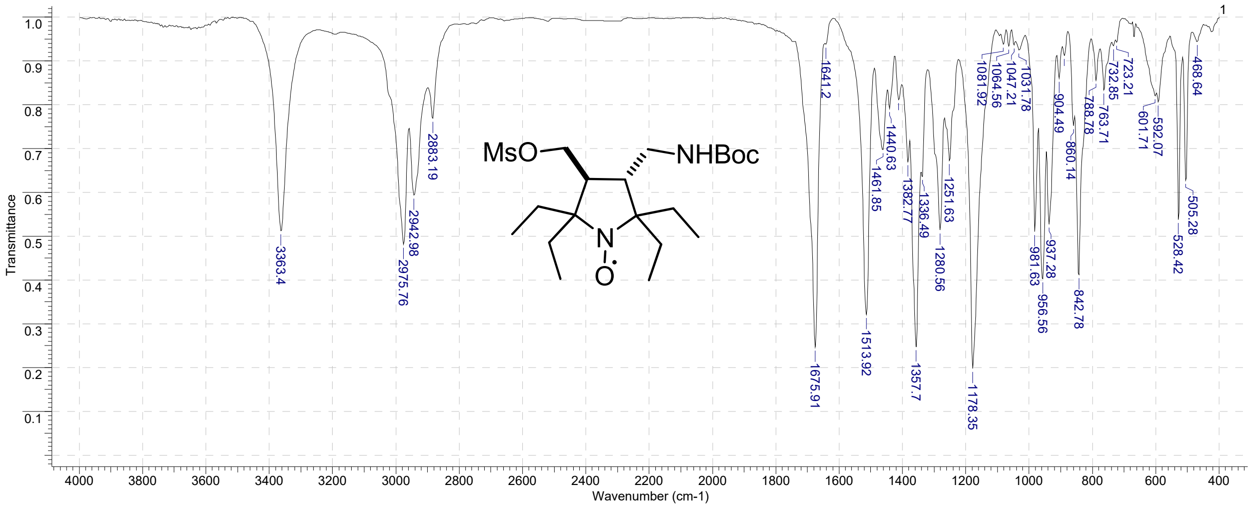

IR (KBr)of 3-(Azidomethyl)-2,2,5,5-tetraethyl-4-(hydroxymethyl)pyrrolidin-1-oxyl (5)

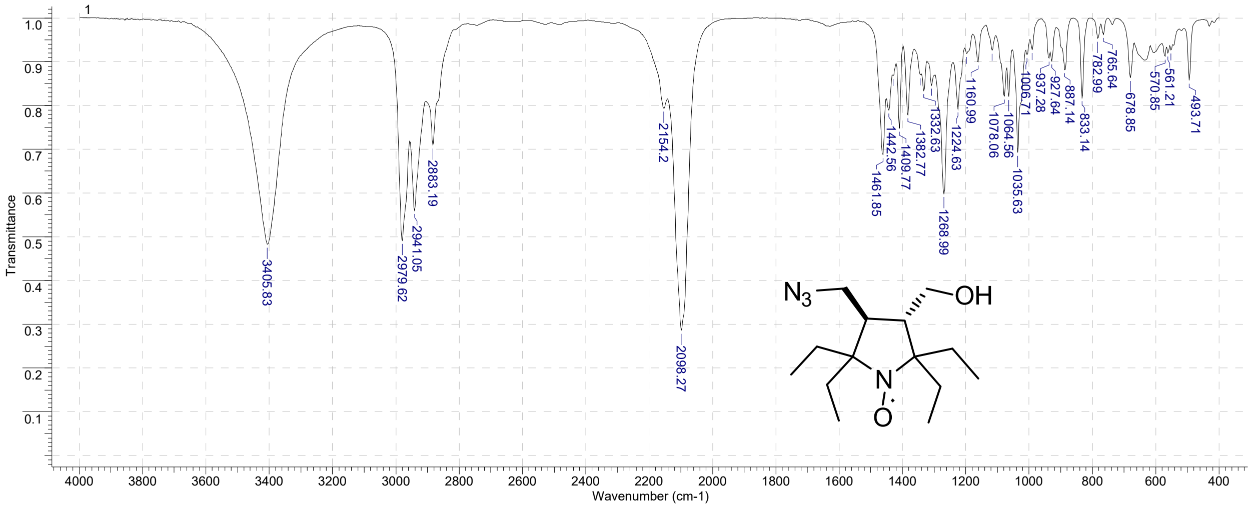

IR (KBr) of 3-(Azidomethyl)-4-((tert-butoxycarbonylamino)methyl)-2,2,5,5-tetraethylpyrrolidin-1-oxyl (10)

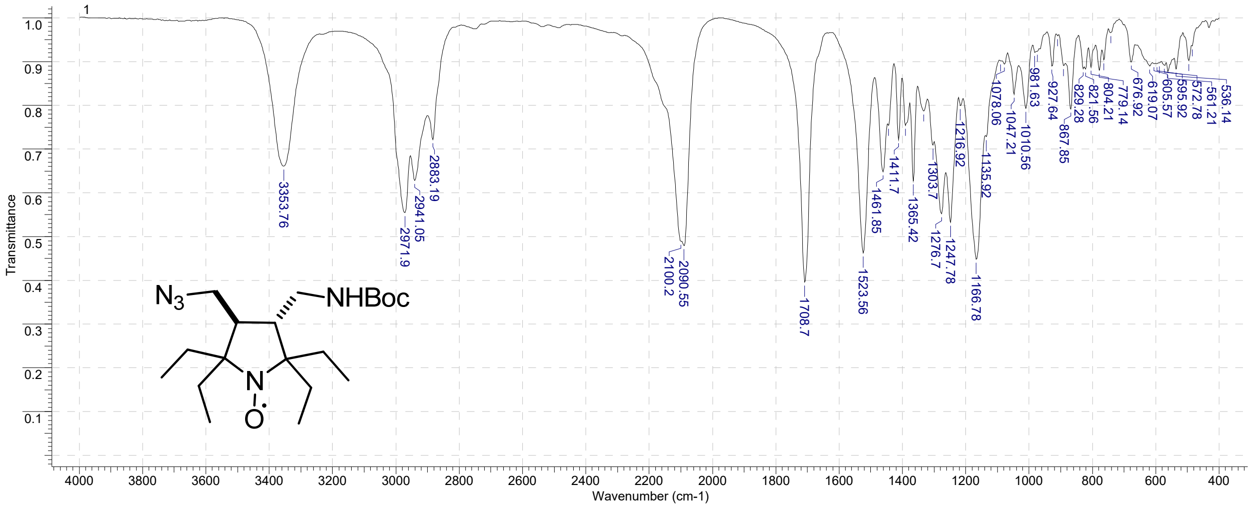

IR (KBr) of 3,4-Bis(azidomethyl)-2,2,5,5-tetraethylpyrrolidin-1-oxyl (12a)

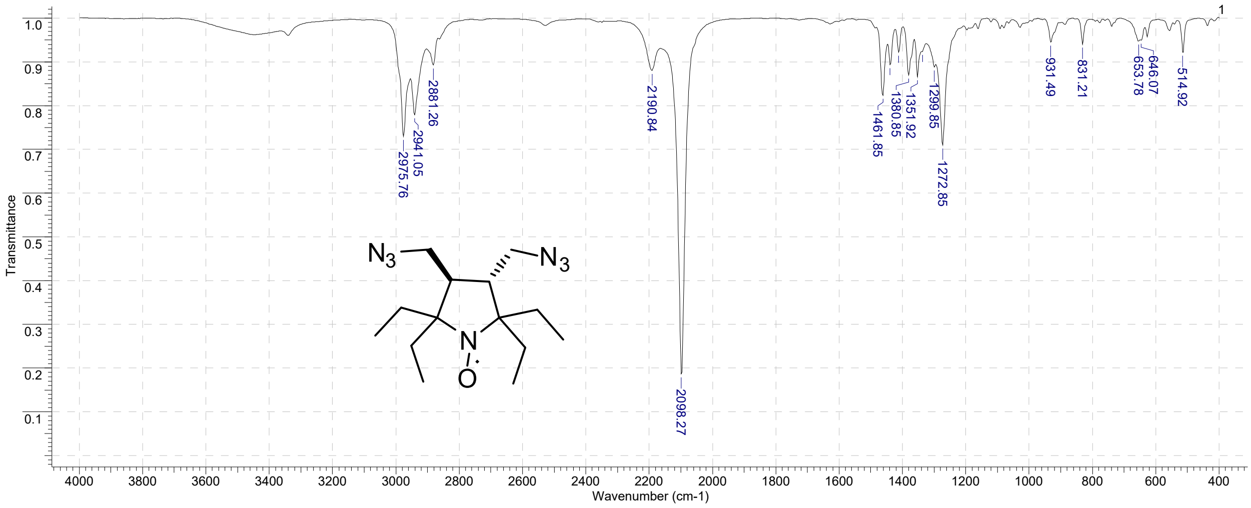

IR (KBr) of 3,4-Bis(azidomethyl)-2,2,5,5-tetraethyl-2,5-dihydro-1H-pyrrol-1-oxyl (12b)

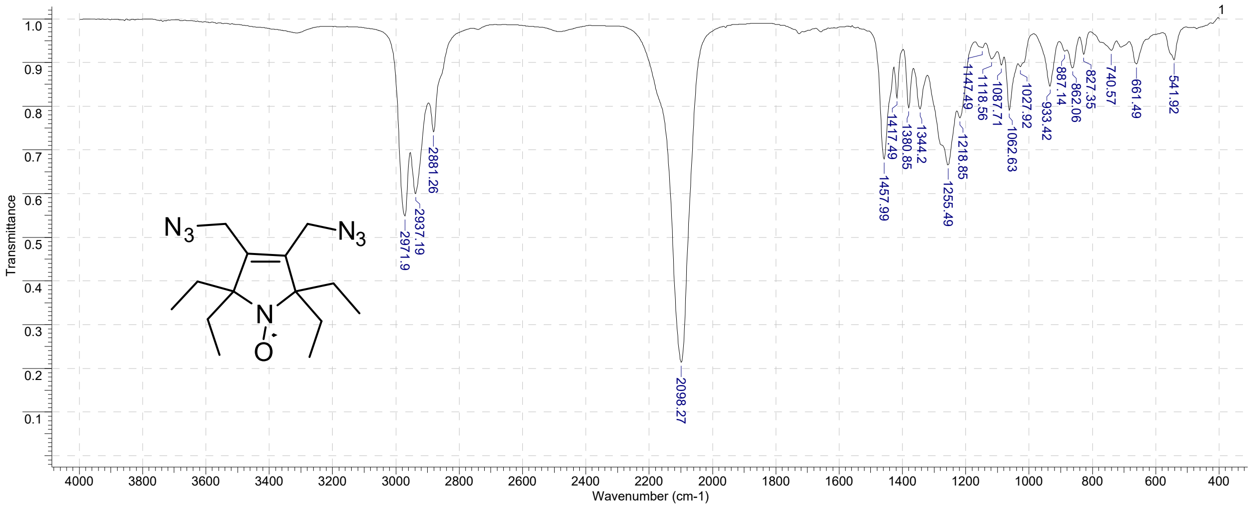

IR (KBr) of 3-(Aminomethyl)-2,2,5,5-tetraethyl-4-(hydroxymethyl)pyrrolidin-1-oxyl (6)

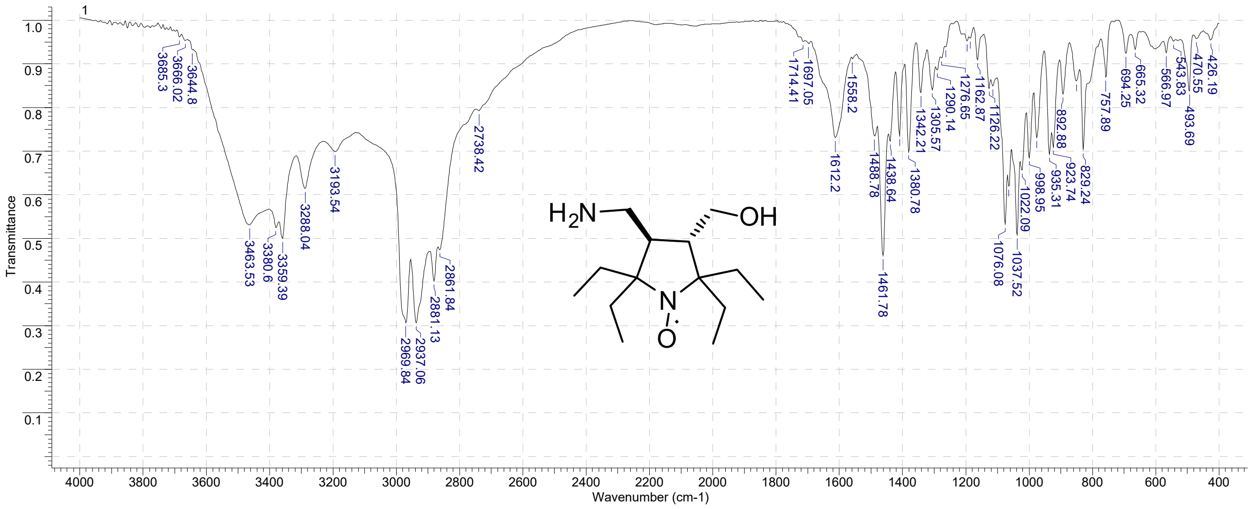

IR (KBr) of 3-(((tert-Butoxycarbonyl)amino)methyl)-2,2,5,5-tetraethyl-4-(hydroxymethyl)pyrrolidin-1-oxyl (8)

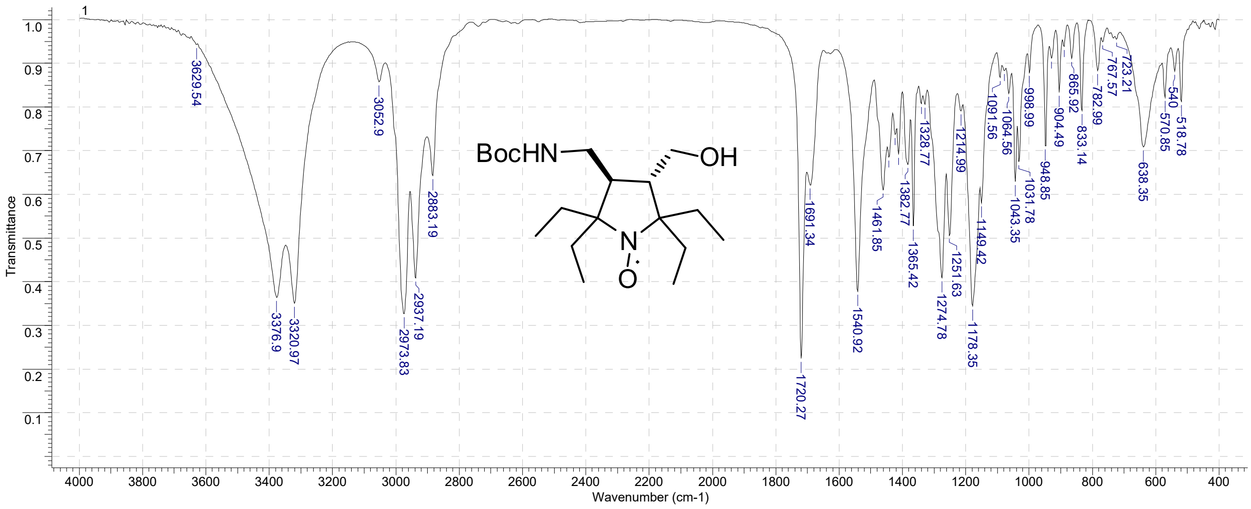

IR (KBr) of 3-(azidomethyl)-4-((dimethylamino)methyl)-2,2,5,5-tetraethylpyrrolidin-1-oxyl (11a)

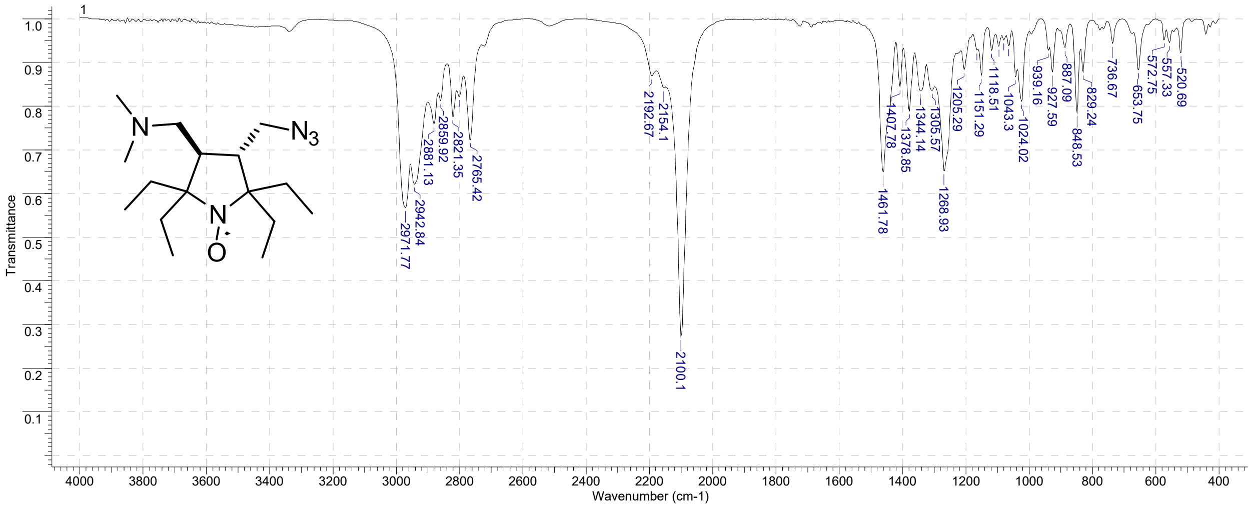

IR (neat) of -(azidomethyl)-4-((dimethylamino)methyl)-2,2,5,5-tetraethyl-2,5-dihydro-1H-pyrrol-1-oxyl (11b)

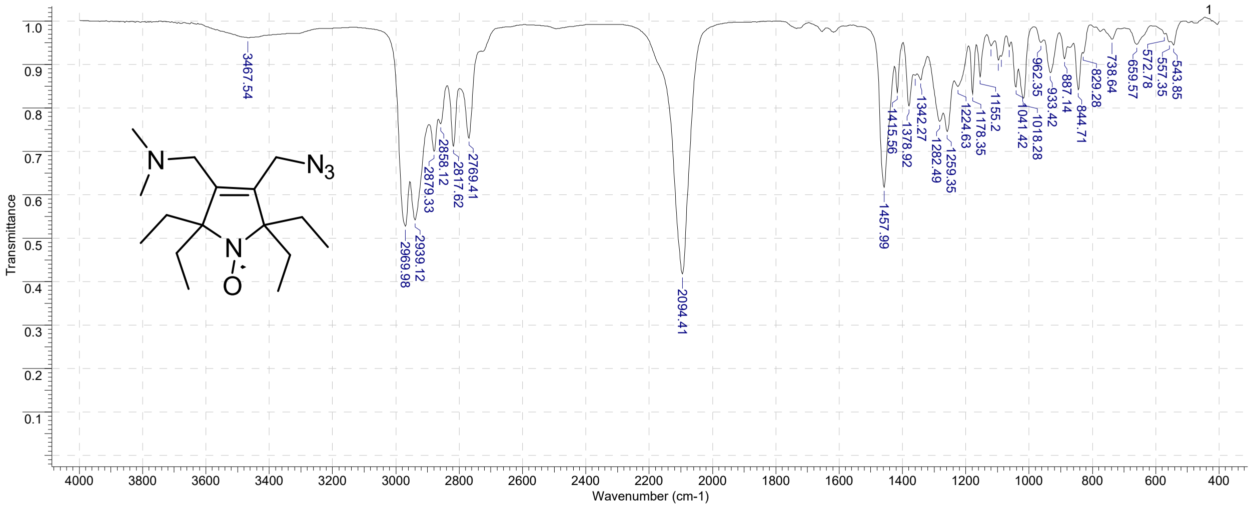

IR (KBr) of 3-((2,5-Dioxo-2,5-dihydro-1H-pyrrol-1-yl)methyl)-2,2,5,5-tetraethyl-4-((trimethylammonio)methyl)pyrrolidin-1-oxyl monoiodide (16a)

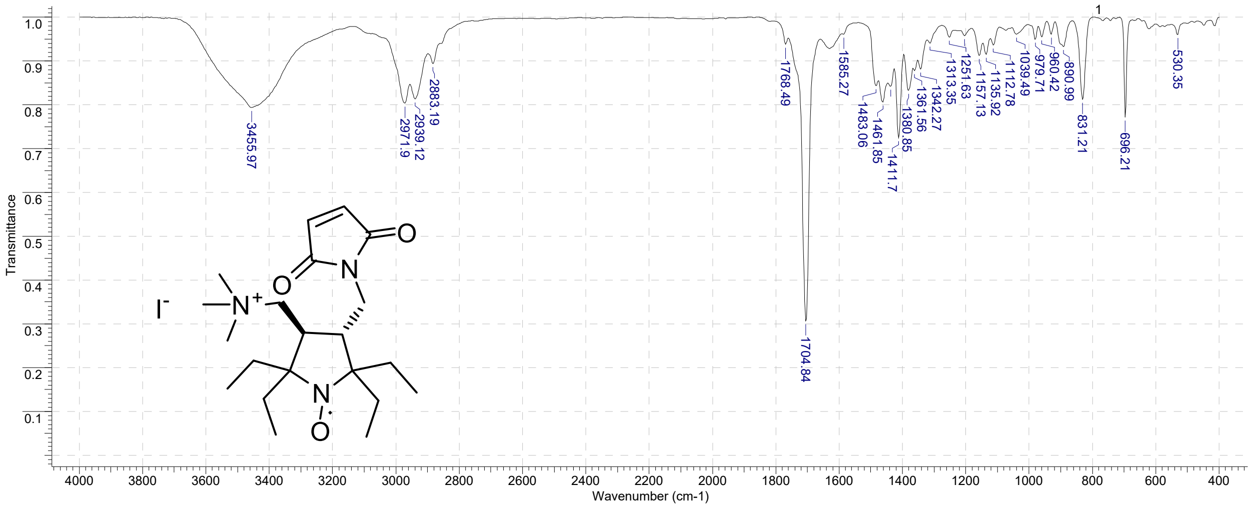

IR (KBr) of 3-((2,5-Dioxo-2,5-dihydro-1H-pyrrol-1-yl)methyl)-2,2,5,5-tetraethyl-4-((trimethylammonio)methyl)-2,5-dihydro-1H-pyrrol-1-oxyl monoiodide (16b)

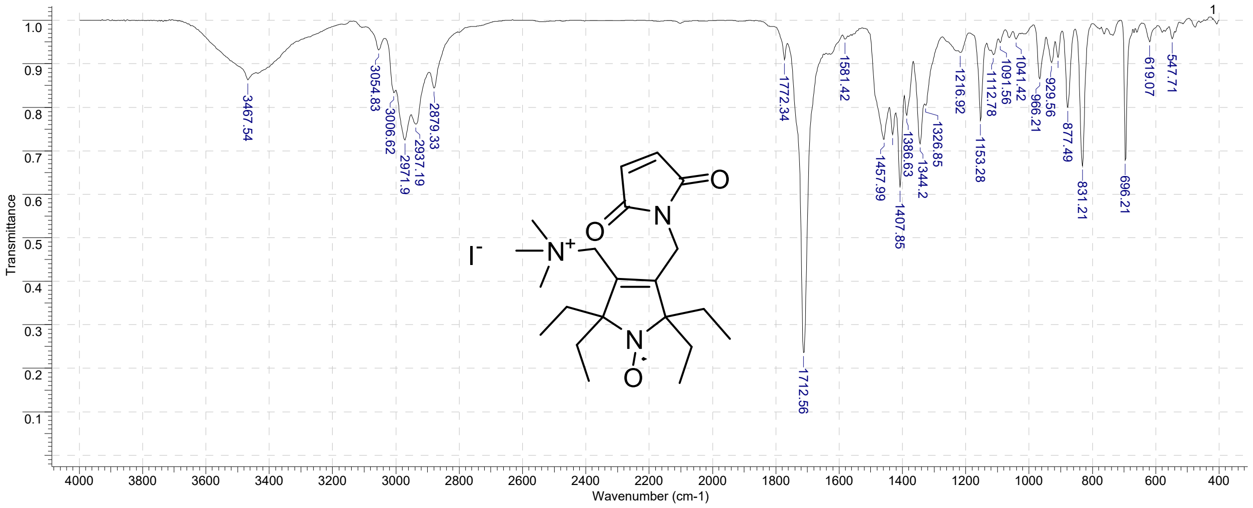

IR (KBr) of 3-(azidomethyl)-2,2,5,5-tetraethyl-4-((trimethylammonio)methyl)pyrrolidin-1-oxyl monoiodide (17a)

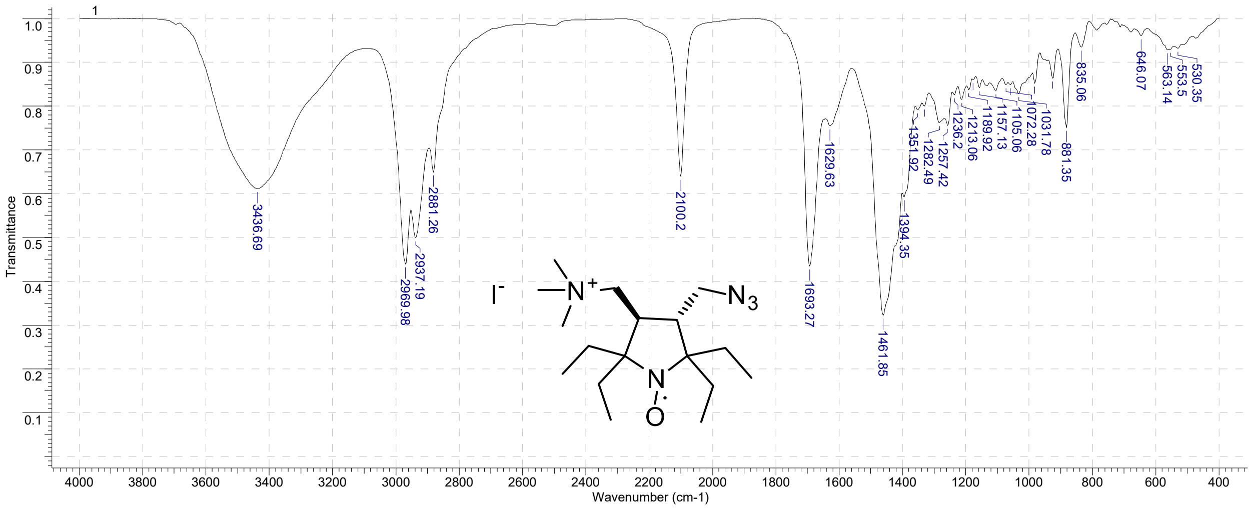

IR (KBr) of 3-(azidomethyl)-2,2,5,5-tetraethyl-4-((trimethylammonio)methyl)-2,5-dihydro-1H-pyrrol-1-oxyl monoiodide (17b)

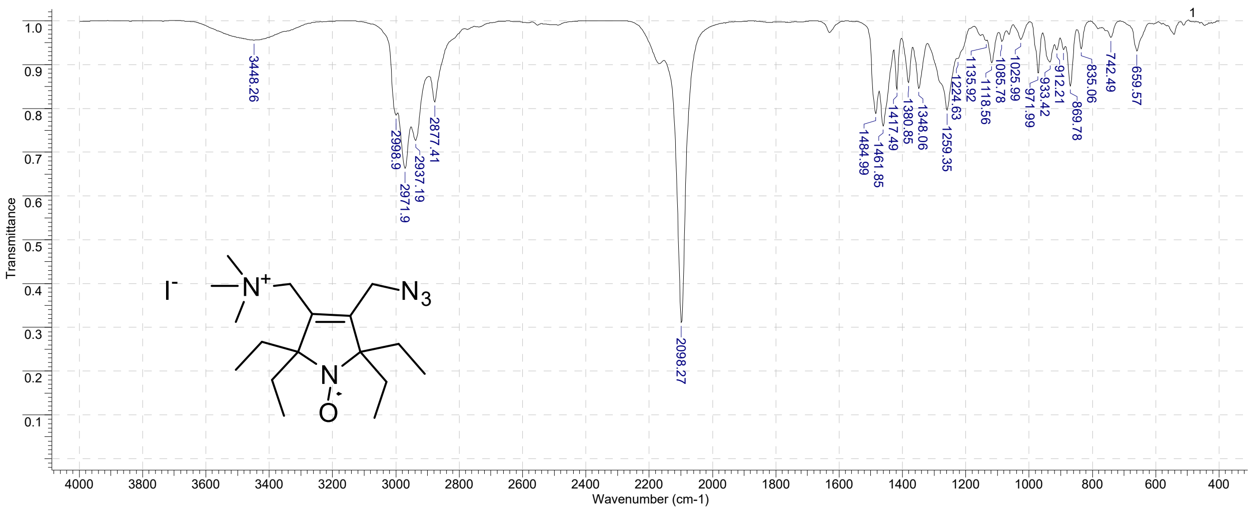

IR (KBr) of 3-(azidomethyl)-4-((4,5-bis(methoxycarbonyl)-1H-1,2,3-triazol-1-yl)methyl)-2,2,5,5-tetraethylpyrrolidin-1-oxyl (18)

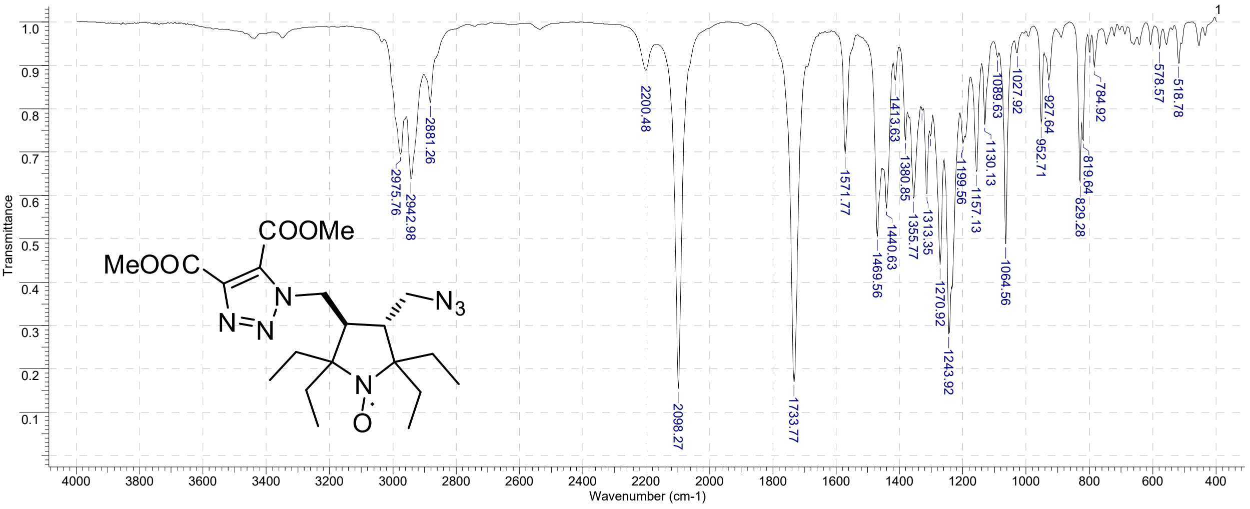

IR (KBr) of 3,4-bis((4,5-bis(methoxycarbonyl)-1H-1,2,3-triazol-1-yl)methyl)-2,2,5,5-tetraethylpyrrolidin-1-oxyl (19)

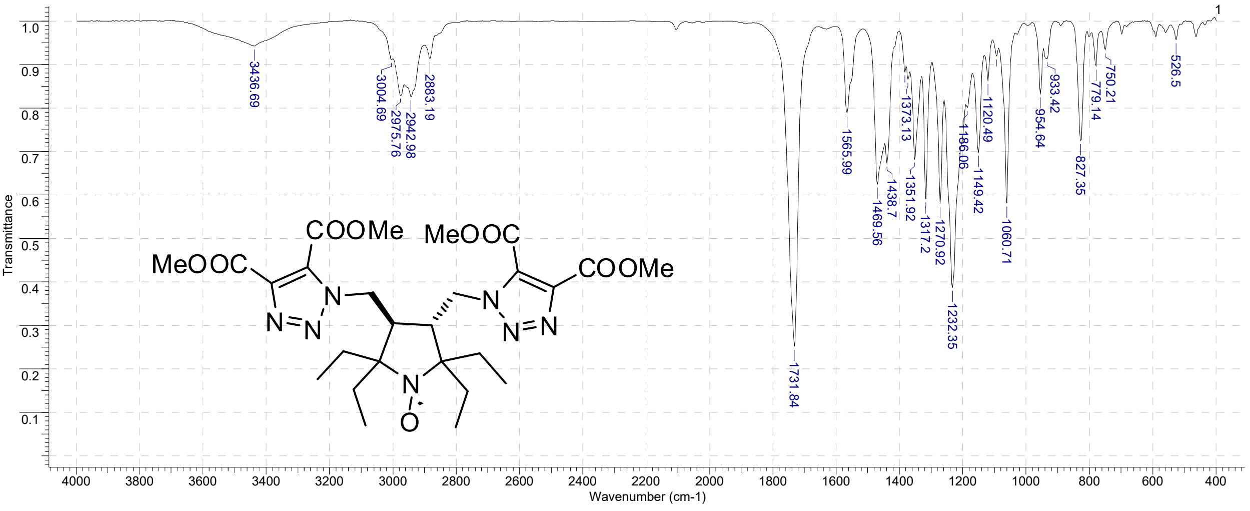

IR (KBr) of 3-(aminomethyl)-4-(azidomethyl)-2,2,5,5-tetraethylpyrrolidin-1-oxyl (13a)

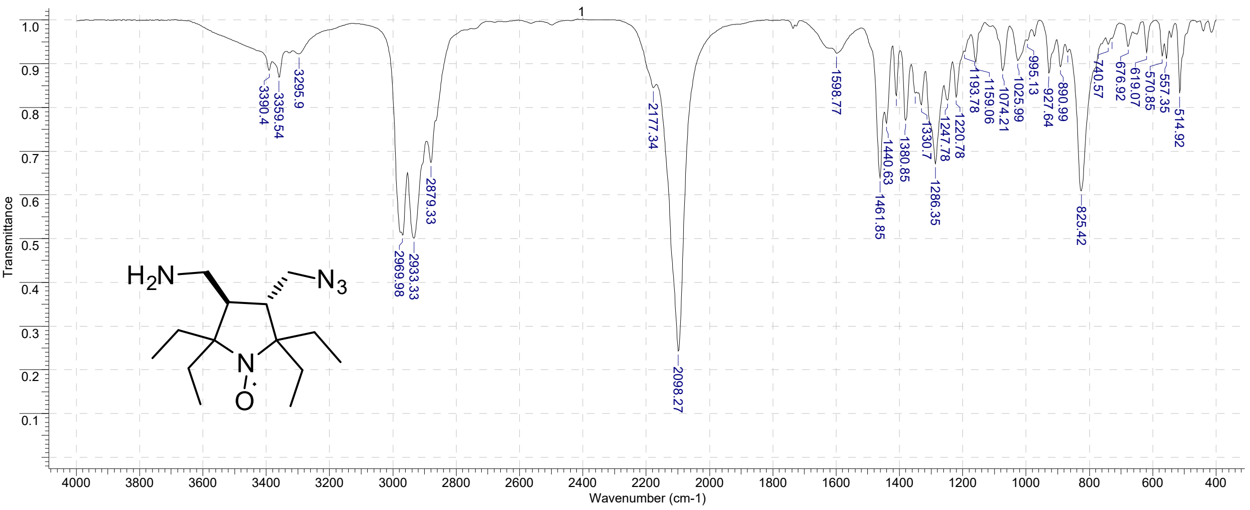

IR (neat) of 3-(aminomethyl)-4-(azidomethyl)-2,2,5,5-tetraethyl-2,5-dihydro-1H-pyrrol-1-oxyl (13b)

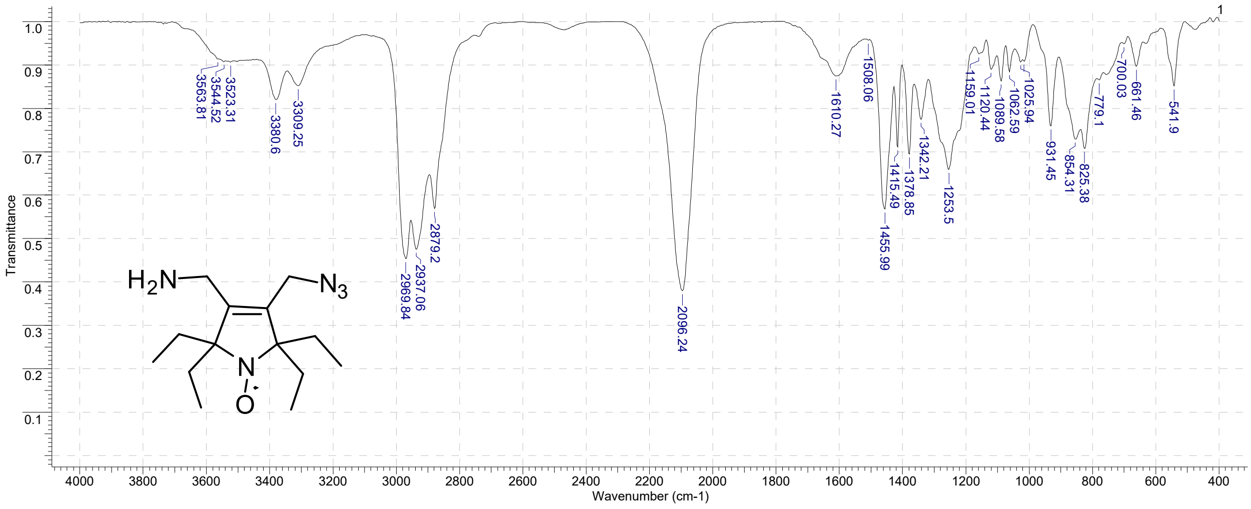

IR (neat) of 3-(aminomethyl)-4-((dimethylamino)methyl)-2,2,5,5-tetraethylpyrrolidin-1-oxyl (14a)

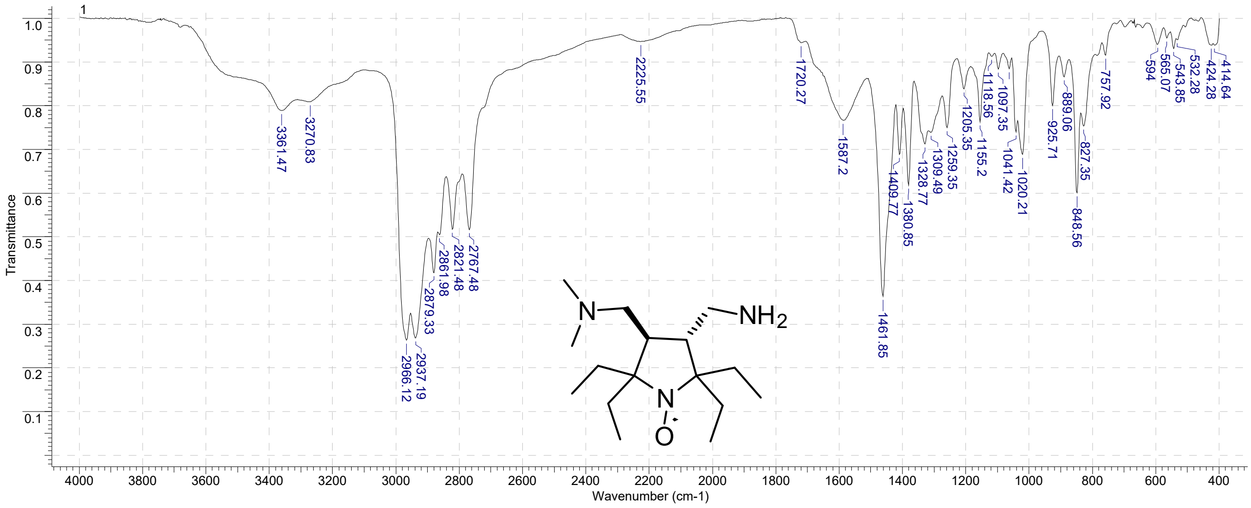

IR (neat) of 3-(aminomethyl)-4-((dimethylamino)methyl)-2,2,5,5-tetraethyl-2,5-dihydro-1H-pyrrol-1-oxyl (14b)

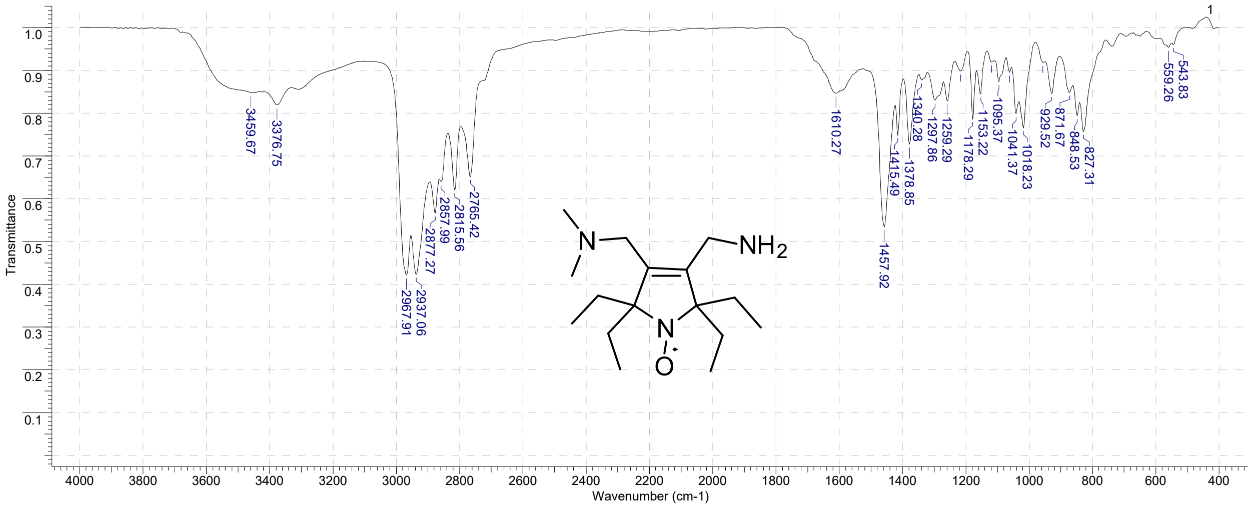

IR (KBr) of 3-((2,5-dioxo-2,5-dihydro-1H-pyrrol-1-yl)methyl)-2,2,5,5-tetraethyl-4-(hydroxymethyl)pyrrolidin-1-oxyl (7)

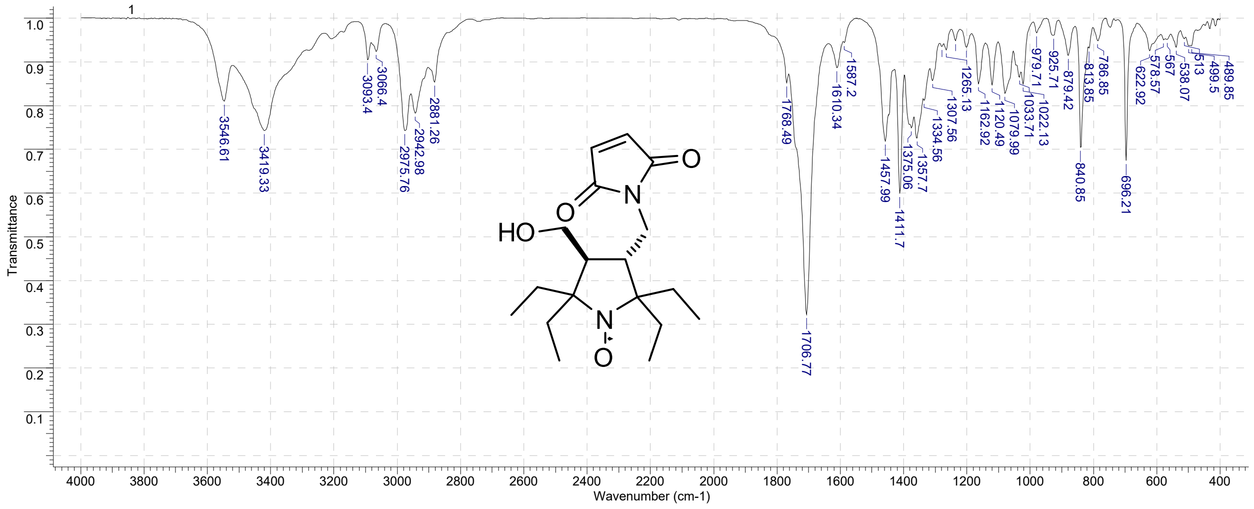

IR (KBr) of 3-((dimethylamino)methyl)-4-((2,5-dioxo-2,5-dihydro-1H-pyrrol-1-yl)methyl)-2,2,5,5-tetraethylpyrrolidin-1-oxyl (15a)

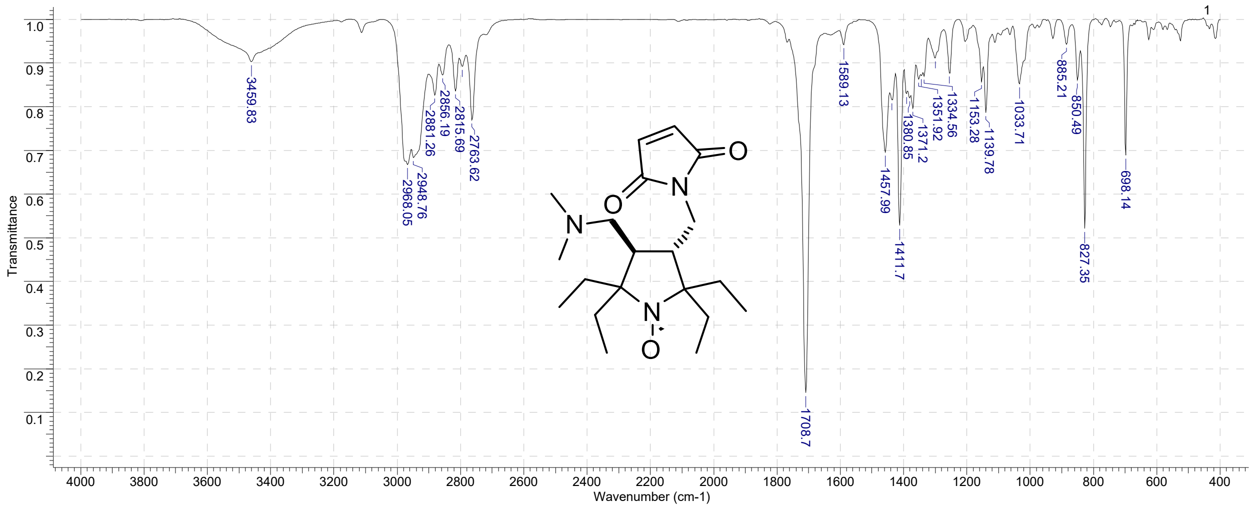

IR (KBr) of 3-((dimethylamino)methyl)-4-((2,5-dioxo-2,5-dihydro-1H-pyrrol-1-yl)methyl)-2,2,5,5-tetraethyl-2,5-dihydro-1H-pyrrol-1-oxyl (15b)

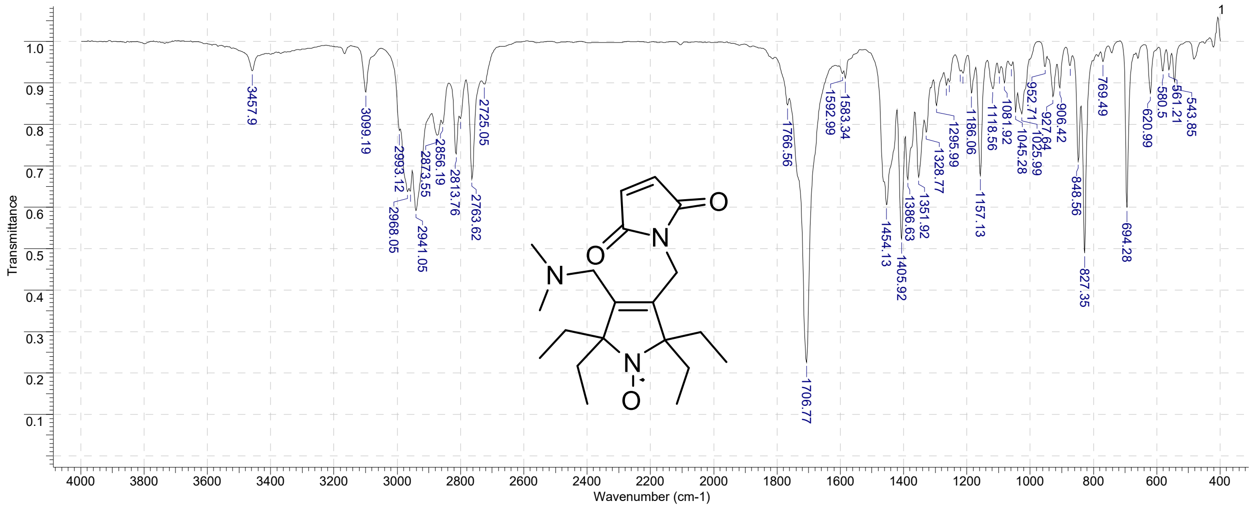

IR (KBr) of 3-(azidomethyl)-4-((4,5-dicarboxy-1H-1,2,3-triazol-1-yl)methyl)-2,2,5,5-tetraethylpyrrolidin-1-oxyl monosodium salt (20)

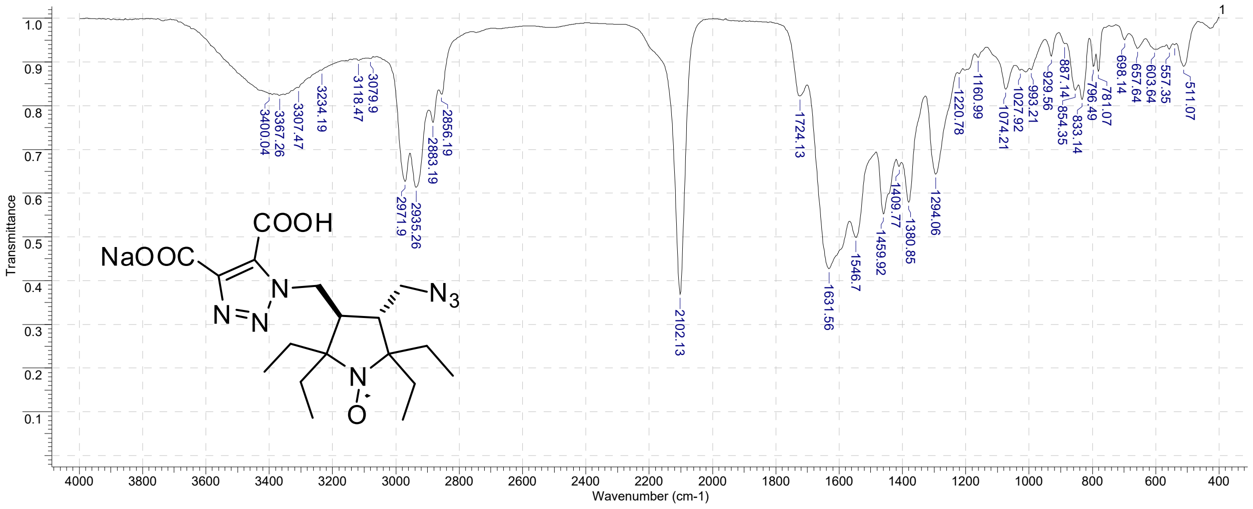

IR (KBr) of 3,4-bis(carboxymethyl)-2,2,5,5-tetraethylpyrrolidin-1-oxyl (23)

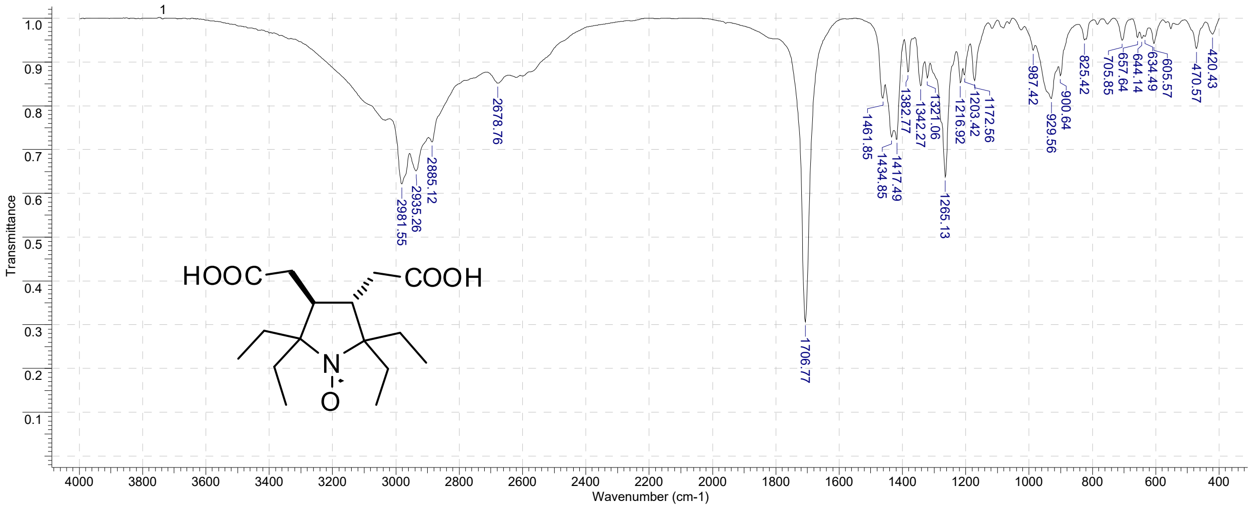

IR (KBr) of 3,4-bis(cyanomethyl)-2,2,5,5-tetraethylpyrrolidin-1-oxyl (**24**)

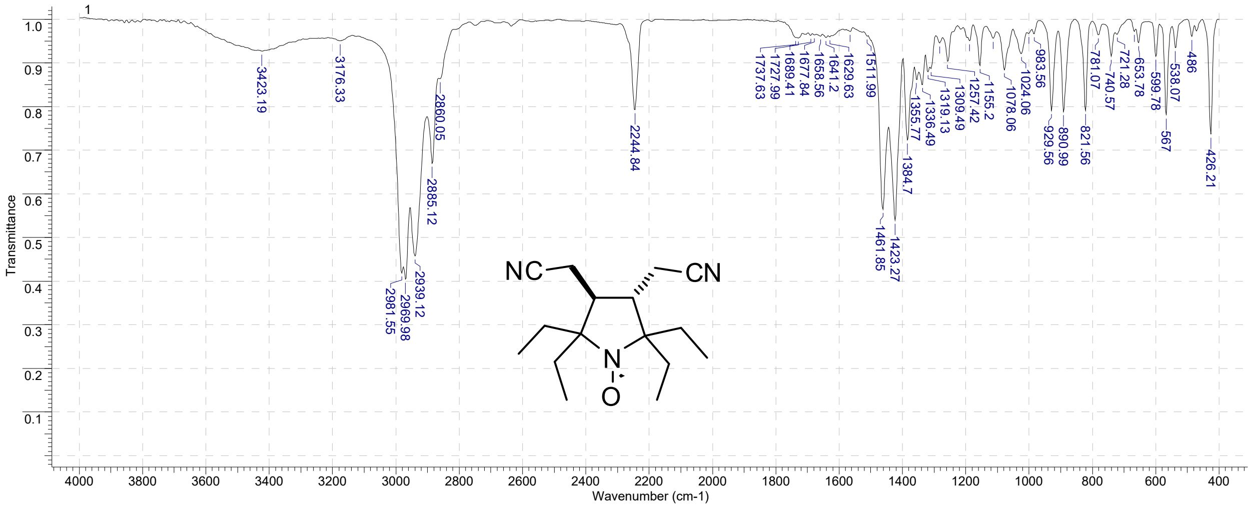

XRD Data

Table 1

XRD data for compounds **3, 4, 6, 8, 10, 12a, 15a, 15b, 20**.

Compound	**3**	**4**
Empirical formula	$C_{16}H_{32}NO_7S_2$	$C_{15}H_{30}NO_5S$
Formula weight	414.54	336.46
Temperature K	296(2)	296(2)
Wavelength Å	0.71073	0.71073
Crystal system	Tetragonal	Triclinic
Space group	$I4_1/a$	P-1
Unit cell dimensions *a* Å	11.2559(3)	9.1916(5)
b Å	11.2559(3)	13.3515(9)
c Å	34.1134(15)	14.9892(10)
α °	90	97.112(2)
β °	90	92.783(2)
γ °	90	93.154(2)
Volume $Å^3$	4322.0(3)	1819.7(2)
Z	8	4
Density (calcd.) $Mg.m^{-3}$	1.274	1.228
Abs. coefficient mm^{-1}	0.280	0.199
F(000)	1784	732
Crystal size mm^3	0.35 x 0.6 x 0.6	0.05 x 0.30 x 0.80
Θ range for data collection °	1.9 – 27.6	1.9 – 25.1
Index ranges	$-14 \leq h \leq 14$, $-14 \leq k \leq 14$, $-44 \leq l \leq 42$	$-10 \leq h \leq 10$, $-15 \leq k \leq 15$, $-17 \leq l \leq 17$
Reflections collected	27582	20336
Independent reflections	2506 R(int) = 0.042	6443 R(int)= 0.039
Completeness to θ %	99.8	99.6
Data / restraints / parameters	2606 / 0 / 122	6443/ 12 / 409
Goodness-of-fit on F^2	1.01	1.00
Final R indices $I > 2\sigma(I)$	R_1 = 0.0464, wR_2 = 0.1222	R_1 = 0.0698, wR_2 = 0.1848
Final R indices (all data)	R_1 = 0.0552, wR_2 = 0.1318	R_1 = 0.1227, wR_2 = 0.1978
Largest diff. peak / hole $e.Å^{-3}$	0.56/ -0.27	0.86/ -0.26
CCDC	2308349	2308350

Table 1 *(continued)*

Compound	**6**	**8**	**10**
Empirical formula	$C_{14}H_{29}N_2O_2$	$C_{19}H_{37}N_2O_4$	$C_{19}H_{36}N_5O_3$
Formula weight	257.39	357.50	382.53
Temperature K	296(2)	296(2)	200(2)
Wavelength Å	0.71073	0.71073	0.71073
Crystal system	Monoclinic	Monoclinic	Monoclinic
Space group	Cc	$P2_1/n$	Cc
Unit cell dimensions *a* Å	8.1003(10)	8.1312(3)	16.0849(11)
b Å	30.326(4)	24.0467(8)	12.2256(11)
c Å	13.2222(16)	12.0626(4)	12.2167(9)
α °	90	90	90
β °	106.333(4)	108.7839(17)	113.772(4)
γ °	90	90	90
Volume $Å^3$	3116.9(7)	2232.96(14)	2198.6(3)
Z	8	4	4
Density (calcd.) $Mg.m^{-3}$	1.097	1.063	1.156
Abs. coefficient mm^{-1}	0.073	0.074	0.079
F(000)	1144	788	836
Crystal size mm^3	0.20 x 0.40 x 0.80	0.07 x 0.12 x 0.68	0.10 x 0.30 x 0.35
Θ range for data collection °	1.3 – 26.1	2.7 – 25.1	2.2 – 25.0
Index ranges	$-10 \leq h \leq 9$, $-37 \leq k \leq 37$, $-16 \leq l \leq 16$	$-9 \leq h \leq 6$, $-28 \leq k \leq 28$, $-13 \leq l \leq 14$	$-18 \leq h \leq 19$, $-14 \leq k \leq 14$, $-14 \leq l \leq 13$
Reflections collected	22931	21652	10723
Independent reflections	6163 R(int) = 0.058	3970 R(int) = 0.059	3780 R(int) = 0.050
Completeness to θ %	99.6	99.5	97.2
Data / restraints / parameters	6163 / 2 / 336	3970 / 0 / 241	3780 / 39 / 250
Goodness-of-fit on F^2	1.10	0.99	1.04
Final R indices $I > 2\sigma(I)$	R_1 = 0.0713, wR_2 = 0.1709	R_1 = 0.0475, wR_2 = 0.1208	R_1 = 0.0793, wR_2 = 0.2009
Final R indices (all data)	R_1 = 0.0978, wR_2 = 0.1852	R_1 = 0.0634, wR_2 = 0.1320	R_1 = 0.1098, wR_2 = 0.2153
Largest diff. peak / hole $e.Å^{-3}$	0.26 / -0.35	0.20 / -0.15	0.77 / -0.29
CCDC	2309021	2308351	23082352

Table 1 *(continued)*

Compound	**12a**	**15a**	**15b**
Empirical formula	$C_{14}H_{26}N_7O$	$C_{20}H_{34}N_3O_3$	$C_{20}H_{32}N_3O_3$
Formula weight	308.42	364.50	357.50
Temperature K	296(2)	296(2)	296(2)
Wavelength Å	0.71073	0.71073	0.71073
Crystal system	Monoclinic	Orthorhombic	Monoclinic
Space group	$P2_1/n$	$P2_12_12_1$	$P2_1$
Unit cell dimensions *a* Å	15.2867(11)	8.0517(11)	9.063(2)
b Å	7.7128(5)	9.9408(12)	10.668(2)
c Å	15.5815(12)	26.754(3)	11.401(3)
α °	90	90	90
β °	112.088(3)	90	110.718(8)
γ °	90	90	90
Volume $Å^3$	1702.3(2)	2141.4(5)	1031.1(4)
Z	4	4	2
Density (calcd.) $Mg.m^{-3}$	1.203	1.131	1.168
Abs. coefficient mm^{-1}	0.082	0.076	0.079
F(000)	668	796	788
Crystal size mm^3	0.08 x 0.15 x 0.55	0.22 x 0.40 x 0.64	0.20 x 0.40 x 0.80
Θ range for data collection °	2.4 - 26.1	2.6 – 25.6	2.75 – 26.1
Index ranges	$-18 \leq h \leq 18$, $-9 \leq k \leq 9$, $-19 \leq l \leq 19$	$-7 \leq h \leq 9$, $-10 \leq k \leq 12$, $-32 \leq l \leq 29$	$-11 \leq h \leq 11$, $-13 \leq k \leq 13$, $-13 \leq l \leq 14$
Reflections collected	21917	12566	8396
Independent reflections	3367 R(int)= 0.040	3993 R(int) = 0.051	3973 R(int) = 0.064
Completeness to θ %	99.7	99.7	99.8
Data / restraints / parameters	3367 / 0 / 203	3993 / 0 / 235	3973 / 1 / 241
Goodness-of-fit on F^2	1.04	1.09	1.01
Final R indices $I > 2\sigma(I)$	R_1 = 0.0541, wR_2 = 0.1327	R_1 = 0.0673, wR_2 = 0.1779	R_1 = 0.0588, wR_2 = 0.1538
Final R indices (all data)	R_1 = 0.0895, wR_2 = 0.1530	R_1 = 0.0916, wR_2 = 0.1917	R_1 = 0.0779, wR_2 = 0.1724
Largest diff. peak / hole $e.Å^{-3}$	0.25 / -0.17	0.22 / -0.23	0.36 / -0.23
CCDC	2308353	2308354	2308355

Compound	**20**
Empirical formula	$C_{18}H_{28}N_7O_7Na$
Formula weight	477.46
Temperature K	200(2)
Wavelength Å	0.71073
Crystal system	Monoclinic
Space group	$P2_1/n$
Unit cell dimensions *a* Å	12.0326(6)
b Å	10.6711(4)
c Å	18.4973(8)
α °	90
β °	92.254(2)
γ °	90
Volume $Å^3$	2373.24(18)
Z	4
Density (calcd.) $Mg.m^{-3}$	1.336
Abs. coefficient mm^{-1}	0.119
F(000)	1008
Crystal size mm^3	0.10 x 0.40 x 0.50
Θ range for data collection °	2.1 – 27.6
Index ranges	$-15 \leq h \leq 15$, $-13 \leq k \leq 13$, $-19 \leq l \leq 24$
Reflections collected	37337
Independent reflections	5462 R(int) = 0.053
Completeness to θ %	99.9
Data / restraints / parameters	5462 / 18 / 342
Goodness-of-fit on F^2	1.05
Final R indices $I > 2\sigma(I)$	R_1 = 0.069, wR_2 = 0.1726
Final R indices (all data)	R_1 = 0.1018, wR_2 = 0.1936
Largest diff. peak / hole $e.Å^{-3}$	1.68 / -0.43
CCDC	2308356

EPR Spectra

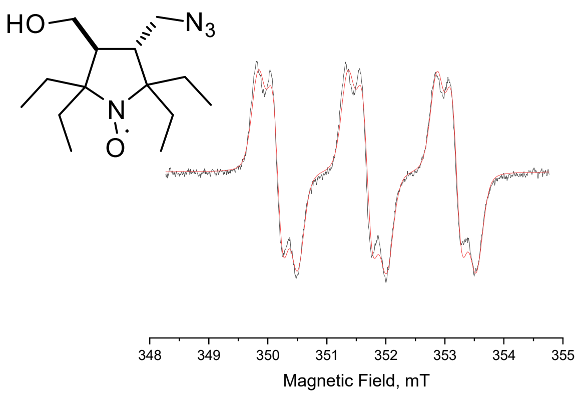

EPR of 3-(Azidomethyl)-2,2,5,5-tetraethyl-4-(hydroxymethyl)pyrrolidin-1-oxyl (5)

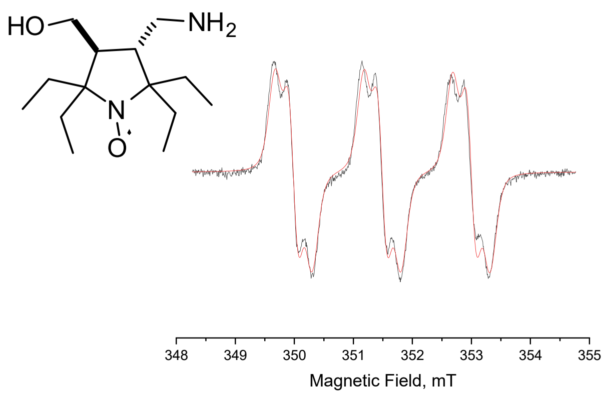

EPR of 3-(Aminomethyl)-2,2,5,5-tetraethyl-4-(hydroxymethyl)pyrrolidin-1-oxyl (6)

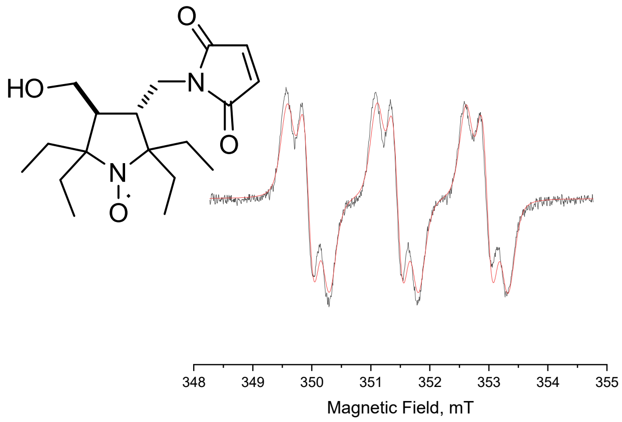

EPR of 3-((2,5-dioxo-2,5-dihydro-1*H*-pyrrol-1-yl)methyl)-2,2,5,5-tetraethyl-4-(hydroxymethyl)pyrrolidin-1-oxyl (7)

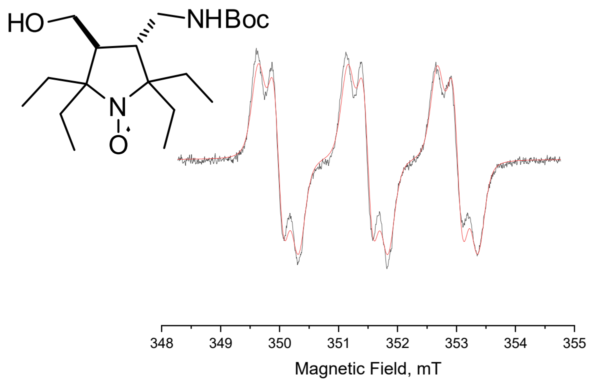

EPR of 3-(((tert-Butoxycarbonyl)amino)methyl)-2,2,5,5-tetraethyl-4-(hydroxymethyl)pyrrolidin-1-oxyl (8)

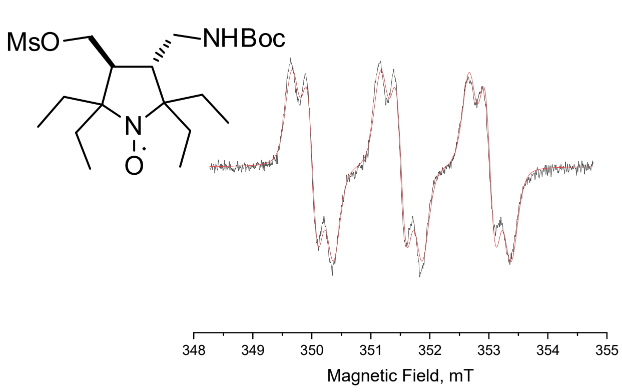

EPR of 3-(((tert-Butoxycarbonyl)amino)methyl)-2,2,5,5-tetraethyl-4-(((methylsulfonyl)oxy)methyl)pyrrolidin-1-oxyl (9)

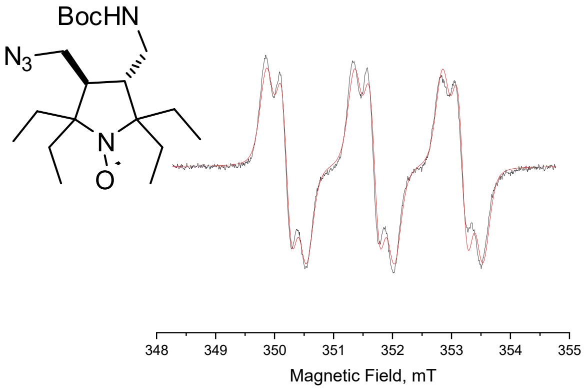

EPR of 3-(Azidomethyl)-4-((tert-butoxycarbonylamino)methyl)-2,2,5,5-tetraethylpyrrolidin-1-oxyl (10)

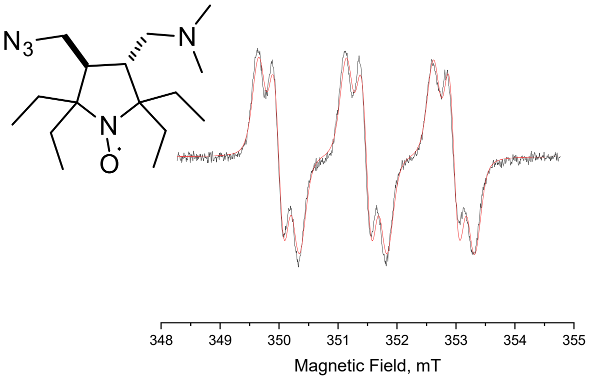

EPR of 3-(azidomethyl)-4-((dimethylamino)methyl)-2,2,5,5-tetraethylpyrrolidin-1-oxyl (11a)

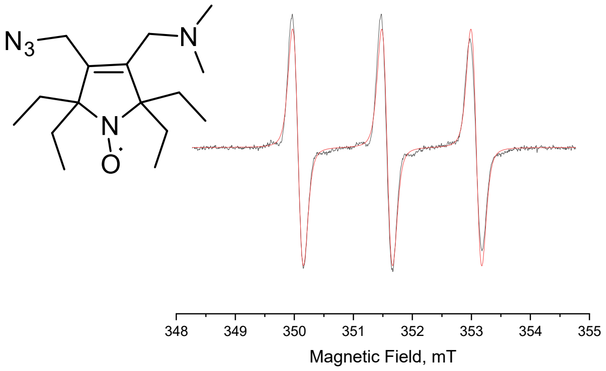

EPR of 3-(azidomethyl)-4-((dimethylamino)methyl)-2,2,5,5-tetraethyl-2,5-dihydro-1H-pyrrol-1-oxyl (11b)

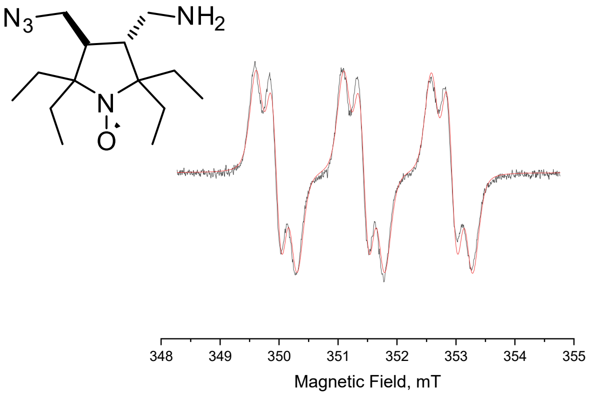

EPR of 3-(aminomethyl)-4-(azidomethyl)-2,2,5,5-tetraethylpyrrolidin-1-oxyl (13a)

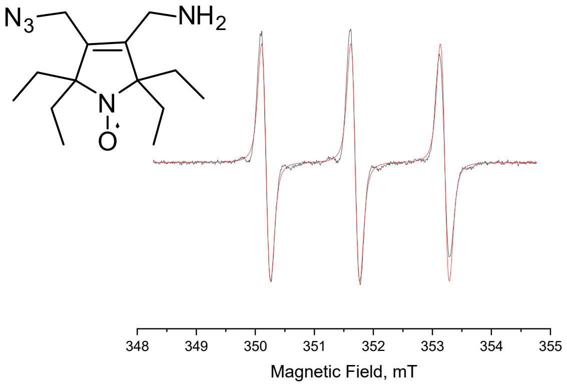

EPR of 3-(aminomethyl)-4-(azidomethyl)-2,2,5,5-tetraethyl-2,5-dihydro-1H-pyrrol-1-oxyl (13b)

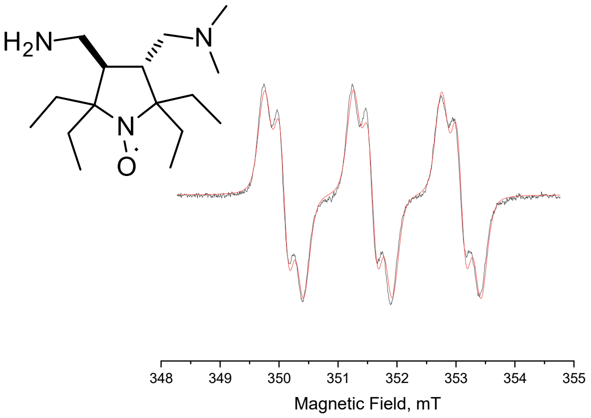

EPR of 3-(aminomethyl)-4-((dimethylamino)methyl)-2,2,5,5-tetraethylpyrrolidin-1-oxyl (14a)

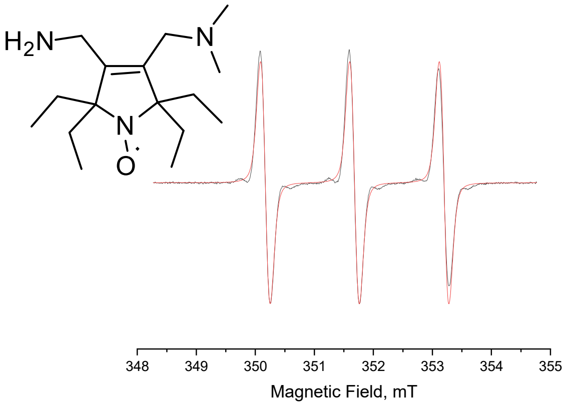

EPR of 3-(aminomethyl)-4-((dimethylamino)methyl)-2,2,5,5-tetraethyl-2,5-dihydro-1H-pyrrol-1-oxyl (14b)

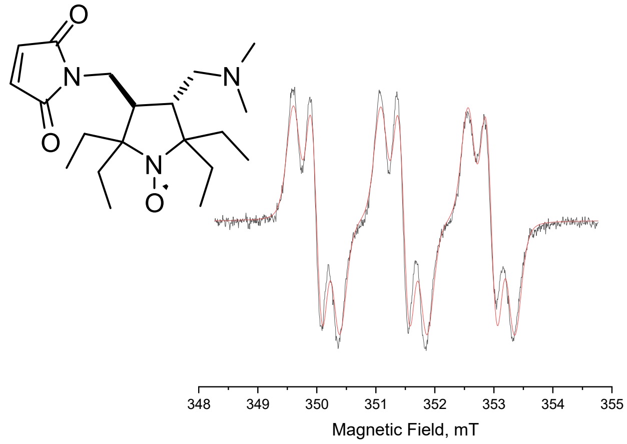

EPR of 3-((dimethylamino)methyl)-4-((2,5-dioxo-2,5-dihydro-1H-pyrrol-1-yl)methyl)-2,2,5,5-tetraethylpyrrolidin-1-oxyl (15a)

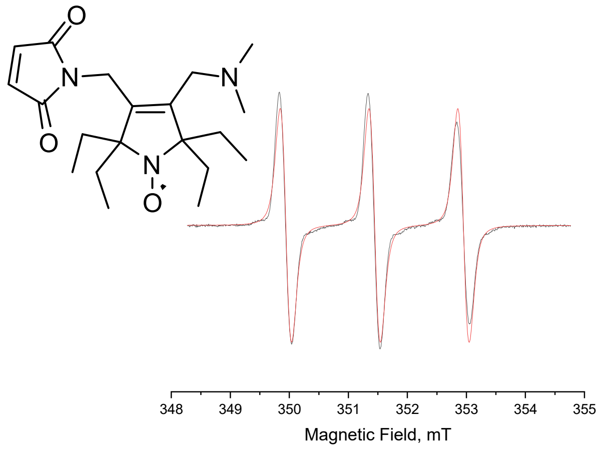

EPR of 3-((dimethylamino)methyl)-4-((2,5-dioxo-2,5-dihydro-1H-pyrrol-1-yl)methyl)-2,2,5,5-tetraethyl-2,5-dihydro-1H-pyrrol-1-oxyl (15b)

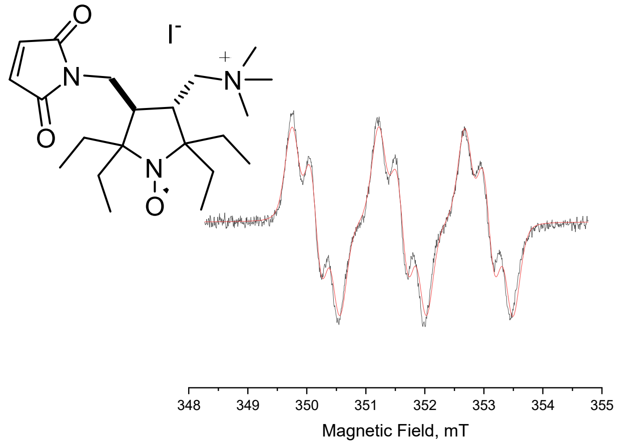

EPR of 3-((2,5-Dioxo-2,5-dihydro-1H-pyrrol-1-yl)methyl)-2,2,5,5-tetraethyl-4-((trimethylammonio)methyl)pyrrolidin-1-oxyl monoiodide (16a)

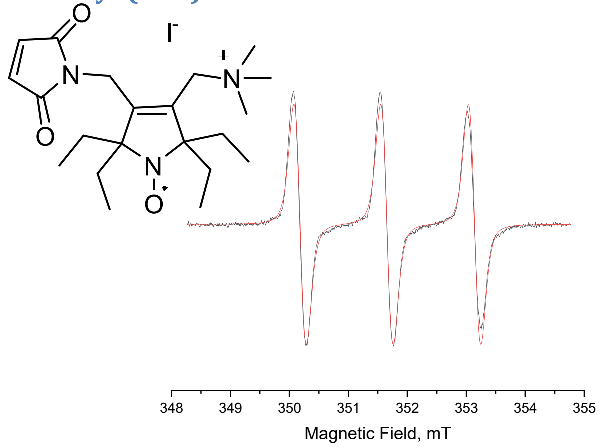

EPR of 3-((2,5-Dioxo-2,5-dihydro-1H-pyrrol-1-yl)methyl)-2,2,5,5-tetraethyl-4-((trimethylammonio)methyl)-2,5-dihydro-1H-pyrrol-1-oxyl monoiodide (16b)

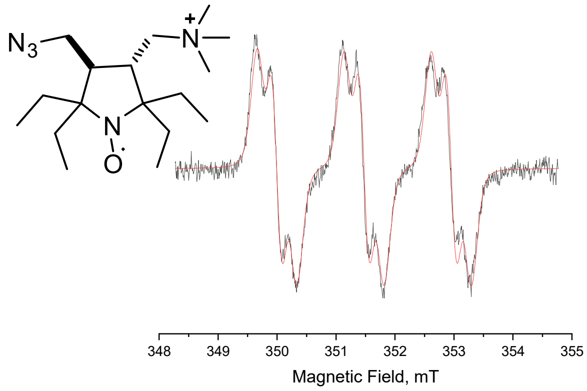

EPR of 3-(azidomethyl)-2,2,5,5-tetraethyl-4-((trimethylammonio)methyl)pyrrolidin-1-oxyl monoiodide (17a)

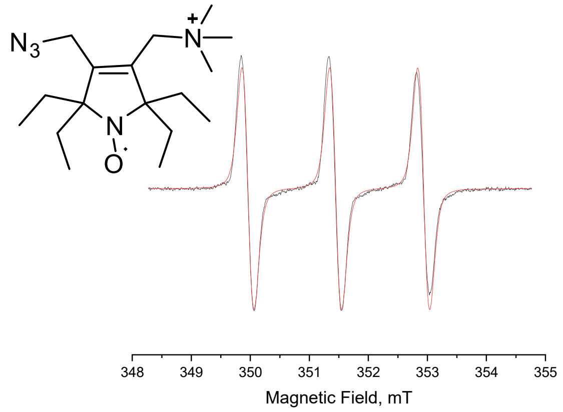

EPR of 3-(azidomethyl)-2,2,5,5-tetraethyl-4-((trimethylammonio)methyl)-2,5-dihydro-1H-pyrrol-1-oxyl monoiodide (17b)

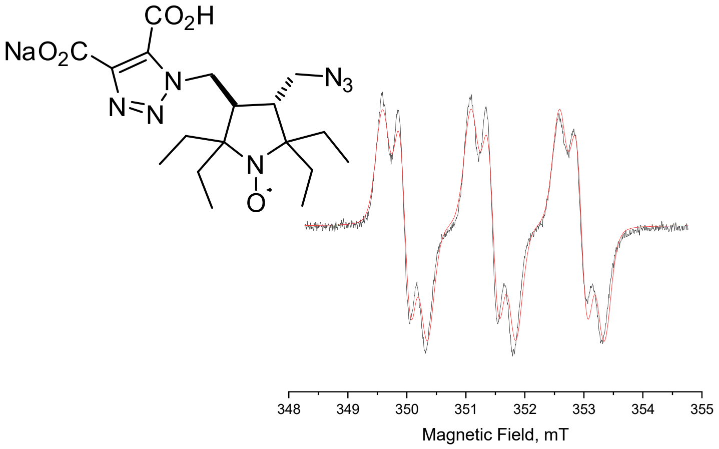

EPR of 3-(azidomethyl)-4-((4,5-dicarboxy-1H-1,2,3-triazol-1-yl)methyl)-2,2,5,5-tetraethylpyrrolidin-1-oxyl monosodium salt (20)

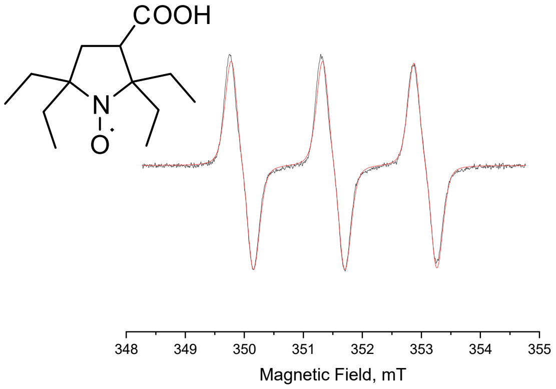

EPR of 3-carboxy-2,2,5,5-tetraethylpyrrolidin-1-oxyl (21)

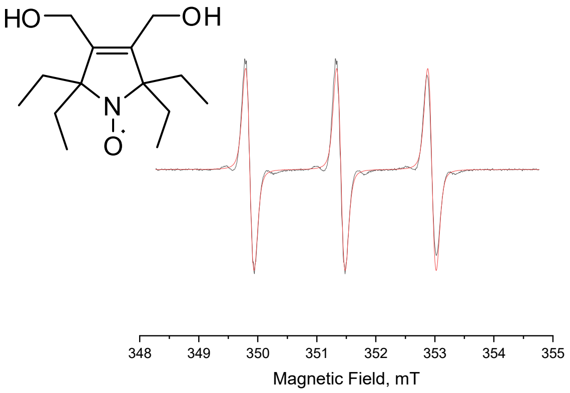

EPR of 3,4-bis(hydroxymethyl)-2,2,5,5-tetraethyl-2,5-dihydro-1H-pyrrol-1-oxyl (22)

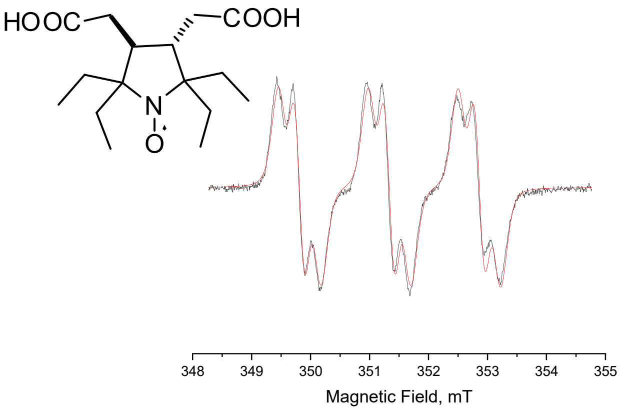

EPR of 3,4-bis(carboxymethyl)-2,2,5,5-tetraethylpyrrolidin-1-oxyl (23)
